# Translating state-of-the-art spinal cord MRI techniques to clinical use: A systematic review of clinical studies utilizing DTI, MT, MWF, MRS, and fMRI

**DOI:** 10.1016/j.nicl.2015.11.019

**Published:** 2015-12-04

**Authors:** Allan R. Martin, Izabela Aleksanderek, Julien Cohen-Adad, Zenovia Tarmohamed, Lindsay Tetreault, Nathaniel Smith, David W. Cadotte, Adrian Crawley, Howard Ginsberg, David J. Mikulis, Michael G. Fehlings

**Affiliations:** a Division of Neurosurgery, Department of Surgery, University of Toronto, Toronto, Ontario, Canada; b Polytechnique Montreal, Montreal, Quebec, Canada; c Royal College of Surgeons in Ireland, Dublin, Ireland; d McMaster University, Hamilton, Ontario, Canada; e Department of Medical Imaging, University of Toronto, Toronto, Ontario, Canada

**Keywords:** MRI, diffusion tensor imaging, DTI, magnetization transfer, MT, T2*-weighted imaging, spinal cord, spine, cervical spine, myelopathy, degenerative cervical myelopathy, DCM, cervical spondylotic myelopathy, CSM, multiple sclerosis, MS, amyotrophic lateral sclerosis, ALS, spinal cord injury, SCI

## Abstract

**Background:**

A recent meeting of international imaging experts sponsored by the International Spinal Research Trust (ISRT) and the Wings for Life Foundation identified 5 state-of-the-art MRI techniques with potential to transform the field of spinal cord imaging by elucidating elements of the microstructure and function: diffusion tensor imaging (DTI), magnetization transfer (MT), myelin water fraction (MWF), MR spectroscopy (MRS), and functional MRI (fMRI). However, the progress toward clinical translation of these techniques has not been established.

**Methods:**

A systematic review of the English literature was conducted using MEDLINE, MEDLINE-in-Progress, Embase, and Cochrane databases to identify all human studies that investigated utility, in terms of diagnosis, correlation with disability, and prediction of outcomes, of these promising techniques in pathologies affecting the spinal cord. Data regarding study design, subject characteristics, MRI methods, clinical measures of impairment, and analysis techniques were extracted and tabulated to identify trends and commonalities. The studies were assessed for risk of bias, and the overall quality of evidence was assessed for each specific finding using the Grading of Recommendations Assessment, Development and Evaluation (GRADE) framework.

**Results:**

A total of 6597 unique citations were identified in the database search, and after full-text review of 274 articles, a total of 104 relevant studies were identified for final inclusion (97% from the initial database search). Among these, 69 studies utilized DTI and 25 used MT, with both techniques showing an increased number of publications in recent years. The review also identified 1 MWF study, 11 MRS studies, and 8 fMRI studies. Most of the studies were exploratory in nature, lacking a priori hypotheses and showing a high (72%) or moderately high (20%) risk of bias, due to issues with study design, acquisition techniques, and analysis methods. The acquisitions for each technique varied widely across studies, rendering direct comparisons of metrics invalid. The DTI metric fractional anisotropy (FA) had the strongest evidence of utility, with moderate quality evidence for its use as a biomarker showing correlation with disability in several clinical pathologies, and a low level of evidence that it identifies tissue injury (in terms of group differences) compared with healthy controls. However, insufficient evidence exists to determine its utility as a sensitive and specific diagnostic test or as a tool to predict clinical outcomes. Very low quality evidence suggests that other metrics also show group differences compared with controls, including DTI metrics mean diffusivity (MD) and radial diffusivity (RD), the diffusional kurtosis imaging (DKI) metric mean kurtosis (MK), MT metrics MT ratio (MTR) and MT cerebrospinal fluid ratio (MTCSF), and the MRS metric of *N*-acetylaspartate (NAA) concentration, although these results were somewhat inconsistent.

**Conclusions:**

State-of-the-art spinal cord MRI techniques are emerging with great potential to improve the diagnosis and management of various spinal pathologies, but the current body of evidence has only showed limited clinical utility to date. Among these imaging tools DTI is the most mature, but further work is necessary to standardize and validate its use before it will be adopted in the clinical realm. Large, well-designed studies with a priori hypotheses, standardized acquisition methods, detailed clinical data collection, and robust automated analysis techniques are needed to fully demonstrate the potential of these rapidly evolving techniques.

## 1 Background

The advent of magnetic resonance imaging (MRI) in the mid-1980s transformed the field of spinal cord imaging and provided clinicians with high-resolution anatomical images, directly leading to improved clinical decision-making. Conventional MRI techniques (spin echo, gradient echo, and inversion recovery sequences, with T1-, T2-, or proton density-weighting) have continued to mature over 3 decades of use, establishing MRI as the imaging modality of choice for most spinal disorders. However, conventional MRI provides little information regarding the health and integrity of the spinal cord tissue itself, due to the fact that signal intensity changes are non-specific and do not correspond directly with aberrant physiological processes (Wada et al., 1995). This is reflected in the poor correlation of conventional MRI data with neurological and functional impairment in various spinal cord pathologies (Tetreault et al., 2013, Wilson et al., 2012), and failure to provide reliable prognostic information. In the degenerative condition cervical spondylotic myelopathy (CSM), weak correlates with clinical status have been identified using T2-weighted hyper-intensity (T2w-HI), T1-weighted (T1w) hypo-intensity, and measures of cord compression (Matsuda et al., 1999, Tetreault et al., 2013, Wada et al., 1995). In multiple sclerosis (MS), numerous studies have found that spinal cord lesion load is less important than atrophy, measured as the cross-sectional area (CSA) of the cord (Stevenson et al., 1998). As a result, conventional MRI techniques are of limited value in developing imaging biomarkers or predicting clinical outcomes because they are not sensitive and specific measures of the degenerative and regenerative changes that occur within the spinal cord at the microstructural and functional levels.

A 2013 international meeting of spinal cord imaging experts, sponsored by the International Spinal Research Trust (ISRT) and the Wings for Life (WfL) Spinal Cord Research Foundation, outlined 5 emerging MRI techniques that have the potential to revolutionize the field, by elucidating details of the microstructure and functional organization within the spinal cord (Stroman et al., 2014, Wheeler-Kingshott et al., 2014). This group highlighted the following techniques due to their ability to characterize microstructural features of the spinal cord: diffusion tensor imaging (DTI), magnetization transfer (MT), myelin water-fraction (MWF), and magnetic resonance spectroscopy (MRS). DTI measures the directional diffusivity of water, and several of the metrics that it produces correlate with axonal integrity, and to a lesser degree, myelination (Wheeler-Kingshot et al., 2002). MT involves an off-resonance saturating pre-pulse that takes advantage of the chemical and magnetization exchange between protons bound to lipid macromolecules and nearby water protons, and provides a surrogate measure of myelin quantity (Graham and Henkelman, 1997). This is most often expressed in a ratio between scans with and without the pre-pulse (MTR) or between the spinal cord and cerebrospinal fluid (MTCSF). MWF estimates the fraction of tissue water bound to the myelin sheath, by fitting the T2 relaxation curve to a multi-exponential model and identifying the fraction of the signal with a T2 parameter between 15 and 40 ms (Wu et al., 2006). MRS quantifies either the absolute or relative concentrations of specific molecules of interest within a single large voxel, including *N*-acetylaspartate (NAA), myo-inositol (Ins), choline (Cho), creatine (Cre), and lactate (Lac) (Gomez-Anson et al., 2000). The expert panel also highlighted functional MRI (fMRI) of the spinal cord, due to its potential to characterize changes in neurological function, using either blood oxygen-level dependent (BOLD), which relies upon the concept of neuro-vascular coupling in which changes in neurological function produce corresponding changes in local blood flow, or signal enhancement by extravascular protons (SEEP), which is thought to detect neural activity indirectly through changes in the intracellular/extracellular volume ratio (Stroman et al., 2001). fMRI studies can involve a variety of designs, including motor tasks or sensory stimuli in block or event-related designs, and can visualize and provide indirect measures reflecting neuronal activity and connectivity occurring within the spinal cord (Stroman et al., 2014).

All 5 of these emerging MRI techniques are highly amenable to quantitative analysis, offering the opportunity to develop quantitative MRI biomarkers that correlate with disability and/or predict outcomes. The development of these techniques may also provide more sensitive and specific diagnostic tests. For example, in the earliest stages of CSM, symptoms may include vague complaints of numbness and neck pain, but the cause may be unclear between early myelopathy vs. musculoskeletal pain and peripheral nerve compression. Objective evidence of damage to the cord tissue could provide important information to prompt earlier surgery. Furthermore, quantitative biomarkers could act as surrogate outcome measures in clinical trials, such as therapeutic remyelination agents in MS or spinal cord injury (SCI), providing short-term end-points and reducing the time and costs associated with novel drug development (Cadotte and Fehlings, 2013). In acute SCI, these techniques could potentially discriminate reversible and irreversible components of damage (demyelination, axonal loss, gray matter loss) early after injury, and thus provide a more accurate prognosis to help guide therapeutic strategies and focus rehabilitation resources.

Unfortunately, the application of these advanced MRI techniques to image the spinal cord is far from trivial. These techniques were initially developed and validated in brain imaging, but the spinal cord is a far more challenging structure to obtain accurate data. In fact, the spine is among the most hostile environments in the body for MRI, due to magnetic field inhomogeneity at the interfaces between bone, intervertebral disk, and cerebrospinal fluid (CSF), and also because of the small size of the cord and its white matter tracts, and the relatively large motion of the cord during cardiac and respiratory cycles (Stroman et al., 2014). High-quality spinal cord imaging using these methods has only recently been achieved, requiring specialized acquisition sequences, complex shimming, custom receive coils, long acquisition times, and substantial post-processing to correct for motion, aliasing, and other artifacts.

This systematic review aims to summarize the progress of clinical translation of these imaging techniques to date, and identify the most common technical methods employed. The review will also highlight the major barriers that are currently preventing the adoption of these techniques into clinical use. The search was designed to identify all studies that applied one or more of these MRI techniques to assess for clinical utility in one or more of the following 3 key questions:

1. *Diagnostic utility*: Does the MRI technique provide metrics that demonstrate group differences or improved diagnostic accuracy (sensitivity/specificity) in the diagnosis of spinal pathologies?
2. *Biomarker utility*: Does the advanced MRI technique generate metrics that quantify the amount of injury and thus correlate with neurological/functional impairment and/or show longitudinal changes over time that correlate with changes in disability in spinal pathologies?
3. *Predictive utility*: Does the advanced MRI technique generate metrics that predict neurological, functional, or quality of life outcomes in spinal pathologies?

## 2 Methods

### 2.1 Electronic literature search

**Fig. 1:**
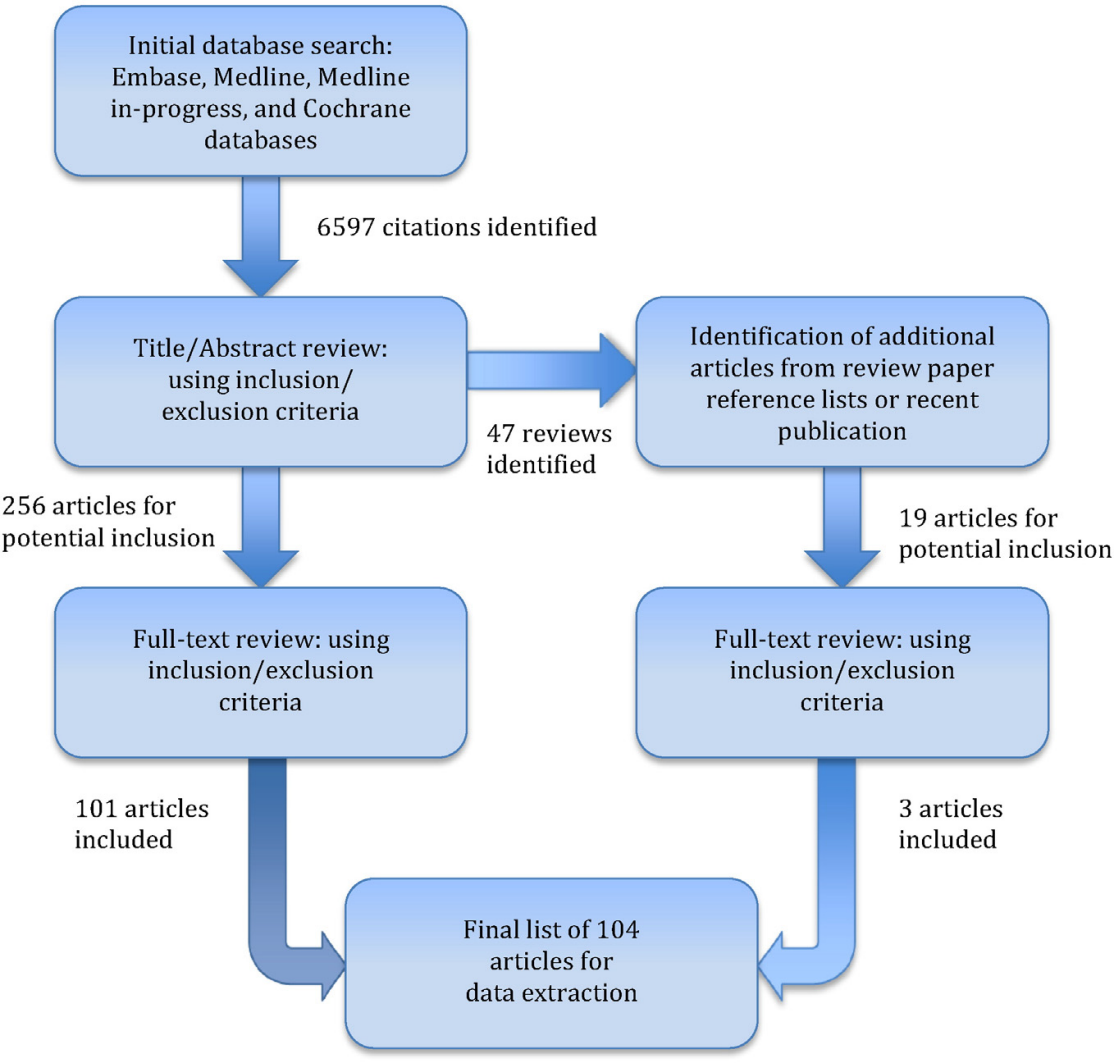
Flowchart showing results of literature search.

A systematic search of MEDLINE, MEDLINE-in-Progress, Embase, and Cochrane databases was conducted, with the results formatted in accordance with the PRISMA statement for systematic reviews and meta-analyses (Liberati et al., 2009). The search included literature published from January 1, 1985 to June 1, 2015 and sought all studies that describe the use of one or more of the state-of-the-art spinal cord MRI techniques (DTI, MT, MWF, MRS, and fMRI) on subjects with any clinical pathology (complete search terms listed in Appendix A, inclusion/exclusion criteria in Table 1). Studies that employed diffusion kurtosis imaging (DKI), an extension of DTI using multiple b-values, were included as these studies typically also report DTI metrics in addition to measures of kurtosis. Studies that employed advanced MRI techniques to image only the brain were excluded (e.g. brain MRS in CSM). We also excluded studies utilizing diffusion-weighted imaging (DWI) that only calculated an apparent diffusion coefficient, but did not calculate tensors (which require the use of diffusion-sensitizing gradients in at least 6 directions) or tensor-derived metrics such as fractional anisotropy (FA), axial diffusivity (AD), and radial diffusivity (RD). The search was limited to human studies, but limits on study design were not placed. Abstracts identified in the initial search were reviewed by 3 of the authors (A.R.M., I.A., N.S.) to determine relevant manuscripts for full-text review. The inclusion criteria required that studies were original research that appeared to answer one or more of the key questions above and included a minimum of 24 total subjects, with at least 12 of these subjects with a specific spinal pathology. Thus, we included studies with at least 24 pathological subjects (with no control subjects), and studies with at least 12 pathological subjects and a total of at least 24 subjects (including controls). Studies that included 3 or more different groups for comparison (e.g. NMO vs. MS vs. healthy) were required to have at least 12 subjects with the primary pathology of interest. Case reports or smaller series, meeting abstracts, white papers, editorials, review papers, technical reports, or studies of only healthy subjects were excluded. The full text of each article was then analyzed by 2 of the authors (A.R.M., I.A.) in the context of each key question to determine suitability for final inclusion, with discrepancies resolved by discussion. If multiple articles were identified with redundant results based on the same group of subjects, only the most relevant article (larger sample size or more recent publication) was kept in the review. References of each full-text article and each review paper that were identified were also systematically checked to identify additional eligible articles (Fig. 1).

For key question 1 (diagnostic utility) we sought all articles that compared the presence or absence of a specific MRI feature or the value of a quantitative metric between patients and controls, relating to diagnosis. For question 2 (biomarker utility), we identified articles that identified relationships between MRI metrics and measures of clinical disability, including the calculation of correlation coefficients (Pearson, Spearman, or multivariate) or identification of differences between severity groups. To be relevant to key question 3 (predictive utility), studies needed to assess the relationship between baseline MRI metrics and follow-up clinical data at a specified time at least 3 months after the initial imaging.

### 2.2 Data extraction

For each of the articles that met all inclusion/exclusion criteria after full-text review, the following data were extracted redundantly by 2 of the authors (A.R.M., Z.T.): study design, subject characteristics (age, gender, diagnosis, treatment(s) administered), follow-up duration, MRI sequences, MRI acquisition parameters, MRI data analysis methods, clinical data recorded, and results pertaining to diagnosis, correlation with disability, and correlation with outcomes. Differences in extracted data were resolved by discussion.

### 2.3 Data analysis and synthesis

Regarding diagnosis, we analyzed group differences and their statistical significance (P-value), and also the number of subjects with each specific MRI feature, present or absent (or a quantity above/below a threshold), that was reported for pathological and healthy subjects, to assess sensitivity (SE), specificity (SP), positive predictive value (PPV), and negative predictive value (NPV). For correlations with disability and prediction of clinical outcomes, we collected results that were reported as odds ratios, univariate or multivariate correlation coefficients, and P-values.

Although many of the studies identified in this systematic review reported results using the same quantitative metrics, a formal meta-analysis was not performed due to the wide variation in acquisition and data analysis techniques. Such a meta-analysis would only be relevant for a group of studies that showed substantial homogeneity in subject populations, MRI techniques, regions of interest (ROIs), and clinical measures. However, trends in the data were tabulated and summarized independently by 2 authors (A.R.M., I.A.) and discrepancies were resolved by discussion.

### 2.4 Risk of bias for individual studies

Risk of bias was assessed for each article independently by 2 reviewers (A.R.M., I.A.). The risk of bias criteria were defined by the authors by consensus, combining criteria from the Center for Evidence-Based Medicine (CEBM) Diagnostic Study Appraisal Worksheet (CEBM Website) and *The Journal of Bone & Joint Surgery* for prognostic studies (Wright et al., 2003), in addition to the modifications described in Skelly et al. (2013). The criteria were further modified to also consider potential sources of bias related to technical factors. The criteria are summarized in Table 2. Factors that were considered to be potential sources of bias include retrospective, case series, or case–control study designs; failure to match or analyze differences in demographics (age, gender) or control for other confounders; heterogeneity in the diagnosis of the study population; non-random enrollment methods (e.g. convenience sampling or posters may have increased selection bias compared with consecutive enrollment); unreliable acquisition and analysis methods; and a narrow range of severity of illness. More specifically, acquisition techniques were considered to have a higher risk of bias if they produced wide confidence intervals for metrics (> 20%), showed distortions/artifacts that frequently required the exclusion of slices/subjects (> 5%), or were subject to potential systematic bias, such as acquisitions that have substantial partial volume effects due to in-plane resolution > 1.5 × 1.5 mm^2^, or thickness > 5 mm. Analytical techniques were considered to confer a higher risk of bias if they involved manual processes (e.g. ROI selection) without blinding, or liberal statistical assumptions (e.g. uncorrected p < 0.05 for activations in fMRI). For diagnostic studies, failure to calculate and report diagnostic accuracy was considered a potential source of reporting bias, as it conceals how many pathological subjects have an “abnormal” result on a given metric. Similarly, correlation studies that did not publish univariate or multivariate correlation coefficients do not disclose the strength of the correlation. Prognostic studies were also judged to have potential bias if the patients were not at a similar point in the course of disease (lacking internal validity), if the study did not achieve > 80% clinical follow-up, if follow-up was not long enough for a majority of patients to show a clinical change, or if other known prognostic factors were not reported and analyzed. If an article failed to report important information for any of the aforementioned potential sources of bias, or technical details that are necessary to reproduce the image acquisition, it was considered to have an increased risk of bias. Following rating of each article for risk of bias by the 2 reviewers, discrepancies were resolved by discussion.

### 2.5 Overall quality of the body of literature

After individual article evaluation, the overall body of evidence with respect to each key question and specific finding was determined based upon precepts outlined by the Grading of Recommendation Assessment, Development and Evaluation (GRADE) Working Group (Schünemann et al., 2008). The possible ratings for overall quality of evidence are high, moderate, low, very low, and insufficient. The initial quality of the overall body of evidence was considered high if the majority of the studies had low or moderately low risk of bias, and low if the majority of the studies had high or moderately high risk of bias. The body of evidence was then upgraded 1 or 2 levels (only if no downgrading occurred) on the basis of the following criteria: (1) large magnitude of effect or (2) dose–response gradient, or downgraded 1 or 2 levels on the basis of the following criteria: (1) inconsistency of results, (2) indirectness of evidence, (3) imprecision of the effect estimates (e.g., wide confidence intervals [CIs] > 50% of the estimate), or (4) non-a priori statement of subgroup analyses. The final overall quality of evidence expresses our confidence in the estimate of effect and the impact that further research may have on the results (Schünemann et al., 2008). The overall quality reflects the authors' confidence that the evidence reflects the true effect and the likelihood that further research will not change this estimate of effect. For example, a high level of evidence suggests that the evidence reflects the true effect, and further research is very unlikely to change our confidence in the estimate. A grade of “insufficient” means that evidence either is unavailable or does not permit a conclusion.

## 3 Results

### 3.1 Study selection

The literature search was designed to be highly inclusive and generated a total of 6597 unique citations (Fig. 1). Following review of the title and abstract, 256 articles were retained for full-text review and 47 review papers were identified. The full-text review of the 256 articles excluded another 156, leaving 101 articles that met all inclusion/exclusion criteria and were relevant to one or more of the 3 key questions. The reference lists of these 101 articles and the 47 review papers identified another 18 articles for full-text review, and 1 additional study that was electronically published following the literature search was identified by the authors. Among these 19 articles, 3 were retained for a final total of 104 studies. Many of the articles excluded at the full-text stage employed advanced MRI techniques in the brain but not the spinal cord, or the number of subjects fell below the threshold. Several articles were also excluded that used MT as a method to enhance contrast between the spinal cord and surrounding tissues, but did not perform quantitative analyses such as computing MTR or MTCSF. Of the final 104 articles, 101 (97%) were identified by the electronic database search.

**Fig. 2:**
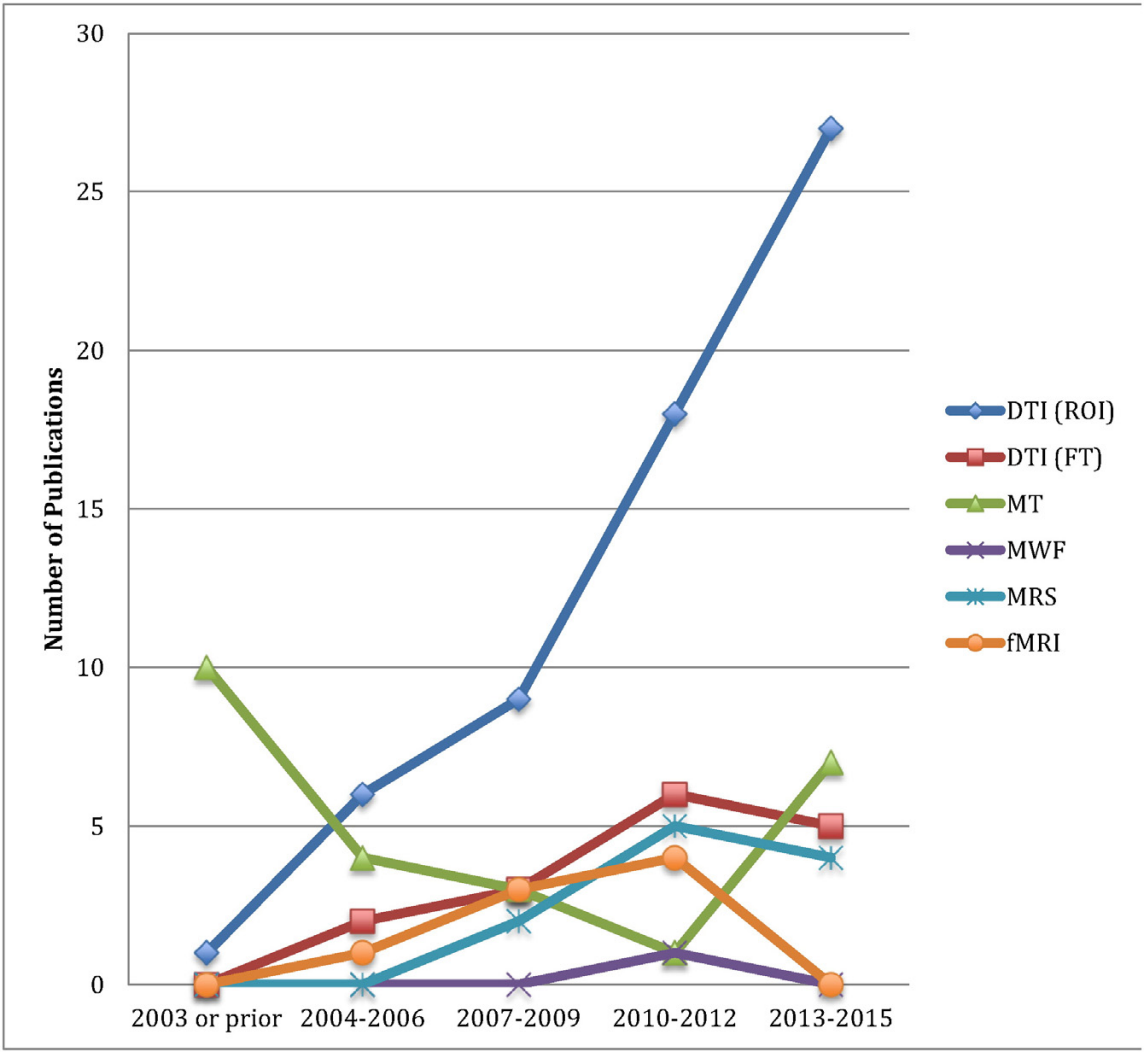
Chronological trends in clinical/translational studies utilizing state-of-the-art spinal cord MRI techniques.

The systematic review identified 69 DTI studies, including 62 that performed ROI-based quantitative analysis and 16 that performed fiber tractography (FT), 25 MT studies, 1 MWF study, 11 MRS studies, and 8 fMRI studies. Ten of the studies employed multi-modal acquisition techniques, including DTI and MT (6 studies), DTI and fMRI (3 studies), or DTI and MRS (1 study). Eight studies that used DTI FT also performed ROI-based quantitative analysis. The chronological trends of each of these imaging techniques are displayed in Fig. 2. The number of DTI studies that used ROI-based analysis sharply increased in recent years, whereas FT analysis decreased slightly. MT studies decreased after 2003, but saw a resurgence in recent years. MRS, MWF, and fMRI have been used in only a small number of studies, and recent use of these techniques has been limited. Table 3, Table 4, Table 5, Table 6, Table 7, Table 8 summarize the details of each study included in the review, separated by the imaging modality that was employed (with DTI divided by analysis technique).

### 3.2 Methodology and risk of bias of individual studies

Among the 104 studies, the risk of bias assessment found moderately low risk (with regards to at least 1 of the key questions) in only 6 studies, with the remainder of studies showing moderately high (24) or high (74) risk. Among the 69 DTI studies, the risk of bias was felt to be high in 52, moderately high in 14, and moderately low in only 3 studies. For MT studies this risk was high in 12, moderately high in 8, and moderately low in 5 studies. MRS studies showed high risk of bias in 7 studies and moderately high risk in 4. All of the fMRI studies and the single MWF study were all assessed to have high risk of bias. Most of the studies reviewed were exploratory in nature (i.e. early translational studies) and not clearly based on a priori hypotheses, frequently making many statistical comparisons without appropriate correction. Most were prospective cohort studies (101), and the remaining 3 were retrospective cohort studies. Furthermore, 43 of the 104 studies failed to account for confounding factors such as age and/or gender, either by ensuring age/gender-matched groups or by performing appropriate multivariate analyses. The vast majority of studies focused on a population with a homogenous diagnosis (98/104), avoiding possible issues with internal validity. However, only 15 of the 104 studies clearly reported the use of consecutive or random enrolment procedures to avoid possible selection bias, whereas the remaining 89 studies either used convenience sampling or failed to report enrolment methods in detail. Most of the studies (82/104) included patients with a range of severity of impairment, including mild/early cases that are more difficult to diagnose.

### 3.3 Acquisition techniques

Among the reviewed studies, a large fraction utilized technical methods that could introduce significant bias in terms of quantitative results. The group of DTI studies used a wide range of pulse sequences, with the majority (41/69) employing a relatively straightforward single-shot EPI (ssEPI) sequence, whereas 3 studies used multi-shot EPI (msEPI), 9 studies used more complex reduced field of view (rFOV) techniques, 1 study used line scan DTI, 1 study utilized a fast spin echo (FSE) sequence, one study used a spectral adiabatic inversion recovery (SPAIR) sequence, and the remaining 13 studies did not provide sequence details. Acquisition parameters were also highly variable, including b-values, FOV, matrix, number of excitations (NEX), saturation bands, shimming, and the use of cardiac gating, which was employed in 16/69 (23%) studies. Two of the studies utilized multiple b-values and calculated measures of diffusion kurtosis, such as mean kurtosis (MK) and root mean square displacement (RMSD) (Hori et al., 2012, Raz et al., 2013). 27 of 69 studies acquired images with very large voxels (greater than 1.5 × 1.5 × 5 mm in at least 1 dimension) or failed to report resolution, potentially biasing the results due to increased partial volume effects. Several studies also performed analyses that could introduce a systematic bias against the pathological group, such as obtaining FA from an ROI in thinned spinal cord tissue at the level of syringomyelia or a hemorrhagic SCI lesion, which is more likely to include voxels with partial volume effects that artificially lower FA (Cheran et al., 2011, Hatem et al., 2009, Hatem et al., 2010, Koskinen et al., 2013, Yan et al., 2015). The group of MT studies tended to use more consistent acquisition methods with less variation, with 24/25 studies employing some form of gradient echo (GE) sequence, all studies using a sinc or Gaussian shaped saturating pre-pulse, and none of the studies utilizing cardiac gating. Only 2 studies computed MTCSF following a single MT acquisition. The remaining 23 studies acquired images with and without a saturation pre-pulse, coregistered the images, and calculated MTR. The study investigating MWF used a 32-echo sequence with inversion recovery (without cardiac gating) to measure the short T2 component using a multi-exponential model, but this technique only acquired a single axial slice with an acquisition time of 30 min. All of the MRS studies uniformly employed similar acquisition sequences, making use of point-resolved spectroscopy (PRESS) with chemical shift selective (CHESS) water suppression, while cardiac gating was employed in 5/11 (45%). Unfortunately, these studies all produced metrics with wide confidence intervals within subject groups. All of the spinal fMRI studies were based on a fast spin echo (FSE) acquisition, and none used cardiac gating. The fMRI studies appeared to suffer from challenges with reliable acquisitions, although reporting was not detailed enough to determine confidence intervals or measures of reliability, as the results typically involved processed data in terms of group activations and connectivity analyses.

### 3.4 Analysis methods

Whole-cord ROIs were used in the vast majority of DTI, MT, and MWF studies. Among the 62 ROI-based DTI studies, 18 reported tract-specific metrics, 3 extracted metrics from WM, and 2 reported data from GM, with the remaining 39 reporting whole-cord metrics or non-specific ROIs (e.g. mixed GM and WM from a mid-sagittal slice). Among DTI FT studies, only 2 reported tract-specific metrics, with the remainder averaging results across all WM identified. 5/25 MT studies reported tract-specific metrics, 1 averaged results across all WM, and 2 offered GM-specific metrics. All MRS results were whole-cord, and fMRI results were typically broken into cord quadrants (combining GM and WM). Only 5 of the ROI-based DTI studies performed automated (or semi-automated) selection of the ROI (Nair et al., 2010, Oh et al., 2013a, Oh et al., 2013b, Oh et al., 2015, Toosy et al., 2014), whereas the other 57 studies introduced potential bias by performing manual ROI selection without blinding procedures. The most common automated method was a simple segmentation procedure, followed by extraction from the whole cord. Nair et al. (2010) used FA values of each subject to create a WM skeleton, and then used this map to draw ROIs from C1 to C6, in a method that is somewhat similar to tractography-based ROI selection. Toosy et al. (2014) performed automated segmentation and registration to a spinal cord template, and subsequently extracted whole-cord ROIs and also hyperintense lesions using an automated threshold-free cluster enhancement (TFCE) algorithm. In addition, 7 studies utilized a semi-automatic algorithm to perform spinal cord segmentation, but then performed manual exclusion of edge voxels that were subject to partial volume effects with contamination from CSF (Agosta et al., 2007a, Agosta et al., 2008b, Agosta et al., 2009a, Agosta et al., 2009b, Benedetti et al., 2010, Manconi et al., 2008, Valsasina et al., 2007), which could introduce bias in the same manner as manual ROI selection. Another study performed random ROI placement to avoid issues of potential bias, but did not report the exact method of randomization (Kamble et al., 2011). Among the 16 DTI FT studies, 6 utilized automatic ROI selection based on the FT output, although 4 of these used manual seed points to initiate the FT algorithm and 1 did not report details on the use of seed points (Hatem et al., 2010). Budzik et al. (2011) performed semi-automated FT without manual seed points and extracted whole-cord ROIs automatically. Among the MT studies, 14 of the 25 studies utilized automatic or semi-automatic analysis methods to extract MTR or MTCSF, with only a minority of studies using manual ROI selection. Rather than exclude edge voxels manually, many of these studies excluded voxels based on a preset threshold of MTR < 10%. The single MWF study used manual ROI selection. The 11 MRS studies were all single-voxel ROIs, with relatively straightforward analysis methods. All of the fMRI studies used a complex series of steps in data analysis, and 7/8 of the reviewed studies made statistical assumptions without correcting for multiple comparisons, leading to potentially biased results. All of the fMRI studies manually divided the cord into quadrants or hemi-cords.

### 3.5 Evidence regarding diagnostic utility

Ninety-five of the 104 studies included in the review made comparisons between pathological subjects and healthy controls. Among these 95 studies, 88 had a high risk of bias, and 7 had a moderately high risk. The vast majority of these studies (89/95) only reported group differences and did not calculate diagnostic accuracy in terms of SE, SP, PPV, or NPV. Group comparisons between pathological subjects and healthy controls frequently showed similarities across different diseases including decreased FA, increased MD, increased RD, decreased MK, decreased MTR, increased MTCSF, and decreased NAA concentration, suggesting various clinical pathologies share common underlying injury mechanisms of demyelination, axonal loss, and GM loss. All 6 of the studies that reported diagnostic accuracy (SE, SP) results utilized DTI, with 4 showing moderate utility of DTI metrics in diagnosing CSM, 1 in CM, and 1 in MS. In CSM, the reported values of SE and SP of DTI metrics ranged from 50 to 100%, but tended to exceed those reported for T2w-HI. However, none of the reported values for diagnostic accuracy were sufficiently high to compete with the gold standard for CSM diagnosis, which is based upon clinical signs of myelopathy along with imaging evidence of any amount of cord compression (typically using conventional MRI). The evidence for diagnostic utility in the CM and MS studies was also not sufficient to consider DTI superior to existing diagnostics. Two studies (both using DTI) computed z-statistics for metrics at each vertebral level to determine if an individual measurement was normal or abnormal. Results pertaining to diagnostic utility are summarized for each clinical pathology in Table 9.

### 3.6 Evidence regarding biomarker utility

A total of 67 studies assessed correlation of MRI metrics with measures of clinical impairment. The risk of bias was high in 40 of these studies, moderately high in 21, and moderately low in 6. Most of these studies (57/67) performed univariate or multivariate correlations, although 10 studies took the simplistic approach of dividing subjects into categories of severity (above/below artibrary thresholds) and then comparing group differences in metrics. Among these studies, the majority (38/67, 57%) only investigated correlations with a single coarse clinical measure, such as Expanded Disability Status Scale (EDSS), Japanese Orthopedic Association (JOA), modified JOA (mJOA), amyotrophic lateral sclerosis (ALS) Functional Rating Scale (ALSFRS), or ASIA Impairment Scale (AIS), rather than employing a battery of assessments or using more detailed measures of impairment such as ASIA motor/sensory scores. The majority of DTI studies reporting biomarker utility results focused on the metric FA, which was particularly successful in CSM with significant results in 5/5 studies correlating with JOA or mJOA (Spearman r = 0.48–0.88, Pearson R = 0.57–0.64) (Ellingson et al., 2014, Gao et al., 2013, Jones et al., 2013, Maki et al., 2015, Wen et al., 2014b) and in SCI in 4/4 studies correlating with ASIA motor/sensory scores (r = 0.59–0.74, R = 0.78–0.92) (Cheran et al., 2011, Cohen-Adad et al., 2011, Koskinen et al., 2013, Vedantam et al., 2015), but slightly less successful in MS with significant results in only 7/15 studies correlating with EDSS (r = − 0.37–0.51, R = − 0.60) (Agosta et al., 2007a, Benedetti et al., 2010, Naismith et al., 2013, Oh et al., 2013a, Oh et al., 2013b, Toosy et al., 2014, Valsasina et al., 2005), with negative results in 8 studies (Agosta et al., 2005, Ciccarelli et al., 2007, Hodel et al., 2013, Miraldi et al., 2013, Oh et al., 2015, Raz et al., 2013, Rocca et al., 2012, Ukmar et al., 2012). Other metrics had limited success in MS correlating with EDSS, with significant results for MD in 3/13 studies (Oh et al., 2013a, Oh et al., 2013b, Valsasina et al., 2005), RD in 4/8 studies (Naismith et al., 2013, Oh et al., 2013a, Oh et al., 2013b, Toosy et al., 2014), MTR in 6/15 studies (Agosta et al., 2007b, Bozzali et al., 1999, Kearney et al., 2014, Lycklama et al., 2000, Oh et al., 2013a, Oh et al., 2013b), MTCSF in 2/2 studies (Fatemi et al., 2005, Zackowski et al., 2009), and the number of active voxels using fMRI in 1/3 studies (Valsasina et al., 2010) whereas no correlation was found between EDSS and the DKI metric MK (1 study) (Raz et al., 2013) and the MRS metric NAA (or relative NAA concentration) in 5/5 studies. Three studies used longitudinal imaging and clinical data collection to assess if changes in MRI metrics over time reflected changes in clinical status, but the results were negative in 2/2 studies using DTI in ALS and 1 study using MWF in MS. Results for biomarker utility, divided by clinical pathology, are summarized in Table 9.

### 3.7 Evidence regarding predictive utility

Longitudinal studies that assessed predictive utility of advanced MRI metrics were only conducted in a total of 10 studies involving MS (5), ALS (2), CSM (2), and CM (1). Among these, 6 utilized DTI, 3 used MRS, 1 used MT, and 1 used MWF. The risk of bias among these studies was assessed as high in 8 and moderately high in 2. Four additional studies collected longitudinal clinical data but did not report prediction of outcomes using baseline MRI metrics. Among the 10 studies investigating predictive utility, 5 employed a detailed battery of clinical assessments (Bellenberg et al., 2013, El Mendili et al., 2014, Freund et al., 2010, Ikeda et al., 2013, Jones et al., 2013). Baseline FA showed weak to moderate correlations with clinical outcomes such as ALSFRS in ALS (1 study), mJOA recovery ratio in CSM (1/2 studies), and EDSS in MS (2/2 studies), but not mJOA in CSM (1 study). Ratios involving NAA were predictive of outcome in ALS (1 study) and MS (1/2 studies). Results for predictive utility are summarized in Table 9.

### 3.8 Evidence summary

The vast majority of studies included in this review had high or moderately high risk of bias, leading to a low baseline quality of evidence for each of the specific findings listed in Table 10. For the specific finding that FA is decreased in terms of group differences between patients and healthy controls in ALS, CSM, myelitis, MS, neuromyelitis optica (NMO), and SCI, the overall quality of evidence was neither upgraded nor downgraded, and remained low. Other metrics MD, RD, MK, MTR, MTCSF, and NAA also showed group differences between patients and healthy subjects in various clinical conditions, but the quality of evidence for these metrics was downgraded to very low due to a low level of evidence (MK, MTCSF) or inconsistent results between studies (MD, RD, MTR, NAA). There was insufficient evidence available to make any recommendations regarding the diagnostic utility (in terms of detecting group differences) of AD, standard deviation of primary eigenvector orientation (SD(θ)), orientation entropy (OE), tractography pattern, MWF, and fMRI-based metrics due to a lack of evidence, inconsistent results, and wide confidence intervals in many of the studies. The overall quality of evidence for diagnostic accuracy (sensitivity and specificity) was also insufficient, which was downgraded 2 levels due to highly inconsistent results. In terms of biomarker utility, only FA demonstrated consistent results, and the quality of evidence was upgraded 1 level to moderate for showing a dose–response gradient. The evidence for other MRI metrics as biomarkers was inconsistent and imprecise, leading to a finding of insufficient evidence. Finally, the evidence regarding the predictive utility for all MRI metrics was inconsistent and imprecise, leading to a rating of insufficient.

## 4 Discussion

It is an exciting time in spinal cord imaging, as the emergence of powerful new MRI techniques has inspired a large number of early clinical studies of pathological spine conditions. The excellent research conducted to date has demonstrated tremendous potential for all of these techniques to elucidate aspects of the microstructure or function within the human spinal cord, adding numerous insights into the pathophysiology of several neurological diseases. Among the 5 new techniques addressed in this review, DTI has thus far generated the most research, comprising 66% of the included studies and showing a sharp increase within the past 6 years, particularly using ROI-based analysis (Fig. 2). This increase in interest is most likely related to the promising results that DTI studies have demonstrated, particularly with moderate evidence that FA is a biomarker for disability in numerous pathologies (Table 10). The correlation of FA with impairment appears to be strongest in diseases that are confined to the spinal cord (e.g. CSM), which is consistent with the concept that disability in more distributed diseases (e.g. MS) is caused by injury to both the brain and the spinal cord. Low evidence was also found suggesting that FA shows group differences compared with healthy controls in several conditions, but insufficient evidence was available to suggest that DTI provides improved diagnostic accuracy or prediction of outcomes over established methods. A very low level of evidence was found for group differences using other DTI metrics MD and RD, MT metrics MTR and MTCSF, and the MRS metric of NAA concentration. It is unclear based on the current body of evidence if these metrics have substantial diagnostic value, due to a lack of strong evidence and substantial inconsistencies in results to date. The lack of well-designed studies to determine the diagnostic utility of the advanced MRI techniques, with 93% having a high risk of bias and only 6% reporting sensitivity and specificity, suggests a profound knowledge gap for future research. Furthermore, several studies in the review suggested that the simple quantitative measure of spinal cord CSA (quantifying atrophy) outperforms all of the advanced MRI metrics in terms of diagnostic and biomarker utility (Kearney et al., 2014, Kearney et al., 2015, Oh et al., 2013b, Oh et al., 2015), suggesting that stronger results are still needed to contemplate the clinical uptake of these techniques.

### 4.1 Interpreting the evidence in the context of risk of bias

Unfortunately, the vast majority of studies (98/104, 94%) completed to date have a high or moderately high risk of bias, indicating the relative immaturity of the research in the field thus far. Although we were unable to determine precisely how many of the studies were based on a priori hypotheses (often due to ambiguous reporting of methods), it was obvious that most studies were highly exploratory, as they frequently analyzed numerous metrics and ROIs/levels without statistical correction to avoid type I errors. The early nature of the body of evidence is also apparent in the fact that 86% of studies failed to explicitly use randon/consecutive enrolment methods, and 41% did not perform age/gender matching in group comparisons or analysis for these potential confounders when assessing correlations or prediction of outcomes. Comparing the risk of bias between the 5 advanced MRI techniques, it was found to be lowest in MT studies, rated as moderately low in 20%, moderately high in 32%, and high in 48%, primarily as a result of more reliable, consistent acquisition methods and a tendency to more frequently utilize automated analysis techniques. However, in spite of these advantages, the results of the MT studies (most commonly using the metric MTR) showed considerably less consistent results compared with the DTI metric FA in terms of detecting group differences and correlating with impairment. As a result, the overall quality of evidence for MTR (and MTCSF) to demonstrate group differences in various clinical conditions was considered very low, and the evidence for their utility as biomarkers was insufficient (Table 10). This is suggestive that MTR is, overall, a weaker marker of pathological changes in the diseases studied than FA, although these metrics appear to measure separate components of microstructural change (Cohen-Adad et al., 2011, Wheeler-Kingshot et al., 2002), and the differences in consistency of results could alternatively be explained by technical factors. The risk of bias among DTI studies was assessed as high in 75% and moderately high in another 20%, largely as a result of problems with acquisition methods such as very large voxels (39%) and a lack of automated/objective analyses (86%). The lack of a substantial number of high quality DTI studies led to a low baseline level of evidence for FA, MD, RD, and MK to demonstrate group differences and utility as a biomarker (Table 10). The quality of evidence for FA as a biomarker was upgraded to moderate due to a “dose–response gradient” (a term used in GRADE) as it shows consistent and relatively strong correlations with impairment, whereas the evidence for MD, RD, and MK were downgraded to very low in terms of diagnostic utility (showing group differences) and insufficient in terms of value as biomarkers. The risk of bias in MRS studies was high in 64% and moderately high in the remaining 36%, related to technical problems with acquisitions that resulted in the exclusion of subjects and wide confidence intervals in reported metrics. NAA showed very promising results in some studies, but the overall evidence was again downgraded to very low in terms of group differences and insufficient for correlation with impairment due to inconsistent results and imprecise estimates of effect. The single MWF study and all of the spinal fMRI studies were deemed to have a high risk of bias, primarily relating to difficulties in acquiring reliable images and the use of liberal statistical assumptions. As a result, none of the metrics investigated in these studies were deemed to have thus far demonstrated utility in terms of the three key questions.

### 4.2 The design of imaging studies for clinical translation

The incorporation of detailed clinical assessments into translational study protocols provides a richer and more objective characterization of patients' functional impairments compared with coarse clinical tools such as EDSS, JOA, mJOA, ALSFRS, and AIS. The majority of studies that investigated biomarker utility (57%) and half of the prognostic studies employed only a single coarse measure of impairment. The use of these summary measures of disability risks misrepresenting the degree to which the spinal cord and specific WM tracts are truly injured, as these measures are imprecise, and results can be strongly influenced by counfounding factors, such as reporting bias (in self-reported measures) or brain involvement in distributed CNS diseases (e.g. MS). If considerable noise and inaccuracies are present in the clinical assessments, the process of trying to identify meaningful correlations with MRI metrics can become futile. The additional use of electrophysiology (EP) tests can be used to augment the clinical information, although it is important that these test do not replace detailed neurological/functional assessments, as in some cases they may not be sufficiently sensitive or specific (Kerkovsky et al., 2012). However, it should be noted that a trend appears to be emerging, with many recent studies employing a broader array of clinical tests. Future studies that generate fine-grained clinical data using a battery of assessments are more likely to identify important correlations with disability, and such high fidelity data may even have the power to show strong relationships between MRI changes in individual WM tracts and focal neurological deficits that uniquely occur in each specific disease.

### 4.3 State-of-the-art spinal cord MRI acquisition techniques: a work in progress

“*The only thing that is constant is change*.” — Heraclitus, 500 BC. Although many technological advances have been made, the state-of-the-art spinal cord MRI techniques addressed in this review remain a work in progress, with many technical hurdles remaining. All of these imaging techniques are much more difficult to implement in the spinal cord than other regions, such as the brain, which has attracted many talented MRI physicists and engineers to take on this challenge. The issues of magnetic field inhomogeneity and physiological motion, leading to various artifacts and image distortions, remain significant barriers to high quality data collection for all of the techniques. DTI, most commonly based on spin echo EPI sequences, is an inherently noisy technique that typically requires large voxels and/or the use of multiple excitations to achieve acceptable SNR, both of which can increase partial volume effects at the cord periphery. The substantial variability in acquisition methods used by spinal cord DTI research groups indicates that this community is far from reaching consensus on the optimal approach to this difficult problem. The most common DTI sequence employed was ssEPI (59%), which tends to allow short acquisition times (< 5 min in the majority of reviewed studies; Table 3, Table 4). 11/69 studies took advantage of these short scan times and used the approach of performing multiple ssEPI acquisitions and averaging the results offline to improve SNR, using coregistration and motion correction tools. However, it should be noted that EPI involves important tradeoffs, as it is strongly affected by susceptibility artifact due to inhomogeneity in the magnetic field. This effect can cause image distortions, particularly at the level of intervertebral disk spaces, which is exaggerated when herniated disks obliterate the anterior CSF, potentially introducing bias or invalidating metrics calculated in the compressed portion of the spinal cord in conditions such as CSM. For example, Kerkovsky et al. (2012) report decreased FA in patients with spinal cord encroachment (effacement on the CSF) that have neck pain or radiculopathy but no objective signs of myelopathy. This result could represent sub-clinical changes in the spinal cord microstructure, but could alternatively be explained by increased susceptibility artifact. In recent years, there has been increased use of rFOV techniques, although this approach was only utilized in 13% of the reviewed studies. These sequences are based on 2D radiofrequency (RF) excitation (Finsterbusch, 2009, Saritas et al., 2008) or oblique refocusing pulses (Dowell et al., 2009, Wilm et al., 2009), and allow the use of a smaller FOV with higher resolution while avoiding aliasing problems and decreasing distortions, albeit at a cost of increased acquisition time. Only a fraction of DTI studies (23%) employed cardiac gating, likely because most groups felt that the reduction in motion artifacts is not worth the increased acquisition time and added complexity of setting up cardiac monitoring equipment. Two diffusion studies collected data with multiple b-values and computed measures of diffusion kurtosis, which is a dimensionless measure of the deviation from a Gaussian probability curve, with a positive value reflecting a sharper peak and heavier tails (Hori et al., 2012, Raz et al., 2013). Both studies identified positive MK in all subjects, with pathological subjects in CSM (Hori et al., 2012) and MS (Raz et al., 2013) showing group decreases in MK. However, it is unclear if DKI measures are sufficiently more powerful than simple DTI metrics to justify the added acquisition time required for multiple b-values. However, the optimal number of diffusion-sensitizing directions has not been established for DKI, but it may be possible that DKI can be performed with a smaller number of directions, possibly offsetting the need for multiple b-values. As mentioned above, all of the MT studies utilized similar acquisition methods such as GE sequences (except for the earliest study (Silver et al., 1997)), MT pre-pulse parameters, and resolution. The single WMF study was exploratory in nature, and further refinements in spinal cord MWF image acquisition, including decreased scan time, are needed prior to the initiation of more advanced clinical studies using this method. MRS, particularly of the spinal cord, is prone to motion artifact and low SNR, typically requiring relatively long acquisition times due to the use of complex shimming methods, a high number of signal averages, and cardiac gating to obtain useful data. The magnetic field inhomogeneity within the spinal canal makes it difficult to shim the B0 field, usually requiring high-order shimming procedures to attempt to compensate. As a result, there is line broadening in the metabolite peaks and decreases amplitude, making detection difficult. MRS studies had the highest use of cardiac gating at 45% compared to other techniques in this review. The MRS results demonstrate significant variations in metabolite concentrations and ratios, even among healthy individuals (Holly et al., 2009, Ikeda et al., 2013, Salamon et al., 2013), suggesting that noise may still be a major limitation. However, it may also be the case that there naturally exists a wide range of normal in the concentrations and ratios of the molecules that MRS captures, in which case it will be difficult for MRS to make strong assertions about individual patients, even with further technical improvements. However, MRS provides unique information compared with the other advanced MRI techniques, and further development may allow quantification of important CNS molecules such as glutamate (not reliably detected with current methods), which may suggest an important role for MRS to compliment the other more anatomically specific techniques. All 8 of the spinal fMRI studies used a fast or turbo SE pulse sequence with SEEP contrast, compared with T2*-weighted EPI that is typically used in brain fMRI based on BOLD contrast. FSE is commonly employed in spinal fMRI to compensate for severe inhomogeneity of the magnetic field within the spinal canal, but the readouts from this technique are considerably slower than EPI, increasing the effects of physiological motion artifacts. The time to acquire each volume of images in the reviewed studies ranged from 8 to 13 s, collecting between 5 and 9 slices (axial orientation in 7 studies, sagittal in 1) per volume, indicating the relatively low temporal resolution compared with brain fMRI, in which an entire brain volume can be acquired in 2 to 4 s. Furthermore, the signal change relating to altered neural activity is frequently only 2–3% (Stroman et al., 2004), requiring high SNR to reliably differentiate active voxels from background noise. The overall results of the spinal fMRI studies did not show convincing changes in activation patterns in specific pathologies (only minor loss of ipsilateral focal activation), possibly due to technical problems achieving sufficient SNR. If, however, reliable activations can be detected with better temporal resolution and shorter acquisition time, fMRI will likely make a significant impact, with obvious applications in conditions such as SCI to detect new activity and connectivity as regeneration therapies (e.g. stem cells) are studied. In summary, all 5 of the state-of-the-art spinal cord MRI techniques continue to face technical issues that require further innovations, and clinical studies face the limitation of needing to freeze on a specific acquisition methodology over the period of time required to complete data collection, even if it may not include the latest and greatest technical advances.

### 4.4 State-of-the-art imaging deserves state-of-the-art analysis

The majority of DTI, MT, MWF, and fMRI studies included in this review used manual methods of ROI selection to extract quantitative metrics, with only 25/93 (27%) using automated or semi-automated ROI selection. In addition to being slow and imprecise, unblinded manual ROI selection is an obvious source of potential bias in studies, as the technician selecting the ROI can arbitrarily include or omit pixels of high or low signal (often present at the edge of the cord due to partial volume effects), and it is impossible to blind the technician in many scenarios (e.g. compressive myelopathy). The very low rate of objective analysis techniques for DTI studies (14%), compared with 56% of MT studies, is possibly due to greater problems with partial volume effects at the edge of the cord in DTI, where contamination with CSF causes an increase in isotropic diffusion and a corresponding decrease in FA, prompting 7 DTI studies to employ manual exclusion of edge voxels after performing semi-automated segmentation to identify the spinal cord. Furthermore, most studies (73/104, 70%) included in this review reported whole-cord metrics, which average the effects of a specific disease process across all GM and WM. Analyzing whole-cord metrics lacks the specificity of measuring changes in individual anatomical areas, such as WM tracts (which might be differentially affected in a certain disease), and it also potentially dilutes the sensitivity to detect small changes: a 10% change present in the WM might only show a 5% change in the whole-cord metric, which may no longer be statistically significant. To optimize the sensitivity and specificity of these techniques, the ideal solution is to analyze only the tissue that is most affected by a certain disease, such as the anterior horn GM and/or the lateral corticospinal tracts in ALS. Several groups are actively developing tools for this purpose, which can perform a series of complex data processing steps and automatically extract quantitative metrics from GM, WM, and specific WM-tracts (Cohen-Adad et al., 2014), even correcting for partial volume effects at the cord periphery (Levy et al., 2015). Tract-specific metrics, which were available in only 22/104 studies (21%), also have the advantage of potentially characterizing gradations of injury to each anatomical area within the cord, potentially correlating with or predicting focal neurological deficits. Fiber tractography (FT) is an interesting alternative to ROI-based quantitative analyses of DTI data. The DTI studies that employed FT were listed separately from ROI studies in Table 4, primarily to identify trends and commonalities among the methods used within FT studies. Among the FT studies reviewed, only 38% extracted quantitative metrics from the region defined by the FT results. The utility of FT in quantitative assessment of the spinal cord is controversial, as some have suggested that using FT to automatically define ROIs is inherently biased (Cohen-Adad et al., 2011), and most FT algorithms require manual seed points, as was identified in our review (only 1/16 studies did not require seed points). However, one study in this review reported improved measures of inter-observer reliability using FT-based ROIs vs. manual ROIs, again supporting the importance of automated, objective analysis methods (Van Hecke et al., 2009). Other studies derived quantitative measures from the FT output, such as number of fibers, fiber density, or fiber length (as surrogates for number of intact axons). However, the FT analysis is typically based on liberal assumptions of what constitutes a fiber, using low thresholds for minimum FA of 0.10–0.30 and angle of < 20–70° when calculating connections between voxels. The result is a very loose representation of the actual white matter that should be interpreted with caution. An alternative to using tractography to measure the organization of the white matter is to perform quantitative analysis of the directionality of the eigenvectors, which was performed in 2 studies using OE and SD(θ). These alternative methods are highly quantitative, and may turn out to be more reliable than tractography in characterizing white matter changes, but greater data is needed to fully define their value. Half of the FT studies, all of which involved various forms of compressive myelopathy, only reported descriptions of the pattern of tracked fibers such as the degree of deformation or disruption. However, assignment of these descriptors is highly subjective and WM compression may be more accurately represented by geometric measurements (e.g. maximum spinal cord compression ratio). In comparing MT techniques, the use of MTR may have a theoretical advantage over MTCSF, as the CSF is prone to flow artifact that causes signal dropout, which could potentially bias results, but this was not an obvious drawback in the 2 studies that employed MTCSF. The calculation of MTR requires an added post-processing step, as images with and without an MT prepulse need to be co-registered accurately, but this is relatively straightforward with modern tools. No major technical challenges were identified in the analysis techniques employed by MWF and MRS studies, except for the use of manual ROIs in the WMF study (Laule et al., 2010). In all of the reviewed fMRI studies, time-series data were analyzed by convolving with a canonical hemodynamic response function, and activation maps (based on a p-value threshold or a clustering algorithm) were created. Due to challenges in obtaining robust activations, most of the spinal fMRI studies used an uncorrected threshold of P < 0.05 for each voxel so that a greater number of activations could be identified, with the exception of one study (Cadotte et al., 2012). This uncorrected analysis runs a high risk of identifying false activations, particularly when hundreds of voxels are included, and therefore the results of these studies must be interpreted with caution. All of the fMRI studies also used manual ROI selection, typically dividing the cord into quadrants manually, contributing another potential source of bias to the analysis.

### 4.5 Statistical analysis: a big data problem

Appropriate statistical analysis for complex clinical studies using quantitative MRI techniques is far from straightforward. This data can involve a large number of metrics, including multiple DTI indices or the output from multi-modal acquisitions, and the values might be extracted from numerous ROIs located in individual WM tracts at many rostro-caudal levels of the spinal cord. Furthermore, the above-mentioned trend toward using multiple clinical measures to fully characterize disability suggests that future studies will need to employ multivariate analyses with an increasing number of independent and dependent variables. The analysis of these studies quickly becomes a big data problem, and help from an experienced statistician is advisable to correctly design robust multivariate analyses that incorporate a priori variables of interest and potential confounding factors such as age and gender. It is of paramount importance that a priori hypotheses are clearly stated beforehand, to avoid an excess number of comparisons and misrepresentation of the complex data to make unfounded conclusions. Among the studies reviewed, there were many cases where no correction was made for multiple comparisons, leading to findings that would not have been identified as significant with proper correction. In some cases, studies went as far as reporting conclusions that were clearly overstated or unfounded, which must be avoided in future translational research that will form the basis for clinical adoption of these techniques.

### 4.6 Limitations of this study

This systematic review attempted to perform an exhaustive review of all clinical studies utilizing the 5 advanced spinal cord MRI techniques. A large number of citations were analyzed in an attempt to identify all relevant articles, but it is still possible that relevant studies were missed, including those not available in English. On the other hand, the large scope of this review made it more difficult to discuss all of the subtleties involved in these MRI techniques. Also, the inclusion criteria arbitrarily excluded cohorts with fewer than 24 subjects or fewer than 12 pathological subjects. This threshold was originally set at 20 total subjects and 10 pathological subjects, but it was increased because the number of studies identified using the lower threshold was far greater than 100, which would have made the tables excessively long and the discussion even more difficult. However, we did not increase the threshold higher than 24 as we felt that several key studies would have been excluded. Studies that only analyzed the quantitative metrics apparent diffusion coefficient (ADC), generated from DWI, or CSA, derived from anatomical images, were also excluded for the purpose of focusing this review on new techniques. Spinal cord DWI has been in clinical use for many years for the detection of infarction and abscess, but the simple metric of ADC (equivalent to MD in DTI) may have value in specific applications as a measure of microstructural tissue changes. CSA is clearly a powerful quantitative metric that relates to cord atrophy, which should be considered for use in addition to the advanced MRI metrics in multivariate models. The search strategy excluded research that only studied healthy subjects, as these studies and those with smaller cohorts of pathological subjects tended to show less robust methodology and clinical relevance. This review also focused solely on advanced spinal cord imaging techniques, but several groups studying spinal cord pathologies have investigated imaging changes in brain microstructure and function, in part due to the relative simplicity of implementing these imaging protocols in the brain (Freund et al., 2013, Kowalczyk et al., 2012, Mikulis et al., 2002). Furthermore, this review was focused on the 5 most promising spinal cord imaging techniques identified by the recent expert panel, but several others are emerging that may make a substantial impact to this field, including perfusion imaging, susceptibility weighted imaging, T1 relaxometry, neurite orientation dispersion and density imaging (NODDI), and myelin g-ratio (Stikov et al., 2015).

### 4.7 Future directions

The path to clinical translation of technological innovations, such as new MRI techniques, invariably includes numerous challenges and there remains significant work to successfully bring these techniques into clinical use. Translational research typically involves a process that begins with small exploratory studies and transitions to large, carefully designed clinical trials, and several of the state-of-the-art spinal cord MRI techniques reviewed in this paper have demonstrated sufficiently strong results and are ready for this next step. Looking forward, the spinal cord imaging community will continue to drive these powerful techniques forward, with several key steps happening concurrently: 1) larger clinical studies with specific hypothesis-driven research questions will be designed and conducted to assess for clinical utility; 2) acquisition techniques will continue to evolve and be refined to maximize signal-to-noise ratio (SNR) and resolution while minimizing distortions, artifacts, and acquisition times; and 3) powerful data analysis tools will be developed that can automatically extract quantitative data from the GM, WM, and specific WM tracts. The long path to clinical translation is not easy, but in the coming years, we can expect many further innovations in this burgeoning field, which will hopefully lead to major improvements in the diagnosis and management of patients with spinal cord pathologies.

New techniques and innovations are also emerging that could dramatically alter the course of research in this field, but were not utilized by any of the studies in this review. For example, the development of high strength gradients for DTI, highlighted by the human connectome project that uses 300 mT/m gradients (200 mT/m/ms slew rate) — 8 times stronger than most clinical hardware, have provided new insights, such as mapping the axon diameter distribution in the human spinal cord (Duval et al., 2015). Recently, the introduction of inhomogenously broadened MT (ihMT) imaging has demonstrated much higher specificity for myelin imaging than previous MT techniques (although the signal dropout is less pronounced requiring subtraction between images, which decreases SNR substantially), which will likely spur new clinical studies to investigate its utility (Girard et al., 2015). Chemical Exchange Saturation Transfer (CEST) effect is a particular case of MT imaging, which can quantify the biochemical composition of tissues based on labile protons (hydroxyl, amide, amine, and sulfhydryl moieties). Feasibility in the human spinal cord and application in MS patients have recently been demonstrated (Kim and Cercignani, 2014). In addition, none of the 104 studies that were reviewed used 7 T field strength, but with the proliferation of 7 T research systems and the recent announcement of 7 T clinical scanners, it is inevitable that new clinical studies at ultra-high field strength are coming soon and these could potentially show substantial improvements that strengthen the case for clinical utility. Analysis techniques may also undergo a revolution with the introduction of machine learning, as complex multivariate data from healthy and pathological subjects could be used to train classifiers, potentially increasing diagnostic sensitivity and specificity.

However, optimism for novel MRI methods must be tempered with practicality. Even if the clinical utility of one or more of these quantitative MRI techniques is clearly demonstrated, a considerable hurdle will still remain before widespread clinical adoption will occur. The concept of quantitative MRI has been used in the research domain for several years (e.g. CSA for MS), but is largely foreign to clinicians, and the exact method and workflow for its use needs to be carefully considered, or these new technique will be quickly abandoned. Radiologists, neurologists, and spine surgeons that have busy clinical practices are unlikely to sit at an imaging workstation and perform manual tasks to generate quantitative metrics, so data analysis will need to be fully automated, robust, and seamlessly integrated. The perception that new analysis methods are time consuming, unreliable, or inaccurate will render these new methods unacceptable. Thus it is essential that sophisticated, automatic analysis tools be developed in parallel with advances in the imaging techniques themselves.

## 5 Conclusions

**Fig. 3:**
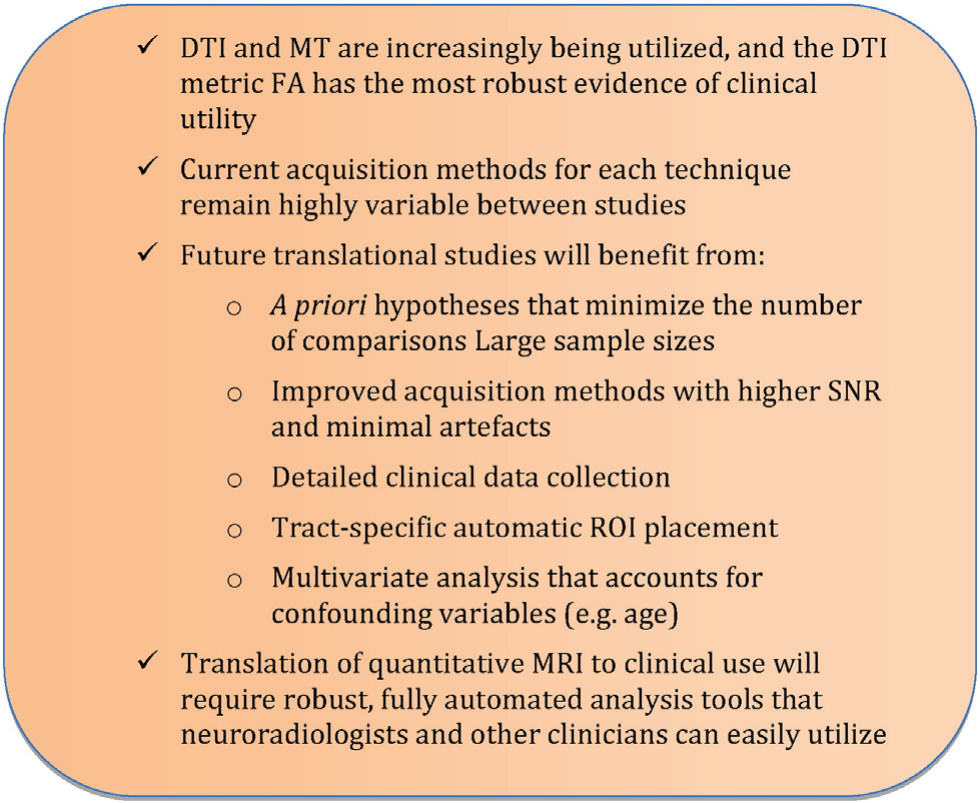
Key points.

The current body of evidence of clinical studies using spinal cord DTI, MT, MWF, MRS, and fMRI is relatively limited, indicating the early stage of this translational research effort. However, moderate evidence indicates that the quantitative DTI metric FA successfully correlates with impairment in a number of neurological disorders. Low evidences suggests that FA shows tissue injury (in terms of group differences) in a number of disorders, but the evidence is insufficient to support its use as a diagnostic test or as a predictor of clinical outcomes. Very low evidence exists for other metrics to show pathological changes in terms of group differences in the spinal cord, including MD, RD, MK, MTR, MTCSF, and NAA, and the evidence is insufficient to determine if they can be used as a diagnostic test, biomarker, or prognostic marker in a clinical context. DTI has produced the most substantial results to date, but acquisition methods, data processing, and interpretation require further refinement, followed by standardization and cross-vendor validation, before this technology is ready for widespread clinical adoption. The path to clinical translation of these complex MRI techniques is not straightforward, and future translational studies are required that have clear a priori hypotheses, large enrolment numbers, short scan times, high quality acquisition techniques, detailed clinical assessments, automated analysis techniques, and robust multivariate statistical analyses (Fig. 3). It is also important to keep in mind that the definition of clinical utility is to be able to make assertions about individual patients, not just achieve significant group differences, setting a very high standard for success. However, much progress has already been made, and the spinal cord imaging community will undoubtedly make many great achievements in the years to come.

## Abbreviations

AC: anterior column
AD: axial diffusivity
ADC: apparent diffusion coefficient
AIS: ASIA impairment scale
ALS: amyotrophic lateral sclerosis
ALSFRS: ALS functional rating scale
ALSFRS-R: revised version of ALSFRS
aSCI: acute SCI
ASIA: American Spinal Injury Association
BMS: benign MS
BOLD: blood oxygen level-dependent
CADASIL: cerebral autosomal dominant arteriopathy with subcortical infacts and leukoencephalopathy
CHESS: chemical selective saturation
Cho: choline (concentration)
Cre: creatinine (concentration)
CM: compressive myelopathy
CO-ZOOM: contiguous slice zonally oblique multislice
CSA: cross-sectional area
cSCI: chronic spinal cord injury
CSM: cervical spondylotic myelopathy
CTL: cervical, thoracic, lumbar
DC: dorsal column
DK: diffusion kurtosis
DTI: diffusion tensor imaging
EDSS: expanded disability status scale
EPI: echo planar imaging
FA: fractional anisotropy; flip angle
FD: fiber density
FDi: fiber density index
FIM: functional independence measure
fMRI: functional MRI
FOV: field of view
FSE: fast spin echo
FVC: forced vital capacity
FT: fiber tractography
FU: follow-up
GE: gradient echo; General Electric
GM: gray matter
GRAPPA: generalized autocalibrating partial parallel acquisition
HASTE: half-Fourier acquisition single-shot turbo spin-echo
HC: healthy control
ICC: intraclass correlation coefficient
ISRT: International Spinal Research Trust
JOA: Japanese Orthopedic Association scale
Lac: lactate (concentration)
LC: lateral column
LCST: lateral corticospinal tract
LHON: Leber's hereditary optic neuropathy
λ_1_: primary eigenvector
λ_2_: secondary eigenvector
λ_3_: tertiary eigenvector
MCL: maximally compressed level
MD: mean diffusivity
MEP: motor evoked potential
mJOA: modified JOA scale
MK: mean kurtosis
mNorris: modified Norris scale
MRI: magnetic resonance imaging
MRS: magnetic resonance spectroscopy
MS: multiple sclerosis
MSFC: MS functional composite scale
MSWS: MS walking scale
MT: magnetization transfer
MTCSF: MT ratio between tissue and CSF
MTR: MT ratio with and without pre-pulse
MWF: myelin water fraction
Myo: myo-inositol
NAA: n-acetylaspartate
NAGM: normal appearing GM
NASC: normal appearing spinal cord
NAWM: normal appearing WM
NEX: number of excitations
NMO: neuromyelitis optica
NPV: negative predictive value
NR: not reported
NS: non-significant
NSA: number of signal averages
OCT: optical coherence tomography
OE: orientation entropy
PAC: phased array coil
PASAT: paced auditory serial addition test
prospective, cross-sectional: prospective, cross-sectional
PD: proton density
PL: prospective, longitudinal
PRESS: point-resolved spectroscopy
PPMS: primary progressive MS
PPV: positive predictive value
RC: retrospective, cross-sectional
RD: radial diffusivity
ResNAA: residual n-acetyl aspartate concentration
rFOV: reduced field of view
RL: retrospective, longitudinal
ROI: region of interest
RLS: restless leg syndrome
RMSD: root mean squared displacement
RRMS: relapsing–remitting MS
Ψ: difference of AD–MD
SCI: spinal cord injury
SD: standard deviation
SD(θ): standard deviation of the angle between primary eigenvectors
SE: spin echo; sensitivity
SEEP: signal enhancement by extravascular protons
SENSE: sensitivity enconding parallel acquisition
SP: specificity
SPGR: spoiled gradient echo
SPMS: secondary progressive MS
SSEP: somatosensory evoked potential
STIR: short-tau inversion recovery
STT: spino-thalamic tract
ssEPI: single shot EPI
T1w: T1-weighted image
T2w: T2-weighted image
T2w-HI: T2w hyper-intensity
TMS: trans-cranial magnetic stimulation
TWT: timed walk test
WfL: Wings for Life
WM: white matter
wOE: weighted orientation entropy

## Figures and Tables

**Table:** 

Ovid MEDLINE (R) 1946 to May week 4 2015			
#	Searches	Results	Search type
1	Diffusion Tensor Imaging/[New MeSH as of 2010]	4098	Advanced
2	Diffusion Magnetic Resonance Imaging/	11,877	Advanced
3	(Diffusion adj1 Tensor? adj1 Imag*).mp.	7767	Advanced
4	(diffusion adj1 weight* adj1 imag*).mp.	5821	Advanced
5	(diffusion adj2 MRI?).mp.	3832	Advanced
6	(diffusion adj2 MR?).mp.	5861	Advanced
7	(diffusion adj2 tractogra*).mp.	719	Advanced
8	(diffusion adj1 coefficien*).mp.	12,916	Advanced
9	(diffusion adj1 co-efficien*).mp.	25	Advanced
10	(dti adj2 tractogra*).mp.	299	Advanced
11	dti.tw,kw.	4963	Advanced
12	(high* adj1 angular* adj1 resolut* adj1 diffusion*).mp.	228	Advanced
13	hardi.tw,kw.	187	Advanced
14	(magneti#at* adj2 transfer*).mp.	2232	Advanced
15	exp magnetic resonance spectroscopy/or electron spin resonance spectroscopy/or nuclear magnetic resonance, biomolecular/	183,850	Advanced
16	(magnetic adj1 resonan* adj1 spectroscop*).mp.	138,238	Advanced
17	(mr adj spectroscop*).mp.	3945	Advanced
18	(mri adj spectroscop*).mp.	55	Advanced
19	(functional adj2 (mri or mris or magnetic resonan*)).mp.	23,954	Advanced
20	fmri?.mp.	23,159	Advanced
21	myelin water fraction*.mp.	80	Advanced
22	1H-MRS.mp.	1246	Advanced
23	ADC.tw,kw.	6071	Advanced
24	ADT.tw,kw.	1634	Advanced
25	(apparent adj1 diffus* adj1 coefficien*).mp.	5429	Advanced
26	(apparent adj1 diffus* adj1 tensor*).mp.	27	Advanced
27	(DTI or DTIs).mp.	5049	Advanced
28	(fraction* adj1 anisotrop*).mp.	4331	Advanced
29	(mean adj1 diffusivit*).mp.	1462	Advanced
30	mrs.tw,kw.	11,830	Advanced
31	mt ratio?.mp.	203	Advanced
32	mtcsf.mp.	2	Advanced
33	MTR.mp.	1455	Advanced
34	MWF.mp.	330	Advanced
35	MWFI.mp.	0	Advanced
36	(NMR adj1 spectroscop*).mp.	30,893	Advanced
37	exp Magnetic Resonance Spectroscopy/	183,850	Advanced
38	(saturat* adj1 spectroscop*).mp.	16	Advanced
39	press.tw,kw.	16,439	Advanced
40	(proton* adj1 MRS).mp.	522	Advanced
41	tractograph*.mp.	2342	Advanced
42	or/1–41 [Advanced MRI & related terms]	280,204	Advanced
43	exp Spine/	112,919	Advanced
44	exp Spinal Diseases/	96,616	Advanced
45	exp Spinal Cord/	81,714	Advanced
46	exp Spinal Cord Diseases/	105,248	Advanced
47	exp spinal cord injuries/	38,997	Advanced
48	exp spinal cord/and exp wounds/	7428	Advanced
49	exp spinal cord/and in.fs.	1446	Advanced
50	exp paraplegia/or brown-sequard syndrome/	11,667	Advanced
51	exp quadriplegia/	7169	Advanced
52	((spine or spinal) adj3 injur*).mp.	44,641	Advanced
53	((spine or spinal) adj3 compress*).mp.	12,835	Advanced
54	((spine or spinal) adj3 contus*).mp.	976	Advanced
55	((spine or spinal) adj3 damag*).mp.	1755	Advanced
56	((spine or spinal) adj3 disrupt*).mp.	227	Advanced
57	((spine or spinal) adj3 disfunct*).mp.	3	Advanced
58	((spine or spinal) adj3 dysfunct*).mp.	947	Advanced
59	((spine or spinal) adj3 hemisect*).mp.	826	Advanced
60	((spine or spinal) adj3 hemi-sect*).mp.	9	Advanced
61	((spine or spinal) adj3 isch?em*).mp.	2513	Advanced
62	((spine or spinal) adj3 lacerat*).mp.	33	Advanced
63	((spine or spinal) adj3 lesion*).mp.	6521	Advanced
64	((spine or spinal) adj1 monoplegi*).mp.	0	Advanced
65	((spine or spinal) adj3 myelopath*).mp.	367	Advanced
66	((spine or spinal) adj3 (cut or cuts or cutting)).mp.	175	Advanced
67	((spine or spinal) adj3 (sever or severed or severs or severing)).mp.	81	Advanced
68	((spine or spinal) adj3 shock*).mp.	321	Advanced
69	((spine or spinal) adj3 syndrom*).mp.	1797	Advanced
70	((spine or spinal) adj3 transect*).mp.	2791	Advanced
71	((spine or spinal) adj3 wound*).mp.	942	Advanced
72	((spine or spinal) adj3 impair*).mp.	729	Advanced
73	paraplegi*.mp.	17,288	Advanced
74	paraparesis.mp.	5451	Advanced
75	parapareses.mp.	17	Advanced
76	monoplegi*.mp.	148	Advanced
77	hemiplegi*.mp.	14,156	Advanced
78	quadriplegi*.mp.	8758	Advanced
79	quadriparesis.mp.	972	Advanced
80	quadripareses.mp.	2	Advanced
81	tetraplegi*.mp.	3213	Advanced
82	tetraparesis.mp.	1034	Advanced
83	tetrapareses.mp.	5	Advanced
84	brown sequard* syndrom*.mp.	476	Advanced
85	traumatic myelopath*.mp.	55	Advanced
86	brown-sequard* disease*.mp.	1	Advanced
87	brown-sequard* paraly*.mp.	0	Advanced
88	hemicord* syndrome*.mp.	3	Advanced
89	hemi-cord* syndrome*.mp.	0	Advanced
90	hemiparaplegic syndrome*.mp.	0	Advanced
91	hemi-paraplegic syndrome*.mp.	0	Advanced
92	hemispinal cord syndrome*.mp.	0	Advanced
93	hemi-spinal cord syndrome*.mp.	0	Advanced
94	locked in syndrome*.mp.	507	Advanced
95	lockedin syndrome*.mp.	1	Advanced
96	(central cord? adj3 compress*).mp.	1	Advanced
97	(central cord? adj3 contus*).mp.	2	Advanced
98	(central cord? adj3 damag*).mp.	5	Advanced
99	(central cord? adj3 dysfunc*).mp.	2	Advanced
100	(central cord? adj3 disfunc*).mp.	0	Advanced
101	(central cord? adj3 disrupt*).mp.	0	Advanced
102	(central cord? adj3 fractur*).mp.	4	Advanced
103	(central cord? adj3 hemisect*).mp.	2	Advanced
104	(central cord? adj3 hemi-sect*).mp.	0	Advanced
105	(central cord? adj3 injur*).mp.	32	Advanced
106	(central cord? adj3 isch?em*).mp.	1	Advanced
107	(central cord? adj3 lacer*).mp.	0	Advanced
108	(central cord? adj3 lesion*).mp.	11	Advanced
109	(central cord? adj3 myelopath*).mp.	1	Advanced
110	(central cord? adj3 sever*).mp.	2	Advanced
111	(central cord? adj3 shock*).mp.	0	Advanced
112	(central cord? adj3 transect*).mp.	0	Advanced
113	(central cord? adj3 trauma*).mp.	50	Advanced
114	(central cord? adj3 wound*).mp.	0	Advanced
115	flaccid paralysis.mp.	1508	Advanced
116	autonomic dysreflexi*.mp.	702	Advanced
117	(hemisection* adj4 cord?).mp.	542	Advanced
118	(hemi-section* adj4 cord?).mp.	8	Advanced
119	Central Nervous System/and exp wounds/	1040	Advanced
120	Central Nervous System/and in.fs.	633	Advanced
121	(injur* adj3 central nervous system*).mp.	2173	Advanced
122	or/43–121 [Spine/Spinal Cord injuries]	363,737	Advanced
123	42 and 122	4153	Advanced
124	remove duplicates from 123	4033	Advanced
125	animals/not (animals/and humans/)	3,949,562	Advanced
126	124 not 125	3589	Advanced
127	limit 124 to human	3575	Advanced
128	126 or 127	3589	Advanced
129	limit 128 to (“all infant (birth to 23 months)” or “all child (0 to 18 years)” or “newborn infant (birth to 1 month)” or “infant (1 to 23 months)” or “preschool child (2 to 5 years)” or “child (6 to 12 years)” or “adolescent (13 to 18 years)”)	789	Advanced
130	128 not 129	2800	Advanced
131	limit 128 to (“all adult (19 plus years)” or “young adult (19 to 24 years)” or “adult (19 to 44 years)” or “young adult and adult (19–24 and 19–44)” or “middle age (45 to 64 years)” or “middle aged (45 plus years)” or “all aged (65 and over)” or “aged (80 and over)”)	2417	Advanced
132	130 or 131	3240	Advanced
133	limit 132 to english language	2945	Advanced

**Table:** 

vid MEDLINE(R) in-process & other non-indexed citations June 01, 2015			
#	Searches	Results	Search type
1	Diffusion Tensor Imaging/[New MeSH as of 2010]	0	Advanced
2	Diffusion Magnetic Resonance Imaging/	0	Advanced
3	(Diffusion adj1 Tensor? adj1 Imag*).mp.	1298	Advanced
4	(diffusion adj1 weight* adj1 imag*).mp.	947	Advanced
5	(diffusion adj2 MRI?).mp.	612	Advanced
6	(diffusion adj2 MR?).mp.	782	Advanced
7	(diffusion adj2 tractogra*).mp.	125	Advanced
8	(diffusion adj1 coefficien*).mp.	4040	Advanced
9	(diffusion adj1 co-efficien*).mp.	2	Advanced
10	(dti adj2 tractogra*).mp.	63	Advanced
11	dti.tw,kw.	935	Advanced
12	(high* adj1 angular* adj1 resolut* adj1 diffusion*).mp.	41	Advanced
13	hardi.tw,kw.	35	Advanced
14	(magneti#at* adj2 transfer*).mp.	200	Advanced
15	exp magnetic resonance spectroscopy/or electron spin resonance spectroscopy/or nuclear magnetic resonance, biomolecular/	0	Advanced
16	(magnetic adj1 resonan* adj1 spectroscop*).mp.	1558	Advanced
17	(mr adj spectroscop*).mp.	273	Advanced
18	(mri adj spectroscop*).mp.	9	Advanced
19	(functional adj2 (mri or mris or magnetic resonan*)).mp.	3383	Advanced
20	fmri?.mp.	4239	Advanced
21	myelin water fraction*.mp.	10	Advanced
22	1H-MRS.mp.	71	Advanced
23	ADC.tw,kw.	940	Advanced
24	ADT.tw,kw.	316	Advanced
25	(apparent adj1 diffus* adj1 coefficien*).mp.	693	Advanced
26	(apparent adj1 diffus* adj1 tensor*).mp.	0	Advanced
27	(DTI or DTIs).mp.	970	Advanced
28	(fraction* adj1 anisotrop*).mp.	838	Advanced
29	(mean adj1 diffusivit*).mp.	262	Advanced
30	mrs.tw,kw.	1344	Advanced
31	mt ratio?.mp.	7	Advanced
32	mtcsf.mp.	0	Advanced
33	MTR.mp.	151	Advanced
34	MWF.mp.	22	Advanced
35	MWFI.mp.	0	Advanced
36	(NMR adj1 spectroscop*).mp.	7065	Advanced
37	exp Magnetic Resonance Spectroscopy/	0	Advanced
38	(saturat* adj1 spectroscop*).mp.	44	Advanced
39	press.tw,kw.	2535	Advanced
40	(proton* adj1 MRS).mp.	32	Advanced
41	tractograph*.mp.	485	Advanced
42	or/1–41 [Advanced MRI & related terms]	24,201	Advanced
43	exp Spine/	0	Advanced
44	exp Spinal Diseases/	0	Advanced
45	exp Spinal Cord/	0	Advanced
46	exp Spinal Cord Diseases/	0	Advanced
47	exp spinal cord injuries/	0	Advanced
48	exp spinal cord/and exp wounds/	0	Advanced
49	exp spinal cord/and in.fs.	0	Advanced
50	exp paraplegia/or brown-sequard syndrome/	0	Advanced
51	exp quadriplegia/	0	Advanced
52	((spine or spinal) adj3 injur*).mp.	2966	Advanced
53	((spine or spinal) adj3 compress*).mp.	611	Advanced
54	((spine or spinal) adj3 contus*).mp.	94	Advanced
55	((spine or spinal) adj3 damag*).mp.	134	Advanced
56	((spine or spinal) adj3 disrupt*).mp.	25	Advanced
57	((spine or spinal) adj3 disfunct*).mp.	1	Advanced
58	((spine or spinal) adj3 dysfunct*).mp.	100	Advanced
59	((spine or spinal) adj3 hemisect*).mp.	27	Advanced
60	((spine or spinal) adj3 hemi-sect*).mp.	1	Advanced
61	((spine or spinal) adj3 isch?em*).mp.	150	Advanced
62	((spine or spinal) adj3 lacerat*).mp.	3	Advanced
63	((spine or spinal) adj3 lesion*).mp.	469	Advanced
64	((spine or spinal) adj1 monoplegi*).mp.	0	Advanced
65	((spine or spinal) adj3 myelopath*).mp.	34	Advanced
66	((spine or spinal) adj3 (cut or cuts or cutting)).mp.	5	Advanced
67	((spine or spinal) adj3 (sever or severed or severs or severing)).mp.	5	Advanced
68	((spine or spinal) adj3 shock*).mp.	15	Advanced
69	((spine or spinal) adj3 syndrom*).mp.	121	Advanced
70	((spine or spinal) adj3 transect*).mp.	119	Advanced
71	((spine or spinal) adj3 wound*).mp.	35	Advanced
72	((spine or spinal) adj3 impair*).mp.	86	Advanced
73	paraplegi*.mp.	807	Advanced
74	paraparesis.mp.	342	Advanced
75	parapareses.mp.	0	Advanced
76	monoplegi*.mp.	23	Advanced
77	hemiplegi*.mp.	783	Advanced
78	quadriplegi*.mp.	186	Advanced
79	quadriparesis.mp.	149	Advanced
80	quadripareses.mp.	0	Advanced
81	tetraplegi*.mp.	234	Advanced
82	tetraparesis.mp.	63	Advanced
83	tetrapareses.mp.	0	Advanced
84	brown sequard* syndrom*.mp.	46	Advanced
85	traumatic myelopath*.mp.	5	Advanced
86	brown-sequard* disease*.mp.	0	Advanced
87	brown-sequard* paraly*.mp.	1	Advanced
88	hemicord* syndrome*.mp.	0	Advanced
89	hemi-cord* syndrome*.mp.	0	Advanced
90	hemiparaplegic syndrome*.mp.	0	Advanced
91	hemi-paraplegic syndrome*.mp.	0	Advanced
92	hemispinal cord syndrome*.mp.	0	Advanced
93	hemi-spinal cord syndrome*.mp.	0	Advanced
94	locked in syndrome*.mp.	47	Advanced
95	lockedin syndrome*.mp.	0	Advanced
96	(central cord? adj3 compress*).mp.	2	Advanced
97	(central cord? adj3 contus*).mp.	0	Advanced
98	(central cord? adj3 damag*).mp.	0	Advanced
99	(central cord? adj3 dysfunc*).mp.	0	Advanced
100	(central cord? adj3 disfunc*).mp.	0	Advanced
101	(central cord? adj3 disrupt*).mp.	0	Advanced
102	(central cord? adj3 fractur*).mp.	0	Advanced
103	(central cord? adj3 hemisect*).mp.	0	Advanced
104	(central cord? adj3 hemi-sect*).mp.	0	Advanced
105	(central cord? adj3 injur*).mp.	4	Advanced
106	(central cord? adj3 isch?em*).mp.	0	Advanced
107	(central cord? adj3 lacer*).mp.	0	Advanced
108	(central cord? adj3 lesion*).mp.	3	Advanced
109	(central cord? adj3 myelopath*).mp.	2	Advanced
110	(central cord? adj3 sever*).mp.	0	Advanced
111	(central cord? adj3 shock*).mp.	0	Advanced
112	(central cord? adj3 transect*).mp.	0	Advanced
113	(central cord? adj3 trauma*).mp.	3	Advanced
114	(central cord? adj3 wound*).mp.	0	Advanced
115	flaccid paralysis.mp.	141	Advanced
116	autonomic dysreflexi*.mp.	46	Advanced
117	(hemisection* adj4 cord?).mp.	26	Advanced
118	(hemi-section* adj4 cord?).mp.	2	Advanced
119	Central Nervous System/and exp wounds/	0	Advanced
120	Central Nervous System/and in.fs.	0	Advanced
121	(injur* adj3 central nervous system*).mp.	174	Advanced
122	or/43–121 [Spine/Spinal Cord injuries]	6559	Advanced
123	42 and 122	109	Advanced
124	remove duplicates from 123	109	Advanced

**Table:** 

Embase Classic + Embase 1947 to 2015 June 01			
#	Searches	Results	Search type
1	diffusion tensor imaging/	14,569	Advanced
2	diffusion weighted imaging/	22,215	Advanced
3	(Diffusion adj1 Tensor? adj1 Imag*).mp.	15,954	Advanced
4	(diffusion adj1 weight* adj1 imag*).mp.	24,527	Advanced
5	(diffusion adj2 MRI?).mp.	6695	Advanced
6	(diffusion adj2 MR?).mp.	9770	Advanced
7	(diffusion adj2 tractogra*).mp.	1120	Advanced
8	(diffusion adj1 coefficien*).mp.	23,049	Advanced
9	(diffusion adj1 co-efficien*).mp.	48	Advanced
10	(dti adj2 tractogra*).mp.	628	Advanced
11	dti.tw,kw.	10,119	Advanced
12	(high* adj1 angular* adj1 resolut* adj1 diffusion*).mp.	316	Advanced
13	hardi.tw,kw.	280	Advanced
14	(magneti#at* adj2 transfer*).mp.	2957	Advanced
15	exp magnetic resonance spectroscopy/or electron spin resonance spectroscopy/or nuclear magnetic resonance, biomolecular/	255,182	Advanced
16	(magnetic adj1 resonan* adj1 spectroscop*).mp.	112,219	Advanced
17	(mr adj spectroscop*).mp.	5999	Advanced
18	(mri adj spectroscop*).mp.	169	Advanced
19	(functional adj2 (mri or mris or magnetic resonan*)).mp.	55,177	Advanced
20	fmri?.mp.	39,897	Advanced
21	myelin water fraction*.mp.	135	Advanced
22	1H-MRS.mp.	3375	Advanced
23	ADC.tw,kw.	10,338	Advanced
24	ADT.tw,kw.	3399	Advanced
25	(apparent adj1 diffus* adj1 coefficien*).mp.	7945	Advanced
26	(apparent adj1 diffus* adj1 tensor*).mp.	28	Advanced
27	(DTI or DTIs).mp.	10,204	Advanced
28	(fraction* adj1 anisotrop*).mp.	8487	Advanced
29	(mean adj1 diffusivit*).mp.	2489	Advanced
30	mrs.tw,kw.	21,904	Advanced
31	mt ratio?.mp.	258	Advanced
32	mtcsf.mp.	2	Advanced
33	MTR.mp.	2077	Advanced
34	MWF.mp.	483	Advanced
35	MWFI.mp.	1	Advanced
36	(NMR adj1 spectroscop*).mp.	42,596	Advanced
37	exp Magnetic Resonance Spectroscopy/	98,902	Advanced
38	(saturat* adj1 spectroscop*).mp.	18	Advanced
39	press.tw,kw.	215,401	Advanced
40	(proton* adj1 MRS).mp.	758	Advanced
41	tractograph*.mp.	4691	Advanced
42	or/1–41 [Advanced MRI & related terms]	619,716	Advanced
43	spine disease/	13,890	Advanced
44	exp spine injury/	34,151	Advanced
45	exp spinal cord disease/	227,506	Advanced
46	exp spinal cord injury/	59,313	Advanced
47	exp spinal cord injury/	59,313	Advanced
48	brown sequard syndrome/	732	Advanced
49	central cord syndrome/	238	Advanced
50	cervical spinal cord injury/	2498	Advanced
51	spinal cord compression/	13,212	Advanced
52	spinal cord transsection/	2835	Advanced
53	spinal cord transverse lesion/	730	Advanced
54	paraplegia/	22,702	Advanced
55	quadriplegia/	14,032	Advanced
56	((spine or spinal) adj3 injur*).mp.	66,147	Advanced
57	((spine or spinal) adj3 compress*).mp.	17,094	Advanced
58	((spine or spinal) adj3 contus*).mp.	1342	Advanced
59	((spine or spinal) adj3 damag*).mp.	2680	Advanced
60	((spine or spinal) adj3 disrupt*).mp.	315	Advanced
61	((spine or spinal) adj3 disfunct*).mp.	5	Advanced
62	((spine or spinal) adj3 dysfunct*).mp.	1392	Advanced
63	((spine or spinal) adj3 hemisect*).mp.	1291	Advanced
64	((spine or spinal) adj3 hemi-sect*).mp.	17	Advanced
65	((spine or spinal) adj3 isch?em*).mp.	4226	Advanced
66	((spine or spinal) adj3 lacerat*).mp.	46	Advanced
67	((spine or spinal) adj3 lesion*).mp.	13,137	Advanced
68	((spine or spinal) adj1 monoplegi*).mp.	0	Advanced
69	((spine or spinal) adj3 myelopath*).mp.	556	Advanced
70	((spine or spinal) adj3 (cut or cuts or cutting)).mp.	297	Advanced
71	((spine or spinal) adj3 (sever or severed or severs or severing)).mp.	148	Advanced
72	((spine or spinal) adj3 shock*).mp.	599	Advanced
73	((spine or spinal) adj3 syndrom*).mp.	2872	Advanced
74	((spine or spinal) adj3 transect*).mp.	3661	Advanced
75	((spine or spinal) adj3 wound*).mp.	984	Advanced
76	((spine or spinal) adj3 impair*).mp.	1440	Advanced
77	paraplegi*.mp.	31,389	Advanced
78	paraparesis.mp.	6426	Advanced
79	parapareses.mp.	34	Advanced
80	quadriplegi*.mp.	15,727	Advanced
81	quadriparesis.mp.	1554	Advanced
82	quadripareses.mp.	2	Advanced
83	tetraplegi*.mp.	5329	Advanced
84	tetraparesis.mp.	1567	Advanced
85	tetrapareses.mp.	10	Advanced
86	brown sequard* syndrom*.mp.	863	Advanced
87	traumatic myelopath*.mp.	76	Advanced
88	brown-sequard* disease*.mp.	1	Advanced
89	brown-sequard* paraly*.mp.	2	Advanced
90	hemicord* syndrome*.mp.	3	Advanced
91	hemi-cord* syndrome*.mp.	1	Advanced
92	hemiparaplegic syndrome*.mp.	1	Advanced
93	hemi-paraplegic syndrome*.mp.	0	Advanced
94	hemispinal cord syndrome*.mp.	1	Advanced
95	hemi-spinal cord syndrome*.mp.	0	Advanced
96	locked in syndrome*.mp.	1010	Advanced
97	lockedin syndrome*.mp.	6	Advanced
98	(central cord? adj3 (compress* or contus* or damag* or disfunct* or dysfunct* or disrupt* or fractur* or hemisect* or hemi-sect* or injur* or isch?emi* or lacerat* or lesion* or myelopath* or sever* or shock* or transect* or trauma* or wound*)).mp.	155	Advanced
99	flaccid paralysis.mp.	3389	Advanced
100	autonomic dysreflexi*.mp.	1133	Advanced
101	or/43–100	315,291	Advanced
102	42 and 101	7829	Advanced
103	(exp animals/or exp animal experimentation/or nonhuman/) not ((exp animals/or exp animal experimentation/or nonhuman/) and exp human/)	6,051,284	Advanced
104	102 not 103	6955	Advanced
105	limit 102 to human	6444	Advanced
106	104 or 105	6955	Advanced
107	limit 106 to (embryo < first trimester > or infant < to one year > or child < unspecified age > or preschool child < 1 to 6 years > or school child < 7 to 12 years > or adolescent < 13 to 17 years >)	1005	Advanced
108	106 not 107	5950	Advanced
109	limit 106 to (adult < 18 to 64 years > or aged < 65 + years >)	2828	Advanced
110	108 or 109	6316	Advanced
111	limit 110 to embase	6012	Advanced
112	limit 111 to (book or book series or conference abstract or conference proceeding or “conference review”)	1180	Advanced
113	111 not 112	4832	Advanced
114	limit 113 to english language	4413	Advanced
115	remove duplicates from 114	4387	Advanced

**Table:** 

EBM reviews — Cochrane central register of controlled trials April 2015			
#	Searches	Results	Search type
1	Diffusion Tensor Imaging/[New MeSH as of 2010]	38	Advanced
2	Diffusion Magnetic Resonance Imaging/[MeSH]	147	Advanced
3	diffusion tensor imaging/	38	Advanced
4	diffusion weighted imaging/	0	Advanced
5	(Diffusion adj1 Tensor? adj1 Imag*).mp.	157	Advanced
6	(diffusion adj1 weight* adj1 imag*).mp.	219	Advanced
7	(diffusion adj2 MRI?).mp.	98	Advanced
8	(diffusion adj2 MR?).mp.	149	Advanced
9	(diffusion adj2 tractogra*).mp.	15	Advanced
10	(diffusion adj1 coefficien*).mp.	139	Advanced
11	(diffusion adj1 co-efficien*).mp.	0	Advanced
12	(dti adj2 tractogra*).mp.	5	Advanced
13	dti.tw,kw.	139	Advanced
14	(high* adj1 angular* adj1 resolut* adj1 diffusion*).mp.	0	Advanced
15	hardi.tw,kw.	3	Advanced
16	(magneti#at* adj2 transfer*).mp.	65	Advanced
17	exp magnetic resonance spectroscopy/or electron spin resonance spectroscopy/or nuclear magnetic resonance, biomolecular/	471	Advanced
18	(magnetic adj1 resonan* adj1 spectroscop*).mp.	736	Advanced
19	(mr adj spectroscop*).mp.	116	Advanced
20	(mri adj spectroscop*).mp.	11	Advanced
21	(functional adj2 (mri or mris or magnetic resonan*)).mp.	1409	Advanced
22	fmri?.mp.	1210	Advanced
23	myelin water fraction*.mp.	2	Advanced
24	1H-MRS.mp.	54	Advanced
25	ADC.tw,kw.	133	Advanced
26	ADT.tw,kw.	196	Advanced
27	(apparent adj1 diffus* adj1 coefficien*).mp.	121	Advanced
28	(apparent adj1 diffus* adj1 tensor*).mp.	1	Advanced
29	(DTI or DTIs).mp.	139	Advanced
30	(fraction* adj1 anisotrop*).mp.	108	Advanced
31	(mean adj1 diffusivit*).mp.	23	Advanced
32	mrs.tw,kw.	657	Advanced
33	mt ratio?.mp.	12	Advanced
34	mtcsf.mp.	1	Advanced
35	MTR.mp.	50	Advanced
36	MWF.mp.	17	Advanced
37	MWFI.mp.	0	Advanced
38	(NMR adj1 spectroscop*).mp.	72	Advanced
39	exp Magnetic Resonance Spectroscopy/	471	Advanced
40	(saturat* adj1 spectroscop*).mp.	0	Advanced
41	press.tw,kw.	2661	Advanced
42	(proton* adj1 MRS).mp.	20	Advanced
43	tractograph*.mp.	29	Advanced
44	or/1–43 [Advanced MRI & related terms]	6486	Advanced
45	exp spinal cord injury/	751	Advanced
46	brown sequard syndrome/	0	Advanced
47	central cord syndrome/	0	Advanced
48	cervical spinal cord injury/	0	Advanced
49	spinal cord compression/	75	Advanced
50	spinal cord transsection/	0	Advanced
51	spinal cord transverse lesion/	0	Advanced
52	paraplegia/	152	Advanced
53	quadriplegia/	124	Advanced
54	((spine or spinal) adj3 injur*).mp.	1434	Advanced
55	((spine or spinal) adj3 compress*).mp.	255	Advanced
56	((spine or spinal) adj3 contus*).mp.	4	Advanced
57	((spine or spinal) adj3 damag*).mp.	43	Advanced
58	((spine or spinal) adj3 disrupt*).mp.	2	Advanced
59	((spine or spinal) adj3 disfunct*).mp.	0	Advanced
60	((spine or spinal) adj3 dysfunct*).mp.	96	Advanced
61	((spine or spinal) adj3 hemisect*).mp.	0	Advanced
62	((spine or spinal) adj3 hemi-sect*).mp.	0	Advanced
63	((spine or spinal) adj3 isch?em*).mp.	39	Advanced
64	((spine or spinal) adj3 lacerat*).mp.	1	Advanced
65	((spine or spinal) adj3 lesion*).mp.	139	Advanced
66	((spine or spinal) adj1 monoplegi*).mp.	0	Advanced
67	((spine or spinal) adj3 myelopath*).mp.	9	Advanced
68	((spine or spinal) adj3 (cut or cuts or cutting)).mp.	12	Advanced
69	((spine or spinal) adj3 (sever or severed or severs or severing)).mp.	0	Advanced
70	((spine or spinal) adj3 shock*).mp.	9	Advanced
71	((spine or spinal) adj3 syndrom*).mp.	92	Advanced
72	((spine or spinal) adj3 transect*).mp.	4	Advanced
73	((spine or spinal) adj3 wound*).mp.	30	Advanced
74	((spine or spinal) adj3 impair*).mp.	70	Advanced
75	paraplegi*.mp.	324	Advanced
76	paraparesis.mp.	60	Advanced
77	parapareses.mp.	0	Advanced
78	quadriplegi*.mp.	245	Advanced
79	quadriparesis.mp.	5	Advanced
80	quadripareses.mp.	0	Advanced
81	tetraplegi*.mp.	141	Advanced
82	tetraparesis.mp.	5	Advanced
83	tetrapareses.mp.	0	Advanced
84	brown sequard* syndrom*.mp.	0	Advanced
85	traumatic myelopath*.mp.	1	Advanced
86	brown-sequard* disease*.mp.	0	Advanced
87	brown-sequard* paraly*.mp.	0	Advanced
88	hemicord* syndrome*.mp.	0	Advanced
89	hemi-cord* syndrome*.mp.	0	Advanced
90	hemiparaplegic syndrome*.mp.	0	Advanced
91	hemi-paraplegic syndrome*.mp.	0	Advanced
92	hemispinal cord syndrome*.mp.	0	Advanced
93	hemi-spinal cord syndrome*.mp.	0	Advanced
94	locked in syndrome*.mp.	3	Advanced
95	lockedin syndrome*.mp.	0	Advanced
96	(central cord? adj3 (compress* or contus* or damag* or disfunct* or dysfunct* or disrupt* or fractur* or hemisect* or hemi-sect* or injur* or isch?emi* or lacerat* or lesion* or myelopath* or sever* or shock* or transect* or trauma* or wound*)).mp.	0	Advanced
97	flaccid paralysis.mp.	4	Advanced
98	autonomic dysreflexi*.mp.	21	Advanced
99	exp spinal cord injuries/	751	Advanced
100	exp spinal cord/and exp wounds/	32	Advanced
101	exp spinal cord/and in.fs.	7	Advanced
102	Central Nervous System/and exp wounds/	10	Advanced
103	Central Nervous System/and in.fs.	3	Advanced
104	exp spine disease/	0	Advanced
105	exp spine injury/	0	Advanced
106	or/45–105	2334	Advanced
107	44 and 106	25	Advanced
108	limit 107 to medline records	11	Advanced
109	limit 107 to embase records	12	Advanced
110	108 or 109	23	Advanced
111	107 not 110	2	Advanced

**Table:** 

EBM reviews — Cochrane database of systematic reviews 2005 to April 2015			
#	Searches	Results	Search type
1	[Diffusion Tensor Imaging/[New MeSH as of 2010]]	0	Advanced
2	[Diffusion Magnetic Resonance Imaging/[MeSH]]	0	Advanced
3	[diffusion tensor imaging/]	0	Advanced
4	[diffusion weighted imaging/]	0	Advanced
5	(Diffusion adj1 Tensor? adj1 Imag*).mp.	2	Advanced
6	(diffusion adj1 weight* adj1 imag*).mp.	9	Advanced
7	(diffusion adj2 MRI?).mp.	8	Advanced
8	(diffusion adj2 MR?).mp.	9	Advanced
9	(diffusion adj2 tractogra*).mp.	0	Advanced
10	(diffusion adj1 coefficien*).mp.	1	Advanced
11	(diffusion adj1 co-efficien*).mp.	0	Advanced
12	(dti adj2 tractogra*).mp.	1	Advanced
13	dti.tw,kw.	14	Advanced
14	(high* adj1 angular* adj1 resolut* adj1 diffusion*).mp.	0	Advanced
15	hardi.tw,kw.	0	Advanced
16	(magneti#at* adj2 transfer*).mp.	3	Advanced
17	[exp magnetic resonance spectroscopy/or electron spin resonance spectroscopy/or nuclear magnetic resonance, biomolecular/]	0	Advanced
18	(magnetic adj1 resonan* adj1 spectroscop*).mp.	21	Advanced
19	(mr adj spectroscop*).mp.	1	Advanced
20	(mri adj spectroscop*).mp.	0	Advanced
21	(functional adj2 (mri or mris or magnetic resonan*)).mp.	30	Advanced
22	fmri?.mp.	22	Advanced
23	myelin water fraction*.mp.	0	Advanced
24	1H-MRS.mp.	0	Advanced
25	ADC.tw,kw.	3	Advanced
26	ADT.tw,kw.	6	Advanced
27	(apparent adj1 diffus* adj1 coefficien*).mp.	1	Advanced
28	(apparent adj1 diffus* adj1 tensor*).mp.	0	Advanced
29	(DTI or DTIs).mp.	15	Advanced
30	(fraction* adj1 anisotrop*).mp.	0	Advanced
31	(mean adj1 diffusivit*).mp.	0	Advanced
32	mrs.tw,kw.	290	Advanced
33	mt ratio?.mp.	0	Advanced
34	mtcsf.mp.	0	Advanced
35	MTR.mp.	2	Advanced
36	MWF.mp.	0	Advanced
37	MWFI.mp.	0	Advanced
38	(NMR adj1 spectroscop*).mp.	0	Advanced
39	[exp Magnetic Resonance Spectroscopy/]	0	Advanced
40	(saturat* adj1 spectroscop*).mp.	0	Advanced
41	press.tw,kw.	624	Advanced
42	(proton* adj1 MRS).mp.	1	Advanced
43	tractograph*.mp.	1	Advanced
44	or/1–43 [Advanced MRI & related terms]	972	Advanced
45	[exp spinal cord injury/]	0	Advanced
46	[brown sequard syndrome/]	0	Advanced
47	[central cord syndrome/]	0	Advanced
48	[cervical spinal cord injury/]	0	Advanced
49	[spinal cord compression/]	0	Advanced
50	[spinal cord transsection/]	0	Advanced
51	[spinal cord transverse lesion/]	0	Advanced
52	[paraplegia/]	0	Advanced
53	[quadriplegia/]	0	Advanced
54	((spine or spinal) adj3 injur*).mp.	177	Advanced
55	((spine or spinal) adj3 compress*).mp.	40	Advanced
56	((spine or spinal) adj3 contus*).mp.	8	Advanced
57	((spine or spinal) adj3 damag*).mp.	34	Advanced
58	((spine or spinal) adj3 disrupt*).mp.	5	Advanced
59	((spine or spinal) adj3 disfunct*).mp.	0	Advanced
60	((spine or spinal) adj3 dysfunct*).mp.	13	Advanced
61	((spine or spinal) adj3 hemisect*).mp.	0	Advanced
62	((spine or spinal) adj3 hemi-sect*).mp.	0	Advanced
63	((spine or spinal) adj3 isch?em*).mp.	18	Advanced
64	((spine or spinal) adj3 lacerat*).mp.	3	Advanced
65	((spine or spinal) adj3 lesion*).mp.	28	Advanced
66	((spine or spinal) adj1 monoplegi*).mp.	0	Advanced
67	((spine or spinal) adj3 myelopath*).mp.	6	Advanced
68	((spine or spinal) adj3 (cut or cuts or cutting)).mp.	2	Advanced
69	((spine or spinal) adj3 (sever or severed or severs or severing)).mp.	0	Advanced
70	((spine or spinal) adj3 shock*).mp.	3	Advanced
71	((spine or spinal) adj3 syndrom*).mp.	17	Advanced
72	((spine or spinal) adj3 transect*).mp.	2	Advanced
73	((spine or spinal) adj3 wound*).mp.	10	Advanced
74	((spine or spinal) adj3 impair*).mp.	14	Advanced
75	paraplegi*.mp.	39	Advanced
76	paraparesis.mp.	13	Advanced
77	parapareses.mp.	0	Advanced
78	quadriplegi*.mp.	33	Advanced
79	quadriparesis.mp.	0	Advanced
80	quadripareses.mp.	0	Advanced
81	tetraplegi*.mp.	14	Advanced
82	tetraparesis.mp.	4	Advanced
83	tetrapareses.mp.	0	Advanced
84	brown sequard* syndrom*.mp.	1	Advanced
85	traumatic myelopath*.mp.	2	Advanced
86	brown-sequard* disease*.mp.	0	Advanced
87	brown-sequard* paraly*.mp.	0	Advanced
88	hemicord* syndrome*.mp.	0	Advanced
89	hemi-cord* syndrome*.mp.	0	Advanced
90	hemiparaplegic syndrome*.mp.	0	Advanced
91	hemi-paraplegic syndrome*.mp.	0	Advanced
92	hemispinal cord syndrome*.mp.	0	Advanced
93	hemi-spinal cord syndrome*.mp.	0	Advanced
94	locked in syndrome*.mp.	3	Advanced
95	lockedin syndrome*.mp.	0	Advanced
96	(central cord? adj3 (compress* or contus* or damag* or disfunct* or dysfunct* or disrupt* or fractur* or hemisect* or hemi-sect* or injur* or isch?emi* or lacerat* or lesion* or myelopath* or sever* or shock* or transect* or trauma* or wound*)).mp.	6	Advanced
97	flaccid paralysis.mp.	10	Advanced
98	autonomic dysreflexi*.mp.	7	Advanced
99	[exp spinal cord injuries/]	0	Advanced
100	[exp spinal cord/and exp wounds/]	0	Advanced
101	[exp spinal cord/and in.fs.]	0	Advanced
102	[Central Nervous System/and exp wounds/]	0	Advanced
103	[Central Nervous System/and in.fs.]	0	Advanced
104	[exp spine disease/]	0	Advanced
105	[exp spine injury/]	0	Advanced
106	or/45–105	306	Advanced
107	44 and 106	40	Advanced
108	limit 107 to full systematic reviews	31	Advanced

**Table:** 

Authors (year)	Study purpose	All studies							Diagnostic studies	Correlation studies	Prognostic studies				Risk of bias
Prospective cohort design	Demographic/confounding factors matched/analyzed	Homogenous diagnosis	Random/consecutive enrollment	Acquisition techniques highly reliable	Metrics derived using automated/blinded techniques	Range of severity	Diagnostic accuracy reported	Univariate or multivariate correlations reported	Similar point in course of disease	Follow-up > 80%	Follow-up long enough for outcomes to occur	Accounts for known prognostic factors
Abbas et al. (2015)	Diagnostic	✔	–	✔	✔	–	–	✔	–	–					High
Correlation	High
Agosta et al. (2005)	Diagnostic	✔	✔	✔	–	–	–	✔	–	✔					High
Correlation	High
Agosta et al. (2006)	Diagnostic	✔	✔	✔	✔	✔	–	✔	–						Moderately high
Agosta et al. (2007a)	Correlation	✔	–	✔	–	–	–	✔		✔	–	–	✔	✔	High
Prognostic	High
Agosta et al. (2007b)	Diagnostic	✔	✔	✔	–	✔	–	✔	–	✔					High
Correlation	Moderately high
Agosta et al. (2008a)	Diagnostic	✔	✔	✔	–	–	–	✔	–						High
Agosta et al. (2008b)	Diagnostic	✔	✔	✔	–	–	–	✔	–						High
Agosta et al. (2009a)	Correlation	✔	✔	✔	–	–	–	✔		✔					High
Agosta et al. (2009b)	Diagnostic	✔	✔	✔	–	–	–	✔	–						High
Banaszek et al. (2014)	Diagnostic	✔	–	✔	–	–	–	✔	–						High
Bellenberg et al. (2013)	Diagnostic	✔	✔	✔	–	–	✔	✔	–	✔	–	✔	–	–	High
Correlation	Moderately high
Prognostic	High
Benedetti et al. (2010)	Diagnostic	✔	✔	✔	✔	–	–	✔	–	✔					High
Correlation	Moderately high
Bozzali et al. (1999)	Diagnostic	✔	✔	✔	–	✔	–	✔	–	–					High
Correlation	High
Budzik et al. (2011)	Diagnostic	✔	✔	✔	–	–	–	✔	–	–					High
Correlation	High
Cadotte et al. (2012)	Diagnostic	✔	–	✔	–	–	✔	✔	–	✔					High
Correlation	High
Carew et al. (2011a)	Diagnostic	✔	–	✔	–	–	✔	✔	–	✔					High
Correlation	High
Carew et al. (2011b)	Diagnostic	✔	✔	✔	–	–	✔	–	–						High
Cheran et al. (2011)	Diagnostic	✔	–	✔	✔	✔	–	✔	–	✔					High
Correlation	Moderately high
Ciccarelli et al. (2007)	Diagnostic	✔	–	✔	✔	✔/– DTI/MRS	–/✔ DTI/MRS	✔	–	✔					High
Correlation	Moderately high
Ciccarelli et al. (2010a)	Diagnostic	✔	–	✔	–	✔/– DTI/MRS	–/✔ DTI/MRS	✔	–	✔					High
Correlation	High
Ciccarelli et al. (2010b)	Correlation	✔	–	✔	–	–	✔	✔		✔	–	✔	–	✔	High
Prognostic	High
Cohen-Adad et al. (2011)	Diagnostic	✔	✔	✔	–	✔	–	✔	–	✔					High
Correlation	Moderately high
Cohen-Adad et al. (2013)	Diagnostic	✔	–	✔	–	✔	–	✔	–	✔					High
Correlation	High
Cruz et al. (2009)	Diagnostic	–	✔	✔	–	–	–	–	–						High
Cui et al. (2015)	Diagnostic	✔	–	✔	–	–	✔	✔	–						High
Demir et al. (2003)	Diagnostic	✔	–	✔	–	–	–	✔	✔						High
El Mendili et al. (2014)	Correlation	✔	✔	✔	–	✔	–	✔		✔	✔	✔	✔	✔	Moderately high
Prognostic	Moderately high
Ellingson et al. (2014)	Diagnostic	✔	–	✔	–	–	–	✔	✔	✔					High
Correlation	High
Facon et al. (2005)	Diagnostic	✔	–	–	–	✔	–	✔	✔						High
Fatemi et al. (2005)	Diagnostic	✔	✔	✔	–	✔	–	✔	–	✔					High
Correlation	Moderately high
Filippi et al. (2000)	Diagnostic	✔	✔	✔	–	✔	–	✔	–	–					High
Correlation	High
Filippi et al. (2002)	Diagnostic	✔	✔	✔	–	✔/– DTI/fMRI	–	✔	–	✔					High
Correlation	Moderately high (MT), high (fMRI)
Freund et al. (2010)	Prognostic	✔	✔	✔	–	✔	–	✔			✔	✔	✔	✔	Moderately high
Gao et al. (2013)	Correlation	✔	–	✔	–	–	–	✔		✔					High
Hatem et al. (2009)	Diagnostic	✔	✔	✔	–	–	–	✔	–	✔					High
Correlation	High
Hatem et al. (2010)	Diagnostic	✔	✔	✔	–	–	–	✔	–	✔					High
Correlation	High
Hesseltine et al. (2006)	Diagnostic	✔	✔	✔	–	✔	–	–	✔						High
Hodel et al. (2013)	Diagnostic	✔	✔	–	–	–	–	✔	–	✔					High
Correlation	High
Holly et al. (2009)	Diagnostic	✔	✔	✔	–	–	✔	✔	–	✔					High
Correlation	Moderately high
Hori et al. (2012)	Diagnostic	✔	–	✔	✔	–	–	–	–						High
Iglesias et al. (2015)	Diagnostic	✔	✔	✔	–	–	–	✔	–	✔					High
Correlation	High
Ikeda et al. (2013)	Diagnostic	✔	✔	✔	–	–	✔	✔	–	✔	✔	–	✔	–	High
Correlation	Moderately high
Prognostic	High
Inglese et al. (2001)	Diagnostic	✔	✔	✔	–	✔	–	✔	–						High
Jeantroux et al. (2012)	Diagnostic	✔	✔	✔	–	–	–	✔	–						High
Jones et al. (2013)	Correlation	✔	✔	✔	–	✔	–	✔		✔	✔	✔	–	✔	Moderately high
Prognostic	High
Kamble et al. (2011)	Diagnostic	✔	–	✔	–	–	–	✔	–						High
Kearney et al. (2014)	Diagnostic	✔	✔	✔	–	✔	✔	✔	–	✔					Moderately high
Correlation	Moderately low
Kearney et al. (2015)	Diagnostic	✔	✔	✔	–	✔	✔	✔	–	✔					Moderately high
Correlation	Moderately low
Kerkovsky et al. (2012)	Diagnostic	✔	✔	✔	–	–	–	✔	–						High
Koskinen et al. (2013)	Diagnostic	✔	–	✔	✔	✔	–	✔	–	✔					High
Correlation	Moderately high
Laule et al. (2010)	Diagnostic	✔	✔	✔	–	–	–	✔	–	✔	✔	✔	✔	✔	High
Correlation	High
Prognostic	High
Lee et al. (2011)	Diagnostic	✔	✔	✔	✔	–	–	✔	–	–	✔	–	–	–	High
Correlation	High
Prognostic	High
Li et al. (2014)	Diagnostic	✔	–	✔	–	–	–	✔	✔						High
Lindberg et al. (2012)	Diagnostic	✔	–	✔	–	✔	–	–	–						High
Lycklama et al. (2000)	Diagnostic	✔	–	✔	–	–	–	✔	–	✔					High
Correlation	High
Maki et al. (2015)	Diagnostic	✔	–	✔	–	✔	–	✔	–	✔					High
Correlation	High
Mamata et al. (2005)	Diagnostic	✔	–	✔	–	–	–	–	–						High
Manconi et al. (2008)	Diagnostic	✔	–	✔	✔	–	–	✔	–						High
Marliani et al. (2010)	Diagnostic	✔	–	✔	–	–	✔	–	–	✔					High
Correlation	High
Miraldi et al. (2013)	Diagnostic	✔	✔	✔	–	–	–	✔	–	✔					High
Correlation	High
Mueller-Mang et al. (2011)	Diagnostic	✔	–	✔	–	–	–	–	–						High
Nair et al. (2010)	Diagnostic	✔	–	✔	–	✔	✔	✔	–	✔					High
Correlation	Moderately high
Naismith et al. (2013)	Diagnostic	✔	–	–	–	✔	–	✔	–	✔					High
Correlation	High
Oh et al. (2013a)	Correlation	✔	✔	✔	–	✔	✔	✔		–					Moderately high
Oh et al. (2013b)	Diagnostic	✔	✔	✔	–	✔	✔	✔	–	✔					Moderately high
Correlation	Moderately low
Oh et al. (2015)	Diagnostic	✔	✔	✔	–	✔	✔	✔	–	✔					Moderately high
Correlation	Moderately low
Ohgiya et al. (2007)	Diagnostic	✔	✔	✔	–	✔	–	–	–						High
Pessoa et al. (2012)	Diagnostic	✔	✔	✔	–	–	–	✔	–	✔					High
Correlation	High
Petersen et al. (2012)	Diagnostic	✔	✔	✔	–	✔	–	✔	–	✔					High
Correlation	Moderately high
Rajasekaran et al. (2014)	Diagnostic	✔	–	✔	–	✔	–	✔	–	✔					High
Correlation	High
Raz et al. (2013)	Diagnostic	✔	✔	✔	✔	✔	–	✔	–	✔					Moderately high
Correlation	Moderately low
Renoux et al. (2006)	Diagnostic	✔	–	–	–	✔	–	–	–						High
Rocca et al. (2001)	Diagnostic	✔	✔	✔	–	✔	–	✔	–	✔					High
Correlation	Moderately high
Rocca et al. (2006)	Diagnostic	✔	✔	✔	–	✔	–	✔	–	✔					High
Correlation	Moderately high
Rocca et al. (2012)	Diagnostic	✔	✔	✔	–	✔/– DTI/fMRI	–	✔	–	✔					High
Correlation	Moderately high (DTI)/high (fMRI)
Rovaris et al. (2000)	Correlation	✔	✔	✔	–	✔	–	✔		–					High
Rovaris et al. (2001a)	Diagnostic	✔	✔	✔	✔	✔	–	–	–						High
Rovaris et al. (2001b)	Diagnostic	✔	✔	✔	✔	✔	–	✔	–	✔					Moderately high
Correlation	Moderately low
Rovaris et al. (2004)	Diagnostic	✔	✔	✔	–	✔	–	✔	–						High
Rovaris et al. (2008)	Diagnostic	✔	✔	✔	–	✔	–	✔	–						High
Salamon et al. (2013)	Diagnostic	✔	–	✔	–	–	✔	✔	–	✔					High
Correlation	High
Shanmuganathan et al. (2008)	Diagnostic	–	–	–	–	–	–	–	–						High
Silver et al. (1997)	Diagnostic	✔	✔	✔	–	–	✔	✔	–	–					High
Correlation	High
Song et al. (2011)	Diagnostic	✔	✔	✔	✔	–	–	–	–						High
Stroman et al. (2004)	Diagnostic	✔	–	✔	–	–	–	–	–						High
Taha Ali and Badawy (2013)	Diagnostic	✔	–	✔	–	–	–	–	–						High
Toosy et al. (2014)	Diagnostic	✔	✔	✔	–	–	✔	✔	–	✔					High
Correlation	Moderately high
Uda et al. (2013)	Diagnostic	✔	–	–	✔	✔	–	–	✔						High
Ukmar et al. (2012)	Diagnostic	✔	–	✔	–	–	–	–	–	–					High
Correlation	High
Valsasina et al. (2005)	Diagnostic	✔	✔	✔	–	–	–	✔	–	✔					High
Correlation	High
Valsasina et al. (2007)	Diagnostic	✔	✔	✔	✔	–	–	✔	–	✔					High
Correlation	Moderately high
Valsasina et al. (2010)	Diagnostic	✔	✔	✔	–	–	–	✔	–	–					High
Correlation	High
Valsasina et al. (2012)	Diagnostic	✔	✔	✔	–	–	–	–	–	✔					High
Correlation	High
van Hecke et al. (2009)	Diagnostic	✔	✔	✔	–	✔	✔	–	–						High
Vedantam et al. (2015)	Diagnostic	–	✔	✔	–	–	–	✔	–	✔					High
Correlation	High
von Meyenburg et al. (2013)	Diagnostic	✔	–	✔	–	✔	–	✔	–	✔					High
Correlation	High
Wang et al. (2012)	Diagnostic	✔	–	✔	–	–	–	✔	–	✔					High
Correlation	High
Wang et al. (2014)	Diagnostic	✔	✔	✔	–	–	–	✔	–	✔					High
Correlation	High
Wen et al. (2014a)	Diagnostic	✔	–	✔	–	✔	–	–	–						High
Wen et al. (2014b)	Diagnostic	✔	–	✔	–	✔	–	✔	–	✔	✔	✔	✔	–	High
Correlation	High
Prognostic	High
Xiangshui et al. (2010)	Diagnostic	✔	✔	✔	–	–	–	–	–						High
Yan et al. (2015)	Diagnostic	✔	✔	✔	–	–	–	–	–						High
Zackowski et al. (2009)	Diagnostic	✔	–	✔	–	✔	–	✔	–	✔					High
Correlation	High
Zhou et al. (2014)	Diagnostic	✔	✔	✔	–	✔	–	✔	–						High

**Table 1:** Study inclusion and exclusion criteria.

	Inclusion	Exclusion
Patient	• Studies involving adult or pediatric human population (no age restriction) • Studies that include patients with a known or suspected pathological diagnosis affecting the spinal cord (SCI, CSM, MS, ALS, infarction, tumor, etc.)	• Animal subjects • Studies in only healthy subjects
Prognostic factors	• Metrics derived from spinal cord DTI: FA, MD, AD, RD • Metrics derived from spinal cord DTI tractography: fiber length, fiber density • Metrics derived from spinal cord MT imaging: MTR or MTCSF • Metrics derived from spinal cord MWF imaging • Metrics derived from spinal cord MRS: absolute or relative (expressed as a ratio) metabolite concentrations • Metrics derived fMRI signal conduction loss	• Studies involving brain imaging techniques
Outcome	• Diagnosis by disease specific criteria (e.g. McDonald criteria for MS) • Clinical severity by validated clinical tools/measures (e.g. ASIA for SCI, JOA/mJOA score for CSM, EDSS for MS, etc.) • Outcomes by disease-specific measures or quality of life measures (e.g. SF-36)	• Subjective or unvalidated outcome measures
Study Design	• Restrospective or prospective cohort studies designed to assess the ability of an imaging factor to: ○ Make a diagnosis ○ Correlate with neurological/functional impairment ○ Predict neurological/functional outcome after at least 3 months • Minimum 24 total subjects, with at least 12 having spinal pathological condition of interest	• Review articles • Opinions • Technical reports • Studies in healthy controls • Animal or biomechanical studies

**Table 2:** Risk of bias for diagnostic, correlation, and prognostic advanced MRI studies.

Risk of bias	Study design	Criteria for diagnostic studies	Criteria for correlation (biomarker) studies	Criteria for prognostic studies
Low risk: Study adheres to commonly held tenets of high quality design, execution and avoidance of bias	Good quality cohort*	• Prospective cohort design • Demographic and other potentially confounding information (age, gender, duration of disease) reported and matched/analyzed • Cohort includes patients with a homogeneous diagnosis • Patients have a range of severity of disease including mild/early (non-obvious) cases • Patients are randomly selected or recruited consecutively (on admission or in clinic) • Acquisition techniques likely to produce reliable results (acceptable SNR and distortions) • Quantitative MRI metrics derived using automated or blinded techniques • Objective criteria used for diagnosis based on presence/absence of distinct features or measurements • Appropriate reporting of SE, SP, PPV, NPV and/or ROC curves	• Prospective cohort design • Demographic and other potentially confounding information (age, gender, duration of disease) reported and matched/analyzed • Cohort includes patients with a homogeneous diagnosis • Patients have a range of severity of disease including mild/early (non-obvious) cases • Patients are randomly selected or recruited consecutively (on admission or in clinic) • Acquisition techniques likely to produce reliable results (acceptable SNR and distortions) • Quantitative MRI metrics derived using automated or blinded techniques • Calculation of univariate correlation coefficients (Spearman or Pearson) or multivariate regression analysis on quantitative imaging features, related to clinical measures	• Prospective longitudinal cohort design • Demographic and other potentially confounding information (age, gender, duration of disease) reported and matched/analyzed • Patients are randomly selected or recruited consecutively (on admission or in clinic) • Cohort includes patients with a homogeneous diagnosis • Patients at reasonably similar point in the course of their disease or treatment (**differs from diagnostic and correlation studies) • F/U rate of greater than 80% • Patients followed long enough for outcomes to occur • Accounts for other known prognostic factors • Acquisition techniques likely to produce reliable results (acceptable SNR and distortions) • Quantitative MRI metrics derived using automated or blinded techniques
Moderately low risk: Study has potential for some bias; does not meet all criteria for class I but deficiencies not likely to invalidate results or introduce significant bias	Moderate quality cohort	• A cohort study that violates one of the criteria for low risk of bias	• A cohort study that violates one of the criteria for low risk of bias	• Prospective design, with violation of one of the other criteria for good quality cohort study • Retrospective design, meeting all the rest of the criteria for low risk of bias
Moderately high risk: Study has flaws in design and/or execution that increase potential for bias that may invalidate study results	Poor quality cohort, good quality case–control or cross-sectional (prognostic only)	• A cohort study that violates two of the criteria for low risk of bias • A case–control study that violates one of the other criteria for low risk of bias	• A cohort study that violates two of the criteria for low risk of bias • A case–control study that violates one of the other criteria for low risk of bias	• Prospective design with violation of 2 or more criteria for good quality cohort • Retrospective design with violation of 1 or more criteria for good quality cohort • A good case–control study • A good cross-sectional study
High risk: Study has significant potential for bias; does not include design features geared toward minimizing bias and/or does not have a comparison group	Very poor quality cohort, poor quality case–control or cross-sectional (prognostic only), case series	• A cohort study that violates three or more of the criteria for low risk of bias • A case–control study that violates two of the other criteria for low risk of bias • Any case series design	• A cohort study that violates three or more of the criteria for low risk of bias • A case–control study that violates two of the other criteria for low risk of bias • Any case series design	• Other than a good case–control study • Other than a good cross-sectional study • Any case series design

**Table 3:** Summary of ROI-based quantitative DTI studies.

Authors (year); design	Subjects	B_0_; vendor; coil; gradients	Anatomical region/position	DTI acquisition	FOV; matrix; voxel size; TR/TE (ms); cardiac gating; AT	DTI metrics	ROI	Clinical measures	Key results	Risk of bias; key barriers to translation
Demir et al (2003); prospective, cross-sectional	CSM (36 total, 21 with myelopathy) vs. HCs (8)	1.5 T; Philips; surface coil; 23 mT/m	• C1–C7 • 3 sagittal slices, 1 mm gap	• SE multishot EPI, 13 echoes • 6 directions • b = 300,600 s/mm^2^	240 mm^2^; 256 × 195; 0.9 × 1.2 × 5 mm^3^; 3 beats/36; yes; 13 min	FA, MD	Manual, whole cord at MCL and NASC	• Presence of myelopathy • SSEPs	• To detect clinical/SSEP myelopathy, MD had SE = 92%, SP = 50%, PPV = 80%, NPV = 75%, and FA had SE = 90%, SP = 50%, PPV = 76%, NPV = 75% • MD, FA had higher SE but lower SP than T2w changes	High; minimal clinical data, several subjects excluded due to low SNR
Agosta et al. (2005); prospective, cross-sectional	PPMS (24) vs. HCs (13)	1.5 T; Siemens; phased-array spine coil	• C1–C7 • 5 sagittal slices, contiguous	• ssEPI, SENSE = 2 • 3 sat bands • Repeated 4 × • 14 directions • b = 900 s/mm^2^	240 × 90 mm^2^; 128 × 48; 1.9 × 1.9 × 4 mm^3^; 7000/100; no; AT NR	FA, MD (corrected with CSA)	Manual ROI, mid-sagittal slice, excluding edge voxels	• EDSS	• Reduced mean FA: 0.38 vs. 0.42, P = 0.007 • Increased MD: 1.20 vs. 1.28 (P = 0.024) • No correlations of DTI metrics found with EDSS	High; coarse clinical data, large voxels increase partial volume effect
Facon et al. (2005); prospective, cross-sectional	CM (15 total, 6 CSM, 5 abscess, 4 tumor) vs. HCs (11)	1.5 T; NR; NR; NR	• Cervical, thoracic • 12 sagittal slices, contiguous	• ssEPI, GRAPPA = 2 • 6 directions • b = 500 s/mm^2^	179 mm^2^; 128 × 128; 1.4 × 1.4 × 3 mm^3^; 4600/73; no; 7 min (3 acquisitions)	FA, MD	Manual, at MCL (CM) or averaged over all levels (HCs)	• Presence of pain, motor or sensory impairment	• No effect of rostrocaudal level seen on FA, MD • FA lower at compressed levels (0.67) than normal appearing cord (0.74, P = 0.01) and controls (0.75, P = 0.01) • FA had better SE (73%) and SP (100%) than T2w-HI or ADC	High; heterogeneous population, metrics at MCL potentially biased
Mamata et al. (2005); prospective, cross-sectional	CSM (79) vs. HCs (11)	1.5 T; GE; spine PAC; 22 mT/m or 40 mT/m	• C1–C7 • 1 sagittal slice	• Sagittal line scan • b = 5 s/mm^2^ taken in 2 directions • 6 directions • b = 1000 s/mm^2^	220 × 110 mm^2^; 128 × 128; 1.7 × 1.7 × 4 mm^3^; 2733/86; no; 31 s per slice	FA, MD	Manual, 2 ROIs drawn at C2–3 and at MCL (or C4–C7 in HCs)	• None	• 54% of spondylosis subjects have low FA, high MD • Age correlates with FA (r = − 0.24) and MD (r = 0.24) • FA is decreased, MD increased within T2 hyper-intensity (P < 0.05)	High; no clinical data, single mid-sagittal slice misses key WM tracts
Valsasina et al. (2005); prospective, cross-sectional	MS (44 total, 21 RRMS, 23 SPMS) vs. HCs (17)	Same as Agosta et al. (2005)					Manual, drawn on mid-sagittal slice	• EDSS	• Reduced mean FA: 0.36 vs. 0.43, P = 0.008 • FA not different in SPMS vs. RRMS • FA correlates with EDSS: r = − 0.48, P = 0.001 • MD correlates with EDSS: r = 0.37, P = 0.02	High; coarse clinical data, single mid-sagittal slice misses key WM tracts
Hesseltine et al. (2006); prospective, cross-sectional	RRMS (24) vs. HCs (24)	1.5 T; NR; NR; NR	• C2–C3 • 10 axial slices, contiguous	• SE EPI • 6 directions • b = 1000 s/mm^2^	140 mm^2^; 128 × 128; 1.1 × 1.1 × 4 mm^3^; 2000/74; no; 2 min 20 s	FA, MD	Manual, 7 ROIs at C2–3: bilateral STTs, LCSTs, DCs, and central cord	• None	• FA decreased in LCSTs (P < 0.0001) and DCs (P = 0.001) • Model using spatial FA data has SE = 87%, SP = 92%	High; no clinical data
Renoux et al. (2006); prospective, cross-sectional	Myelitis (15 total, 9 MS, 6 other) vs. HCs (11)	1.5 T; Philips; NR; 23 mT/m	• C2–C5, T1–T6, T7–T12 • 3 sagittal slices, 1 mm gap	• Multi-shot EPI • 25 directions • b = 300, 600 s/mm^2^	240 mm^2^; 256 × 195; 0.9 × 1.2 × 5 mm^3^; 3 beats/80; yes; NR	FA, MD (calculated as z-statistics)	Manual, whole-cord (avoiding edge voxels)	• None	• All T2 hyperintense lesions had significantly decreased FA • 9 subjects showed significant FA decrease in normal-appearing SC, and 5 had areas of increased FA	High; no clinical data, no correction for multiple comparisons
Agosta et al. (2007a); prospective, longitudinal	MS (42 total, 13 RRMS, 14 SPMS, 15 PPMS) vs. HCs (9)	Same as Agosta et al. (2005)						• EDSS • FU at 1.5–3 years (mean 2.4)	• At FU, FA decreased: 0.36 vs. 0.37, P = 0.01 • At FU, MD increased: 1.26 vs. 1.37, P < 0.001 • Cord FA correlates with EDSS: r = − 0.51, P = 0.001 • Cord FA decrease was greatest in PPMS: P = 0.05 • Baseline FA predicts EDSS at FU: r = − 0.40, P = 0.03	High; coarse clinical data
Ohgiya et al. (2007); prospective, cross-sectional	MS (21 total, 16 RRMS, 4 SPMS, 1 PPMS) vs. HCs (21)	1.5 T; GE; 8-channel neuro-vascular PAC	• C2–C5 • Axial slices, number NR, contiguous	• ssEPI • 25 directions • b = 900 s/mm^2^	170 mm^2^; 128 × 128; 1.3 × 1.3 × 4 mm^3^; 12,000/107; no; 6 min	FA, MD	Manual, ROIs drawn on plaques and NAWM (DCs and R/L LCs), matched in HCs	• None	• FA decreased in all ROIs vs. HCs (all P < 0.001) • MD increased in 6/9 ROIs (P < 0.05) • FA decreased in plaques vs. NAWM vs. HCs (0.44 vs. 0.54 vs. 0.74, P < 0.01)	High; no clinical data
Valsasina et al. (2007); prospective, cross-sectional	ALS (28) vs. HCs (20)	1.5 T; Siemens; spine PAC; 33 mT/m, 125 mT/m/ms	• C1–C7 • 5 sagittal slices, 1.2 mm gap	• ssEPI • 12 directions • 3 sat bands • Repeated 2 × • b = 900 s/mm^2^	240 × 90 mm^2^; 128 × 48; 1.9 × 1.9 × 4 mm^3^; 2900/84; no; NR	FA, MD (with and without correction for CSA)	Semi-automated segmentation, manual ROI of cord excluding edge voxels	• ALSFRS • FU at 6–12 months (mean 9)	• Decreased mean FA: 0.48 vs. 0.52, P = 0.002 • MD not different than controls: 0.88 vs. 0.85, NS • Mean FA correlates with ALSFRS, r = 0.74, P < 0.001	High (diagnostic), moderately high (correlation); gaps in sagittal acquisition exclude some WM
Agosta et al. (2008b); prospective, cross-sectional	RRMS (25) vs. HCs (12)	1.5 T; Siemens; spine PAC; 33 mT/m, 125 mT/m/ms	• C1–C7 • 5 sagittal slices, contiguous	• ssEPI • 12 directions • 3 sat bands • Repeated 4 × • b = 900 s/mm^2^	240 × 180 mm^2^; 192 × 144; 1.3 × 1.3 × 4 mm^3^; 2700/71; no; NR	FA, MD (with and without correction for CSA)	Semi-automated segmentation, manual ROI of cord excluding edge voxels	• EDSS	• Decreased mean FA: 0.48 vs. 0.58, P < 0.001	High; FA higher than in previous similar studies, correlation with EDSS NR
Manconi et al. (2008); prospective, cross-sectional	MS (82 total, 30 with restless leg syndrome), no HCs	Same as Agosta et al. (2005)					Semi-automated segmentation, manual ROI in mid-sagittal slice from C1–C5	• EDSS • Qualitative RLS and sleep data	• Mean FA decreased in RLS subjects vs. non-RLS (P = 0.02) • FA histogram peak higher in RLS (P = 0.004) • No correlations between spinal cord DTI metrics and brain DTI or number of cord lesions (on STIR)	High; coarse clinical data, single mid-sagittal slice misses key WM tracts
Shanmuganathan et al. (2008); retrospective, cross-sectional	aSCI (20 total, 16 with neurological injury) vs. HCs (8)	1.5 T; Siemens; 12-channel head/neck PAC	• Medulla-T1 • 67 axial slices, contiguous	• ssEPI • Partial Fourier • 6 direcctions • b = 1000 s/mm^2^	200 mm^2^; 128 × 128; 1.6 × 1.6 × 3 mm^3^; 8000/76; no; 3 min 40 s	FA, MD, RA, VR, λ_1_, λ_2_, λ_3_	Manual, 3 ROIs drawn to include GM and WM, medulla-C2, C3–C5, and C6–T1	• None	• Decreased MD vs. HCs in all 3 ROIs: P ≤ 0.01 • Decreased λ_1_ vs. HCs in all 3 ROIs: P ≤ 0.002	High; retrospective, 4/20 subjects excluded due to image quality, no clinical data
Agosta et al. (2009a); prospective, longitudinal	ALS (17) vs. HCs (20)	Same as Valsasina et al. (2007)						• ALSFRS • FU at 6–12 months (mean 9)	• At FU, FA decreased: 0.45 vs. 0.48, P = 0.01 • At FU, MD increased: 0.95 vs. 0.89, P = 0.01 • FA, MD changes did not correlate with ALSFRS changes	High; only 61% had FU MRI, prediction of FU EDSS NR
Agosta et al. (2009b); prospective, cross-sectional	PPMS (23) vs. HCs (18)	Same as Agosta et al. (2008b)						• EDSS	• Decreased FA: 0.45 vs. 0.57, P < 0.001 • Increased MD: 0.99 vs. 0.85, P < 0.001 • FA correlates with mean cord fMRI signal change: r = − 0.58	High; coarse clinical data, correlation with EDSS NR
Cruz et al. (2009); retrospective, cross-sectional	RRMS (41) vs. HCs (37)	1.5 T; Siemens; 8 channel head coil; NR	• C2–C3 • Axial slices: 30% gap; sagittal slices: contiguous, number NR	• DTI sequence NR • 12 directions • b value NR	Axial: 225 mm^2^; 128 × 128; 1.8 × 1.8 × 3 mm^3^; 3200/80; no; AT NR; sagittal: 280 mm^2^ 192 × 192; 1.5 × 1.5 × 3 mm^3^, 2800/90; no; NR	FA	Manual, on plaque, peri-plaque, NASC, vs. whole-cord (HCs)	• None	• FA in plaques (0.44) is lower than periplaque (0.57), NASC (0.63), or HCs (0.74): P < 0.001 • FA lower in NASC vs. controls: P < 0.05	High; retrospective, no clinical data
van Hecke et al. (2009); prospective, cross-sectional	MS (21) vs. HCs (21)	1.5 T; Siemens; spine, neck coils; 40 mT/m	• C1–C5 • 30 axial slices, contiguous	• ssEPI • Parallel (factor NR) • 60 directions • b = 700 s/mm^2^	256 mm^2^; 128 × 128; 1.4 × 1.4 × 3 mm^3^; 10,400/100; no; 12 min 18 s	FA, MD, AD, RD, ψ (from FT)	Manual, whole cord	• None	• Decreased FA, ψ in MS with lesions (P < 0.01) and without (P < 0.02)	High; no clinical data, diagnostic accuracy NR
Benedetti et al. (2010); prospective, cross-sectional	MS (68 total, 40 BMS, 28 SPMS) vs. HCs (18)	Same as Agosta et al. (2005)						• EDSS	• Total MS: increased MD (P = 0.001), decreased FA: (P < 0.001) • SPMS: lower mean cord FA than BMS: 0.33 vs. 0.37, P = 0.01 • Mean FA correlates with EDSS: r = − 0.37, P = 0.002 • Multivariate model (brain, cord) correlates with EDSS: r = 0.58	High (diagnostic), moderately high (correlation); coarse clinical data
Freund et al. (2010); prospective, longitudinal	MS with acute lesion (14) vs. HCs (13)	1.5 T; GE; NR; 33 mT/m	• C1–C5 • 30 axial slices, contiguous	• CO-ZOOM-EPI rFOV • 60 directions • b = 1000 s/mm^2^	70 × 47 mm^2^; 48 × 32; 1.5 × 1.5 × 5 mm^3^; 15 beats/96; yes; NR	FA, MD, AD, RD, FU MRI at 1 min, 3 min, 6 min	Manual, 4 ROIs in ACs, DCs, L/R LCs	• EDSS • 9 hole peg • 25-foot TWT • MSWS-12 • FU at 1 min, 3 min, 6 min	• FA decreased and RD increased vs. HCs in all ROIs (P < 0.05) • Baseline RD predicted EDSS, 9 hole peg, and TWT at 6 min (P < 0.05) • Baseline FA of the LCs predicted EDSS recovery at 6 min (P = 0.02)	Moderately high; several datasets excluded due to artifact
Nair et al. (2010); prospective, cross-sectional	ALS (14) vs. HCs (15)	3 T; Siemens; 12-channel head and 2-channel neck PACs	• C1–C6 • 19 coronal slices, contiguous	• ssEPI • NEX = 2 • 2 acquisitions • 30 directions • b = 1000 s/mm^2^	160 mm^2^; 128 × 128; 1.3 × 1.3 × 2.5 mm^3^; 3200/105; no; 7 min (for 2 acquisitions)	FA, MD, AD, RD	Semi-automatic, FA skeleton used to define WM	• ALSFRS-R • FVC • Finger/foot tapping speed	• FA decreased (P = 0.003), RD increased (P = 0.03) • Multiple correlations: FA with tapping: r = 0.61, P = 0.02; RD with ALSFRS-R (r = − 0.55, P = 0.04), FVC (r = − 0.69, P = 0.01), and tapping (r = − 0.59, P = 0.03); MD with ALSFRS-R (r = − 0.56, P = 0.04) and FVC (r = − 0.54, P = 0.01)	High (diagnostic), moderately high (correlation); complex analysis likely requires expert
Xiangshui et al. (2010); prospective, cross-sectional	CSM (84) vs. HCs (21)	3 T; GE; neck PAC; 40 mT/m	• C1–C7 • 28 axial slices, contiguous	• SENSE EPI • 15 directions • b = 1000 s/mm^2^	270 mm^2^; 96 × 96; 2.8 × 2.8 × 4 mm^3^; 6000/83; no; 5 min	FA, MD, λ_1_, λ_2_, λ_3_	Manual, whole-cord	• None	• CSM divided into groups A–D by T2w changes • All metrics altered vs. HCs in groups B–D (P < 0.01) • Only λ_2_, λ_3_ differed between group A and HCs (P < 0.05)	High, no clinical data, large voxels
Cheran et al. (2011); prospective, longitudinal	aSCI (25 total, 13 HC, 12 NHC) vs. HCs (11)	1.5 T; Siemens; 12-channel head/neck PAC	• Caudal medulla and C1–T1 • 67 axial slices, contiguous	• ssEPI, partial Fourier, GRAPPA = 2 • 6 directions • b = 1000 s/mm^2^	200 mm^2^; 128 × 128; 1.6 × 1.6 × 3 mm^3^; 8000/76; no; 3 min 40 s	FA, MD, AD, RD	Manual, mid-sagittal slice: C1–C2, C3–C5, C6–T1, avoiding hemorrhage	ASIA motor score FU data in 12 subjects (at 1–29 months)	FA reduced at C3–C5, C6–T1 (NHC: P < 0.001, HC: P < 0.05) and at injury site (P < 0.001) MD, AD reduced in all regions (P < 0.001) All metrics correlated with motor score in NHC (R = 0.78–0.92)	High (diagnostic), moderately high (correlation); 7 subjects excluded, ROI misses key WM, prediction of outcomes NR
Cohen-Adad et al. (2011); prospective, cross-sectional	cSCI (14) vs. HCs (14)	3 T; Siemens; head/neck/spine PACs; NR	• C2–T2 • 8 axial slices, mid-VB (gap adjusted to fit)	• ssEPI, GRAPPA = 2 • 2 sat bands • Repeated 4 × • Manual shim • 64 directions • b = 1000 s/mm^2^	128 mm^2^; 128 × 128; 1 × 1 × 5 mm^3^; 1 heartbeat/76; yes (delay NR); NR	FA, MD, AD, RD, GFA	Manual, 4 ROIs: ACs, DCs, L/R LCSTs; lesion levels skipped	• ASIA motor and sensory scores	• Decreased FA,GFA (P < 0.0001) and AD, RD (P = 0.01) • FA, GFA, RD correlate with total ASIA (abs r = 0.66–0.74, P < 0.01) • Tract-specific metrics: weak specificity with motor vs. sensory scores	High (diagnostic), moderately high (correlation); manual ROI
Kamble et al. (2011); prospective, cross-sectional	cSCI (18) vs. HCs (11)	1.5 T; GE; spine coil; NR	• Cervical or lumbar • Axial slices, contiguous, number NR	• EPI • 25 directions • b = 1000 s/mm^2^	260 mm^2^; 128 × 128; 2 × 2 × 5 mm^3^; 8500/98; no; NR	FA	Manual, 3 ROIs placed randomly	• None	• FA in areas above/below lesion decreased vs. HCs: 0.37 vs. 0.55, P = 0.001	High; no clinical data, random ROI placement could miss key WM
Lee et al. (2011); prospective, longitudinal	CM (20) vs. HCs (20)	3 T; Philips; head/neck PAC; 40 mT/m	• C1–T1 • Sagittal slices, number, gap NR	• ssEPI, SENSE = 2 • NEX = 4 • 15 directions • b = 600 s/mm^2^	250 × 224 mm^2^; 128 × 128; 2 × 2 × 2 mm^3^; 3380/56; no; 3 min 43 s	FA, MD	Manual, whole-cord	• JOA • FU JOA at 3 months	• FA decreased at MCL: 0.50 vs. 0.60, P = 0.001 • MD increased at MCL: 1.44 vs. 1.17, P = 0.001 • FA, MD not correlated with JOA and not predictive of outcome	High; heterogeneous subjects, correlation coefficients not calculated
Mueller-Mang et al. (2011); prospective, cross-sectional	HIV (20) vs. HCs (20)	3 T; Siemens; standard neck coil; NR	• C2–C3 • 10 axial slices, contiguous	• SE double shot EPI, parallel = 2 • 6 directions • b = 1000 s/mm^2^	180 mm^2^; 256 × 256; 0.7 × 0.7 × 3 mm^3^; 3700/98; no; 2 min	FA, MD, λ_1_, λ_2_, λ_3_	Manual, 7 ROIs at C2–3: central GM, L/R ACs, DCs, LCSTs	• None	• No difference in metrics between HIV and HCs	High; negative study results, small voxels likely have very low SNR
Song et al. (2011); prospective, cross-sectional	CSM (53) vs. HCs (20)	1.5 T; Philips; spine PAC; 23 mT/m, 150 mT/m/ms	• C2–C6 • Sagittal slices, contiguous, number NR	• ssEPI • NEX = 4 • 6 directions • b = 400 s/mm^2^	230 mm^2^; 128 × 128; 1.8 × 1.8 × 3 mm^3^; NR; no	FA, MD	Manual, ROIs drawn at MCL (CSM), at disk levels (HCs)	• None	• FA decreases at descending cervical levels: P < 0.01 • MD increased (837 vs. 733, P < 0.01) and FA decreased (736 vs. 776, P < 0.01)	High; no clinical data, patients followed for 6 months but outcomes NR
Hori et al. (2012); prospective, cross-sectional	CSM (50 total, 18 with cord compression), no HCs	3 T; Philips; NR; NR	• C3–C6 • 30 axial slices, contiguous	• Sequence NR • 6 directions • b = 400, 800, 1200, 1600, 2000 s/mm^2^	80 mm^2^; 64 × 64; 1.3 × 1.3 × 3 mm^3^; 6996/73; no; 7 min	FA, MD, MK, RMSD	Manual, whole-cord at C3–4, C4–5, C5–6	• None	• Compressed cords (N = 18) had lower FA (0.61 vs. 0.66, P = 0.006), lower MK (0.80 vs. 0.91, P = 0.002), and higher RMSD (8.4 vs. 8.3, P = 0.006)	High; 15/50 subjects excluded due to artifacts, no clinical data, no HCs
Jeantroux et al. (2012); prospective, cross-sectional	NMO (25) vs. HCs (20)	1.5 T; Siemens; head, spine PACs; NR	• C1–C7 • 30 axial slices, contiguous	• SE EPI • 12 directions • b = 800 s/mm^2^	230 mm^2^; 104 × 104; 2.2 × 2.2 × 5 mm^3^; 2700/71; no; 7 min	FA, MD	Manual, NAWM and intralesional (based on T2)	• None	• Decreased FA in lesions (0.48, P < 0.001) and NAWM (0.58, P < 0.05) vs. HCs (0.61) • Increased MD in lesions (1.29, P < 0.001) and NAWM (1.11, P < 0.05) vs. HCs (1.03)	High; no clinical data, large voxels
Kerkovsky et al. (2012); prospective, cross-sectional	CSM (52 total, 20 with myelopathy) vs. HCs (13)	1.5 T; Philips; 16-channel head/neck PAC; NR	• Axial slices (number, gap NR)	• ssEPI, SENSE = 2 • 15 directions • FA = 25° • b = 900 s/mm^2^	NR; NR; 4 mm thick; 3549/83; no; NR	FA, MD	Manual, whole-cord at C2–3 and max. compression	• SSEPs • MEPs	• FA decreased at MCL in myelopathic subgroup (P = 0.001) and non-myelopathic subgroup (P = 0.04) • No difference in FA, MD at C2–3 between groups • EP measures only 67% sensitive in myelopathy	High; no clinical data (only EP), MRI details NR
Lindberg et al. (2012); prospective, cross-sectional	CSM (15) vs. HCs (10)	1.5 T; Siemens; NR; NR	• C2–C7 • 12 sagittal slices, contiguous	• ssEPI, SENSE = 2 • NEX = 4 • 2 sat bands • 25 directions • b = 900 s/mm^2^	180 mm^2^; 128 × 128; 1.4 × 1.4 × 3 mm^3^; 2000/95; no; 4 min 26 s	FA, MD, AD, RD	Manual, whole-cord	• Presence/absence of gait change or hyperreflexia	• FA decreased (C2–C7): 0.50 vs. 0.54, P = 0.02 • RD increased (C2–C7): 0.56 vs. 0.52, P = 0.03 • FA decreased with descending vertebral level (P value NR)	High; minimal clinical data
Pessoa et al. (2012); prospective, cross-sectional	MS (32) vs. NMO (8) vs. HCs (17)	1.5 T; Siemens; 8-channel head and neck PACs; NR	• C2–C7 • 16 sagittal slices, 0.3 mm gap	• ssEPI • 20 directions • b = 400,800 s/mm^2^	260 mm^2^; 128 × 128; 2 × 2 × 3 mm^3^; 2800/88; no; NR	FA, MD, AD, RD	Manual, 4 ROIs at C2 and C7: ACs, DCs, and R/L LCs	• EDSS (NMO subjects only)	• FA decreased, RD increased (only in AC at C2) in NMO vs. MS (P < 0.05) and NMO vs. HC (P < 0.05) • In NMO, FA in DC at C2 correlates with EDSS (r = − 0.80, P = 0.02)	High; coarse clinical data (NMO only), large voxels
Petersen et al. (2012); prospective, cross-sectional	cSCI (19) vs. HCs (28)	3 T; Philips; 6-element spine coil; NR	• C2, C5, T5, T12 • 6 axial slices per region, gap NR	• ssEPI, partial Fourier • NEX = 12 • Directions NR • b = 750 s/mm^2^	120 × 30 mm^2^; 176 × 44; 0.7 × 0.7 × 5 mm^3^; 4000/49; no; 30 min (for 3 regions)	FA, MD	Manual, 5 ROIs: whole-cord, L/R LCSTs and DCs; slices with SNR < 20 excluded	• AIS • SSEPs • MEPs	• FA (C2) decreased in whole-cord, LCSTs, and DCs (P < 0.005) • FA (C2) correlates with AIS in each ROI: whole-cord (r = 0.64, P = 0.001), LCSTs (r = 0.50, P = 0.002), and DCs (r = 0.41, P = 0.01) • Mean FA of DCs correlates with tibial SSEP amplitude (r = 0.46, P < 0.001)	High (diagnostic), moderately high (correlation); coarse clinical data, long acquisition time
Rocca et al. (2012); prospective, cross-sectional	MS (35 total, 20 with fatigue, 15 without) vs. HCs (20)	Same as Agosta et al. (2008b);						• EDSS • Fatigue Severity Scale	• FA decreased, MD increased in all MS vs. HCs (P < 0.001) • No difference in FA, MD between MS groups • DTI metrics do not correlate with clinical measures	High (diagnostic), moderately high (correlation); no correlations found
Wang et al. (2012); prospective, cross-sectional	CM (42) vs. HCs (49)	3 T; Philips; CTL coil; 80 mT/m, 200 mT/m/s	• C1–C7 or T6–T12 • Sagittal slices, number NR, contiguous	• SE ssEPI • 6 directions • b = 700 s/mm^2^	170 × 136 mm^2^; 96 × 61; 1.6 × 1.9 × 2 mm^3^; 5000/64; no; 30 min (for 3 regions)	FA, MD	Manual, rectangular ROIs placed at MCL (in CM) or mid-disk levels in HCs	• None	• FA decreased, MD increased in CM with T2w-HI vs. HCs (P < 0.05) • Metrics not different in CM without T2w-HI vs. HCs	High; heterogeneous subjects, no clinical data
Cohen-Adad et al. (2013); prospective, cross-sectional	ALS (29) vs. HCs (21)	Same as Cohen-Adad et al. (2011)				FA, MD, AD, RD	Same as Cohen-Adad et al. (2011)	• ALSFRC-R • TMS motor threshold	• FA decreased in LCST: 0.51 vs. 0.60, P < 0.0005 • FA correlates with ALSFRC-R (R = 0.38, P = 0.04) and motor threshold (R = − 0.47, P = 0.02) • Reduction in FA greatest at caudal levels	High; manual ROI
Gao et al. (2013); prospective, cross-sectional	CSM (104), no HCs	3 T; GE; 8-channel head/neck PAC	• C2–C7 • 27 axial slices, contiguous	• ssEPI • 2 sat bands • High order shim • 15 directions • b = 1000 s/mm^2^	27 mm^2^; 96 × 96; 0.3 × 0.3 × 4 mm^3^; 6000/83; no; NR	FA, MD, λ_1_, λ_2_, λ_3_	Manual, 3 regions of 10 voxels per slice	• JOA	• FA, MD, λ_2_, λ_3_ differ between JOA severity groups: P < 0.001 • FA, MD, λ_2_, λ_3_ differ with T1w/T2w signal change • FA correlates with JOA: r = 0.88, P < 0.05	High; no HCs, small voxels with low SNR, small FOV likely to have aliasing
Jones et al. (2013); prospective, longitudinal	CSM (30), no HCs	3 T; GE; cervical spine coil	• C2–T1 • 24 axial slices, contiguous	• ssEPI • 6 directions • b = 1000 s/mm^2^	180 mm^2^; 128 × 128; 1.4 × 1.4 × 4 mm^3^; 8100/94; no; 3 min 55 s	FA	Manual, 3 ROIs: DCs, L/R LCs at C2–3, MCL, C7–T1	• mJOA, Nurick, NDI, SF-36 • FU at 2–12 months (N = 15)	• FA correlates with mJOA (r = 0.62, P < 0.01) and Nurick (r = − 0.46, P = 0.01) • Higher FA predicts post-op improvement on NDI (r = − 0.61, P = 0.04)	Moderately high (correlation), high (prognostic); short FU times, multiple comparisons not corrected
Koskinen et al. (2013); prospective, cross-sectional	cSCI (28 total, 13 with surgical fixation hardware) vs. HCs (40)	3 T; Siemens; 12-channel head and 4-channel neck PACs; NR	• C2–C6 • Axial slices, number NR, 1.2 mm gap	• EPI • 20 directions • NEX = 4 • b = 1000 s/mm^2^	152 mm^2^; 128 × 128; 1.2 × 1.2 × 4 mm^3^; 4000/103; no; 5 min 50 s	FA, MD, AD, RD	Manual, whole-cord at C2–3, lesion (rostral edge), and C3–4, C4–5, C5–6 (HCs)	• ASIA motor and sensory scores • FIM	• Decreased FA at C2–3: 0.58 vs. 0.69, P < 0.001 • Increased MD and RD at C2–3: P < 0.001 • FA, MD significantly altered at lesion level (P < 0.001) • FA at lesion correlates with ASIA motor: r = 0.67, P < 0.01	High (diagnostic), moderately high (correlation); subjects not age-matched with HCs, 6 subjects excluded
Miraldi et al. (2013); prospective, cross-sectional	RRMS (32) vs. HCs (17)	1.5 T; Siemens; 8-channel head/neck PAC; NR	• C2–C7 • 16 axial slices, 0.3 mm gap	• ssEPI • 20 directions • b = 800 s/mm^2^	260 mm^2^; 128 × 128; 2 × 2 × 3 mm^3^; 2800/88; no; 15 min	FA, MD, AD, RD (from FT)	Manual, 4 ROIs in ACs, DCs, L/R LCs, at C2 and C7	• EDSS	• Most metrics showed no difference with controls • No significant correlation with EDSS	High; negative results, high variance of metrics
Naismith et al. (2013); prospective, cross-sectional	Myelitis (37 total, 26 MS, 11 NMO) vs. HCs (15)	3 T; Siemens; 2 or 4-channel neck PAC; NR	• C1–2, C3–4, C5–6 • 6 axial slices/region, contiguous	• rFOV ssEPI • 25 directions • Repeated 4 × • Shim: field-map • b = 600 s/mm^2^	72 × 29 mm^2^; 80 × 32; 0.9 × 0.9 × 5 mm^3^; 5 beats/99; yes; 45 min (4 acquisitions)	FA, MD, AD, RD	Manual, whole-cord and L/R DCs and LCSTs drawn on each slice	• EDSS • Vibration threshold • 25-foot TWT • 9 hole peg	• FA, RD of DCs (but not LCSTs) correlate with vibration (P < 0.01) • FA, RD of DCs and LCSTs correlate with 9 hole peg (all P < 0.0001) • FA, RD of whole cord (or tracts) correlate with EDSS categories (P < 0.0001)	High; heterogeneous subjects, 4 subjects and 33% of ROIs excluded due to artifacts/SNR
Oh et al. (2013a); prospective, cross-sectional	MS (124 total, 69 RRMS, 36 SPMS, 19 PPMS), no HCs	3 T; Philips; 2 element surface PAC;	• C2–C6 • 30 axial slices, contiguous	• Multi-slice SE ssEPI, parallel = 2 • 16 directions • b = 500 s/mm^2^	NR; NR; 1.5 × 1.5 × 3 mm^3^; 4727/63; no; NR	FA, MD, AD, RD	Automatic segmentation, whole-cord at C3–4 (11 slices)	• EDSS • MSFC	• FA, MD, AD, RD more abnormal with high vs. low EDSS in low or high lesion count subjects (all P < 0.05 except AD in high lesion count)	Moderately high; convenience sampling enrollment
Oh et al. (2013b); prospective, cross-sectional	MS (129 total, 74 RRMS, 36 SPMS, 19 PPMS) vs. HCs (14)	Same as Oh et al. (2013a)						• EDSS • Hip flexion power • Vibration	• FA, MD, AD, RD differed vs. HCs (P < 0.05) • FA, MD, RD differed from progressive MS vs. RRMS (P < 0.05) • FA, MD, RD correlate with EDSS (P < 0.05) • FA, RD correlate with vibration (P < 0.05) • MD, AD, RD correlate with hip flexion power (P < 0.05)	Moderately high (diagnostic), moderately low (correlation); diagnostic accuracy NR
Raz et al. (2013); prospective, cross-sectional	RRMS (19) vs. HCs (16)	3 T; Siemens; 4-channel neck PAC	• C1–C4 • 20 axial slices, contiguous	• SE (twice-refocused) EPI • NEX = 2 • 30 directions • b = 500, 1000, 1500, 2000, 2500 s/mm^2^	160 mm^2^; 128 × 128; 1.3 × 1.3 × 3 mm^3^; 3100/110; no; 15 min 7 s	FA, MD, MK	Manual, whole-cord from C1–C4, and NAGM, NAWM (DCs) at C2	• EDSS • Disease duration	• WM at C2: decreased FA vs. HCs: 0.52 vs. 0.62, P = 0.01 • GM at C2: decreased MK vs. HCs: 1.11 vs. 1.16, P = 0.01 • Lesions: decreased FA, MK, increased MD vs. NASC (P < 0.0001) • Metrics in whole-cord and GM (but not WM) differ between high EDSS vs. low (P ≤ 0.01) • No correlation between FA, MD, MK and EDSS	Moderately high (diagnostic), moderately low (correlation); no correlations found
Uda et al. (2013); prospective, cross-sectional	CSM (26) vs. HCs (30)	3 T; Philips; 16-element PAC; NR	• C2–T1 • 30 axial slices, contiguous	• SS FSE • NEX = 1 • 15 directions • b = 1000 s/mm^2^	240 mm^2^; 160 × 160; 1.5 × 1.5 × 3 mm^3^; 8000/80; no; 4 min 54 s	FA, MD, z-statistics calculated per level	Manual, whole-cord at disks, C2–T1	• None	• FA varied with cervical level (P < 0.0001) but increased at C7–T1 • MD had ROC AUC = 0.90, with SE = 100%, SP = 75%, PPV = 90%, and NPV = 100% • FA had ROC AUC = 0.76, with SE = 95%, SP = 50%	High; groups not age-matched, no clinical data
Von Meyenburg et al. (2013); prospective, cross-sectional	MS (38 total, 15 RRMS, 13 SPMS, 10 PPMS), 28 HCs	3 T; Philips; 6-element spine PAC	• C5 • 6 axial slices, contiguous	• rFOV ssEPI • Partial Fourier = 0.6 • 6 directions • b = 750 s/mm^2^	120 × 30 mm^2^; 176 × 44; 0.7 × 0.7 × 5 mm^3^; 4000/49; no; 10 min	FA, MD	Manual, 4 ROIs: L/R LCs and DCs	• EDSS • MEPs	• Decreased FA in all ROIs (all P ≤ 0.001) • No differences in MD • FA correlates with age (P < 0.05) • Tract-specific FA correlates with corresponding MEPs: r = − 0.93–0.94, P < 0.01	High; groups not age-matched, correlation with EDSS NR
Banaszek et al. (2014); prospective, cross-sectional	CSM (132) vs. HCs (25)	1.5 T; GE; 16-channel head/spine PAC; 33 mT/m	• C2–C7 • Axial slices, variable number, contiguous	• SE ssEPI • 2 acquisitions • 14 directions • b = 1000 s/mm^2^	160 mm^2^; 96 × 96; 1.6 × 1.6 × 4 mm^3^; 10,000/99; no; 5–7 min	FA, MD	Manual, whole-cord; images divided into 5 groups based on cord compression	• None	• FA decreased at all levels (C2–C6) vs. HCs (P < 0.0001) • FA correlated with measures of cord compression (P < 0.01) • MD increased in most levels/subgroups vs. HCs (P < 0.05)	High; no clinical data, images at C6–7 excluded due to artifacts
El Mendili et al. (2014); prospective, longitudinal	ALS (29), no HCs	3 T; Siemens; neck/spine coil; NR	• C2–T2 • 8 axial slices, mid-VB, variable gap	• ssEPI, GRAPPA = 2 • Repeated 4 × • 64 directions • b = 1000 s/mm^2^	128 mm^2^; 128 × 128; 1 × 1 × 5 mm^3^; 700/60; yes; 15 min	FA, MD, AD, RD; FU MRI at 1 year	Manual, 4 ROIs: ACs, DCs, L/R LCSTs	• ALSFRS-R • Muscle power • FU at 1 year	• FA of LCSTs correlates with ALSFRS-R leg (P < 0.001) and total (P = 0.04) scores • Baseline FA predicts ALSFRS-R leg (P = 0.002) and total (P = 0.001) scores at 1 year FU • No change in DTI metrics at 1 year FU vs. baseline	Moderately high; manual ROI
Ellingson et al. (2014); prospective, cross-sectional	CSM (48 total, 16 mJOA = 18) vs. HCs (9)	3 T; Siemens; CTL spine PAC (2 elements); NR	• Upper cervical cord (HCs) • MCL (CSM) • Axial slices, number NR	• rFOV ZOOMED-EPI • 6 directions • NEX = 15 • b = 500 s/mm^2^	NR; NR; NR; 5000/67; no; NR	FA, MD, AD, RD, ψ, SD(θ)	Manual, whole-cord at MCL or upper cord (HCs)	• mJOA	• FA diagnostic of mJOA < 18 vs. mJOA = 18 with SE = 72%, SP = 75% (AUC = 0.77) • FA diagnostic of mJOA < 15 with SE = 81%, SP = 92% (AUC = 0.95) • FA correlates with mJOA: R^2^ = 0.41, P < 0.0001 • SD(θ) correlates with mJOA: R^2^ = 0.41, P < 0.0001	High; MRI details NR, age/gender of HCs NR, metrics at MCL potentially biased
Li et al. (2014); prospective, cross-sectional	CSM (14) vs. HCs (14)	3 T; Philips; NR; NR	• C3–C7 • Axial slices, number/gap NR	• SE EPI • 15 directions • b = 600 s/mm^2^	NR; NR; 1 × 1.3 × 7 mm^3^; 5 beats/60; yes; NR	OE, wOE	Manual, whole-cord	• Muscle power • Reflexes • Sensory testing	• Diagnosis of symptomatic level with OE had SE = 81%, SP = 67%, wOE had SE = 81%, SP = 100%	High; groups not age-matched, OE not compared with standard metrics
Rajasekaran et al. (2014); prospective, cross-sectional	CSM (35) vs. HCs (40)	1.5 T; Siemens; NR; NR	• C1–T1 • 40 axial slices, gap NR	• SE ssEPI • 12 directions • b = 500 s/mm^2^	220 mm^2^; 256 × 256; 0.9 × 0.9 × 4 mm^3^; 6000/85; no; NR	FA, MD, λ_1_, λ_2_, λ_3_	Manual, whole-cord, at C1 and disks: C2–T1	• Nurick	• All metrics differed between CSM vs. HCs at MCL: P < 0.01 • DTI metrics not different between high and low Nurick grades • No correlation between DTI metrics and Nurick grades	High; coarse clinical data, comparison vs. HCs not at same level (C1–T1) as MCL
Toosy et al. (2014); prospective, cross-sectional	MS (14) vs. HCs (11)	1.5 T; GE; NR; 33 mT/m	• C1–C5 • 30 axial slices, contiguous	• CO-ZOOM-EPI rFOV • 60 directions • b = 1000 s/mm^2^	70 × 47 mm^2^; 48 × 32; 1.5 × 1.5 × 5 mm^3^; 15 beats/96; yes; NR	FA, MD, AD, RD	Automatic (registered to template), whole-cord and lesions using TFCE, P < 0.01	• EDSS • 9 hole peg • 25-foot TWT • MSWS	• FA decreased, RD increased (P < 0.01) • FA correlates with EDSS (R = − 0.6, P = 0.05) and TWT (R = 0.7, P = 0.02) • RD correlates with EDSS (R = 0.7, P = 0.01) and TWT (R = − 0.6, P = 0.05)	High (diagnostic), moderately high (correlation); 4 subjects excluded (image processing)
Wang et al. (2014); prospective, cross-sectional	ALS (24) vs. HCs (16)	1.5 T; GE; 8-channel spine coil; NR	• C2–C4 • 24 axial slices, contiguous	• SE ssEPI, NEX = 4 • 6 directions • b = 400 s/mm^2^	2240 mm^2^; 128 × 128; 1.9 × 1.9 × 4 mm^3^; 6000/min; no; NR	FA, MD	Manual, 5 ROIs: DCs, L/R STs, LCSTs at mid-VB C2–C4	• ALSFRS-R • mNorris • EMG	• FA decreased in LCSTs at all levels (P < 0.01), not DCs, STs • MD increased in LCSTs at all levels (P < 0.05), not DCs, STs • DTI metrics not correlated with clinical measures	High; large voxels (difficult to assess individual tracts), manual ROI
Wen et al. (2014a); prospective, cross-sectional	CSM (15) vs. HCs (25)	3 T; Philips; head/neck PAC; NR	• C1–C7 • 12 axial slices mid-VB or mid-disk	• ssEPI with spatial presaturation • 15 directions • b = 600 s/mm^2^	880 mm^2^; 80 × 64; 1 × 1.3 × 4 mm^3^; 5 beats/60; yes; 24 min	FA, MD, AD, RD	Manual, ACs, LCs, DCs at MCL	• mJOA • SSEPs	• FA in HCs higher in DCs and LCs than ACs (P < 0.05) • FA decreased selectively in LCs and DCs at MCL, but not in ACs (P < 0.05)	High; groups not age-matched, only severe CSM subjects included
Wen et al. (2014b); prospective, longitudinal	CSM (45) vs. HCs (20)	Same as Wen et al. (2014a)					Manual, whole-cord	• mJOA • SSEPs • Recovery ratio (6 min–2 years FU)	• Reduced mean FA: 0.65 vs. 0.52, P < 0.001 • FA correlates with mJOA: R^2^ = 0.33, P = 0.02 • FA predicts good mJOA recovery ratio: P = 0.03	High; groups not age-matched, coarse clinical data, 2 inconsistent definitions of mJOA recovery rate
Zhou et al. (2014); prospective, cross-sectional	CSM (19) vs. HCs (19)	3 T; Siemens; NR; NR	• C1–C7 • 16 axial slices, gap NR	• SE ssEPI, NEX = 2 • 20 directions • b = 600 s/mm^2^	8128 × 124 mm^2^; 128 × 124; 1 × 1 × 5 mm^3^; 5000/106; yes; 24 min	FA	Manual, whole-cord at C2, MCL	• JOA	• FA decreased at C2 (0.60 vs. 0.67, P = 0.01) and MCL (0.51 vs. 0.66, P < 0.001) • Amplitude of right pre-central and post-central gyri oscillations correlate weakly with FA at C2 (P < 0.05)	High; primarily brain fMRI study, with cord DTI as secondary measure
Abbas et al. (2015); prospective, cross-sectional	Pott Disease (30 total, 15 with paraplegia, 15 without), no HCs	3 T; Siemens; NR; NR	• 1 VB above to 1 VB below lesion • 25 axial slices, 2 mm gap	• SPAIR, NEX = 4 • 20 directions • b = 700 s/mm^2^	1280 mm^2^; 128 × 128; 2.2 × 2.2 × 5 mm^3^; 4100/66; no; NR	FA, MD	Manual, central GM/WM at 3 levels: 1 VB above, at lesion, and 1 VB below	• Jain and Sinha score • Presence of paraplegia	• FA higher above vs. below lesion in all subjects (P < 0.05) • No difference between metrics with or without paraplegia	High; non-standard/coarse clinical data, large voxels
Iglesias et al. (2015); prospective, cross-sectional	ALS (21) vs. HCs (21)	3 T; Siemens; neck/spine coil; NR	• C2–T2 • 8 axial slices, mid-VB, variable gap	• ssEPI, GRAPPA = 2 • Repeated 2 × • 64 directions • b = 1000 s/mm^2^	1128 mm^2^; 128 × 128; 1 × 1 × 5 mm^3^; 700/60; yes; 10 min	FA, MD, AD, RD	Manual, 4 ROIs: ACs, DCs, L/R LCSTs	• SSEPs • ALSFRS-R • 9 hole peg • Muscle power	• 58% of ALS group had abnormal MD, RD values (outside 95% CI) in DCs • DTI metrics only correlated with N9 amplitude, not N20 • DTI metrics not correlated with clinical measures	High; 3 subjects excluded due to artifacts, no correlation with clinical scores
Maki et al. (2015); prospective, cross-sectional	CSM (20) vs. HCs (10)	3 T; GE; 8 channel neck PAC; NR	• C1–T1 • 15 axial slices, mid-VB/mid-disk, variable gap	• rFOV SE ssEPI, NEX = 16 • 6 directions • b = 700 s/mm^2^	140 × 30 mm^2^; 176 × 44; 0.7 × 0.7 × 5 mm^3^; 3000/75; no; NR	FA	Manual, 2 ROIs: DCs, LCs one slice above MCL	• JOA	• FA decreased in LCs (0.59 vs. 0.71, P = 0.01) and DCs (0.58 vs. 0.72, P < 0.01) but ranges overlap • FA correlates with JOA: r = 0.48, P = 0.03 for both LCs, DCs • FA correlates with JOA lower extremity subscore in LCs (r = 0.76, P < 0.01) and DCs (r = 0.74, P < 0.01) • ICC for ROI selection: 0.72–0.80	High; groups not age-matched, manual tract-specific ROIs had only moderate reliability
Oh et al. (2015); prospective, cross-sectional	MS (102 total, 66 RRMS, 24 SPMS, 12 PPMS) vs. HCs (11)	Same as Oh et al. (2013a)				FA, RD	Same as Oh et al. (2013a)	• EDSS • MSFC • Vibration • Hip flexion • OCT retinal measures	• RD (but not FA) decreased in progressive MS vs. RRMS (P = 0.03) • FA, RD correlate with several measures of retinal layers (P < 0.01) • DTI metrics do not independently correlate with clinical measures in multivariate regression	Moderately high (diagnostic), moderately low (correlation); no correlation found
Vedantam et al. (2015); retrospective, cross-sectional	aSCI (12) vs. HCs (12)	1.5 T; GE; CTL spine coil; NR	• C1–T1	• Sequence NR • 15/25 directions (19/5 subjects) • b = 500/600 s/mm^2^	190 mm^2^; 128 × 128; 1.5 × 1.5 mm^2^ (thickness NR); 5000/98; no; NR	FA	Manual, whole-cord and LCSTs, C1–C2	• ASIA motor and sensory scores • AIS	• FA decreased at C1–2 in whole-cord (0.61 vs. 0.67, P < 0.01) and LCSTs (0.66 vs. 0.70, P = 0.04) • FA of LCSTs correlates with AIS (r = 0.71, P = 0.01), and upper limb motor score (r = 0.67, P = 0.01) • DTI metrics did not correlate with sensory scores	High; MR pulse sequence NR, manual ROIs
Yan et al. (2015); prospective, cross-sectional	Chiari I with Syringomyelia (23) vs. HCs (8)	1.5 T; Philips; 16-channel NC coil;	• C2–T1 • Axial slices, number/gap NR	• EPI • 15 directions • b = 400 s/mm^2^	224 mm^2^; 112 × 109; 2 × 2 × 2 mm^3^; 2170/59; no; 10 min	FA	Manual, whole-cord at syrinx and above/below	• None	• No difference in FA above/below syrinx vs. HCs • FA at syrinx decreased vs. HCs: 0.43 vs. 0.53, P < 0.05 • FA decreased at syrinx in symptomatic patients vs. asymptomatic: 0.37 vs. 0.45, P < 0.05	High; large voxels (and thinly stretched cord), definition of symptomatic NR

**Table 4:** Summary of DTI fiber tractography (FT) studies.

Authors (year); design	Subjects	B_0_; vendor; coil; gradients	Anatomical region/position	DTI acquisition	FOV; matrix; voxel size; TR/TE (ms); cardiac gating; AT	FT metrics	FT method; ROI	Clinical measures	Key results	Risk of bias; key barriers to translation
Facon et al. (2005); prospective, cross-sectional	See Table 3					None	Vector-based tracing; none	See Table 3	• FT only used in 3 subjects to assist with ROI	High; Detailed FT method NR, no quantitative analysis using FT
Renoux et al. (2006); prospective, cross-sectional	See Table 3					None	DPTools using FA > 0.17, angle < 45°; none	See Table 3	• Areas of myelitis with T2 hyper-intensity (and low FA) tended to show ‘spreading fibers’ or ‘broken fibers’ • FT had optimal results with b = 500 s/mm^2^	High; no quantitative analysis using FT
Ciccarelli et al. (2007); prospective, longitudinal	MS (14 acute, lesion at C1–C3) vs. HCs (13)	1.5 T; GE; NR; 33 mT/m	• C1–C7 • 30 axial slices, contiguous	• CO-ZOOM-EPI rFOV • 31 directions • b = 1000 s/mm^2^	70 × 47 mm^2^; 48 × 32; 1.5 × 1.5 × 5 mm^3^; 15 heartbeats/90; yes; AT NR	Connectivity index, FA, MD, AD, RD (from FT)	4 seed points (ACs, DCs, L/R LCSTs), FT with FA > 0.1; C1–C3 for each FT bundle	• EDSS • 9-hole peg • 25-foot TWT • MSWS-12 • FU: 3–6 min EDSS	• Decreased connectivity in LCSTs and DCs (P = 0.03) • Decreased FA in LCSTs (P = 0.006) and DCs (P = 0.02) • MD, AD, RD not different than HCs • Connectivity and FA of DCs correlates with 9-hole peg test (P < 0.05, r value NR)	High (diagnostic), moderately high (correlation); min FA, max angle NR, no prediction of EDSS
Hatem et al. (2009); prospective, cross-sectional	Syringomyelia (28) vs. HCs (19)	1.5 T; Siemens; NR; 40 mT/m	• C1–C7 • 12 sagittal slices, contiguous	• ssEPI • GRAPPA parallel factor = 2 • 25 directions • b = 1000 s/mm^2^	179 mm^2^; 128 × 128; 1.4 × 1.4 × 3 mm^3^; 2100/97; no; 4 min 37 s	FA, MD (from FT)	MedINRIA, with FA > 0.2; manual, 5 ROIs: whole-cord, L/R/A/P hemi-cords at C3–4, C6–7	• Thermal sensory tests • Laser EPs	• FA reduced in all ROIs: P < 0.05 • MD not different than HCs • FA at C3–4 (but not C6–7) correlates with thermal: r = − 0.63, P < 0.01	High; 9 subjects excluded due to artifacts, only sensory clinical data
van Hecke et al. (2009); prospective, cross-sectional	See Table 3					FA, MD, AD, RD, ψ (from FT)	Streamline-based FT, manual seed points, FA > 0.3, angle < 20°; whole-cord based on FT	• None	• FT segmentation had improved ICC vs. manual ROI: 0.96 vs. 0.79 (for FA) • Decreased FA, ψ in MS with lesions (P < 0.01) and without (P < 0.02)	High; no clinical data, diagnostic accuracy NR
Hatem et al. (2010); prospective, cross-sectional	Syringomyelia (37) vs. HCs (21)	Same as Hatem et al. (2009)				FA, MD (from FT), number of FT fibers	MedINRIA, with FA > 0.2; whole-cord based on FT, A/P hemi-cords	• Pain scores • Mechanical, vibration, thermal • Laser EPs • SSEPs	• FA (r = − 0.64, P = 0.02) and number of FT fibers (r = − 0.75, P = 0.02) correlate with average daily pain scores	High; correlation with sensory testing NR, only sensory clinical data
Xiangshui et al. (2010); prospective, cross-sectional	See Table 3					None	GE Functool, FA > 0.18, angle < 45°; none	• None	• Subjects with only dural indentation on T2w had normal FT • FT appeared distorted in subjects with cord compression on T2w	High; no quantitative analysis using FT
Budzik et al. (2011); prospective, cross-sectional	CSM (20) vs. HCs (15)	1.5 T; Philips; Sense spine coil; NR	• C1–C7 • 12 sagittal slices, contiguous	• ssEPI with SPIR, partial Fourier • 25 directions • b = 900 s/mm^2^	200 mm^2^; 128 × 128; 1.6 × 1.6 × 3 mm^3^; 2010/94; no; 3 min 33 s	FA, MD (from FT > 10 mm)	Semi-automated, no seed points; whole-cord based on FT at C2–3, MCL or C4–C7 (HCs)	• JOACMEQ	• FA decreased at compressed level vs. C4–C7 in HCs: 0.40 vs. 0.50, P = 0.0003 • FA at compressed level correlates with detailed UE (P < 0.001) and LE (P < 0.001) scores • FA negatively correlated with age: P = 0.04	High; FT parameters (min FA, max angle) NR
Lee et al. (2011); prospective, longitudinal	See Table 3					FT: intact, waist, partial, or broken	PRIDE, FA > 0.1, angle < 27°; whole-cord at MCL based on FT	See Table 3	• Tractography patterns not correlated with JOA	High; heterogeneous subjects, FT analysis uses subjective categories
Ukmar et al. (2012); prospective, cross-sectional	MS (27 total, 9 RRMS, 9 SPMS, 9 PPMS) vs. HCs (18)	1.5 T; Philips; NR; 33 mT/m, slew = 150 mT/m/s	• C1–C7, 40 axial slices, contiguous	• Sequence NR, fat sat, SENSE = 2 • 32 directions • b = 1000 s/mm^2^	224 mm^2^; 112 × 112; 2 × 2 × 2 mm^3^; 6731/91; no; 4 min 2 s	FA (manual ROI), FDI	DTI Studio, FA > 0.25, angle < 70°; manual, whole-cord, C1–C7	• EDSS	• No difference in FA vs. HCs • FDi decreased in MS: 12 vs. 16, P < 0.01 • No correlation of metrics with EDSS	High; large voxels, groups not age-matched, no correlation found
Wang et al. (2012); prospective, cross-sectional	See Table 3					FT: amount of compression	PRIDE, FA > 0.2; none	See Table 3	• FT normal in all 49 HCs • FT slightly compressed in 25/27 without T2w-HI • FT showed various degrees of severe compression in CM with T2w-HI	High; subjective analysis of FT, large voxels
Gao et al. (2013); prospective, cross-sectional	See Table 3					FT: deformed, thinning, or broken	NR; no ROI, qualitative impression of MCL	See Table 3	• FT deformed in 28/31 mild (JOA 13–16) subjects, thinning in 10/27 moderate (JOA 9–12) and 19/25 severe (JOA 5–8) subjects, broken in 18/21 serious (JOA 0–4)	High; DTT method NR, subjective FT categorization
Hodel et al. (2013); prospective, cross-sectional	Myelitis (40 total, 25 MS, 11 NMO, 4 other) vs. HCs (12)	3 T; Philips; 16-channel head/neck PAC; NR	• C1–C7 • 11 coronal slices	• rFOV ZOOMED-EPI, fat sat, partial Fourier, NEX = 3 • 15 directions • b = 600 s/mm^2^	42 × 170 mm^2^; 23 × 96; 1.8 × 1.8 × 2.5 mm^3^; 3 beats/39; yes; 7 min 30 s	FA, MD, AD, RD, Ψ (from FT)	Manual seed and termination points at C1, C7, using FMRIB; whole-cord based on FT	• EDSS • Pyramidal score • Sensory score	• FA and Ψ significantly decreased in overall cohort and all subgroups except MS with acute cervical lesions • Excluding active lesions, FA correlates with sensory score: r = − 0.4, P = 0.01	High; groups not age-matched, heterogeneous subjects, large voxels
Rajasekaran et al. (2014); prospective, cross-sectional	See Table 3					FT: intact, waist, partial, or broken	Method NR, manual seed points at C1–2, FA > 0.2; none	See Table 3	• FT results showed 4 waist, 21 partially broken, and 10 completely broken • No correlation between FT results and Nurick grade	High; FT method NR, no correlation found
Abbas et al. (2015); prospective, cross-sectional	See Table 3					None	Method NR; none	See Table 3	• 13/15 subjects without paraplegia had decreased FT thickness below lesion, and 14/15 had some disruption • 4/15 subjects with paraplegia had decreased FT thickness below lesion, 6/15 had some disruption, and 2/15 had complete cessation of FT	High; minimal clinical data, FT method NR, only qualitative assessment of FT
Cui et al. (2015); prospective, cross-sectional	CSM (23) vs. HCs (20)	3 T; Philips; head/neck coil; NR	• C1–C7 • 12 axial slices, gap NR	• rFOV SE ssEPI, fat sat • 15 directions • b = 600 s/mm^2^	80 × 36 mm^2^; 80 × 28; 1 × 1.3 × 7 mm^3^; 5 beats/60; yes; 24 min	FA, MD, AD, RD (from FT), FD	TrackVis, manual seed points at C2, angle < 35°; 7 ROIs from FT: whole-cord, L/R ACs, LCs, DCs	• JOA • Hand 10 second test	• Decreased FA in LCs, DCs: P < 0.001 • MD, AD, RD higher in all columns: P < 0.05 • Decreased FD: 0.29 vs. 0.32, P < 0.05	High; correlation with clinical measures NR

**Table 5:** Summary of MT studies.

Authors (year); design	Subjects	B_0_; vendor; coil	Anatomical region/position	MT acquisition	FOV; matrix; voxel size; TR/TE (ms); cardiac gating; AT	MT metrics	ROI	Clinical measures	Key results	Risk of bias; key barriers to translation
Silver et al. (1997); prospective, cross-sectional	MS (12 total, 8 RRMS, 4 SPMS) vs. HCs (12)	1.5 T; NR; neck PAC	• C1–C7 • 3 sagittal slices, contiguous	• FSE ± MT pre-pulse (sinc, 1 kHz offset, 20 ms, 1430°), NEX = 8	NR; 256 × 192; 5 mm thick; 1600/17; no; 17 min 40 s	MTR	Manual, ellipse drawn on mid-sagittal image from C1–C3	• EDSS	• Decreased MTR: 18 vs. 19, P = 0.0004 • No correlation between MTR and EDSS	High; no correlation with EDSS, mid-sagittal ROI misses key WM tracts
Bozzali et al. (1999); prospective, cross-sectional	MS (90) vs. HCs (20)	1.5 T; NR; tailored cervical PAC	• C1–C7 • 20 axial slices (contiguous) • 17 sagittal slices (0.3 mm gap)	• 2D GE ± MT pre-pulse (Gaussian, 1.5 kHz offset, 7.7 ms, 500°), NEX = 2, FA = 20°	Axial: 250 mm^2^; 192 × 256; 1 × 1 × 3 mm^3^; 640/10; no; NR; sagittal: 280 mm^2^; 224 × 256; 1 × 1 × 5 mm^3^; 640/10; no; NR	MTR, histogram peak, location	Manual, whole-cord	• EDSS	• Axial data more sensitive to pathology • Decreased MTR (axial): 44 vs. 46, P = 0.001 • Patients with EDSS ≥ 4.0 had lower MTR: P = 0.02	High; correlation coefficient not calculated
Filippi et al. (2000); prospective, cross-sectional	MS (96 total, 52 RRMS, 33 SPMS, 11 PPMS) vs. HCs (21)	1.5 T; Siemens; tailored cervical PAC	• C1–C7 • Slice orientation, number, gap NR	• 2D GE ± MT pre-pulse (Gaussian, 1.5 kHz offset, 7.7 ms, 500°), FA = 20°, NEX = 2	192 × 250 mm^2^; 256 × 256; 1 × 1 × 5 mm^3^; 640/12; no; NR	MTR, histogram peak, location	Semi-automatic, whole-cord, excluding voxels with MTR < 10%	• EDSS	• Decreased MTR in MS patients: 44% vs. 46% P = 0.006 • Peak location and height were independent predictors of EDSS ≥ 4.0 in multivariate analysis	High; correlation coefficient not calculated
Lycklama et al. (2000); prospective, cross-sectional	MS (65 total, 14 RRMS, 34 SPMS, 17 PPMS) vs. HCs (9)	1.0 T; Siemens; quadrature head coil	• Brain-C1 • 22 axial slices, 3 mm gap	• 2D GE ± MT pre-pulse (Gaussian, 1.5 kHz offset, 7.6 ms, 500°), FA = 30°, NEX = 2	NR; NR; 3 mm thick; 700/10; no; NR	MTR	Manual, whole-cord excluding edge voxels at C1	• EDSS	• Decreased MTR: 30 vs. 33, P < 0.01 • MTR correlates weakly with EDSS: r = − 0.25, P < 0.05	High; coarse clinical data, weak correlation with EDSS
Rovaris et al. (2000); prospective, cross-sectional	MS (77 total, 40 RRMS, 28 SPMS, 9 PPMS), no HCs	1.5 T; Siemens; tailored cervical PAC	• C1–C7 • 20 axial slices, contiguous	• 2D GE ± MT pre-pulse (Gaussian, 1.5 kHz offset, 7.7 ms, 500°), FA = 20°, NEX = 2	250 mm^2^; 192 × 256; 1 × 1 × 3 mm^3^; 640/10; no; NR	MTR, histogram peak, location	Semi-automatic, whole-cord, excluding voxels with MTR < 10%	• EDSS	• No difference in mean MTR, histogram height between RRMS, SPMS, and PPMS • Peak location significantly different for RRMS > SPMS > PPMS, P = 0.01 • Peak location corresponds with EDSS ≥ 3, P < 0.001	High; correlation coefficients not calculated
Inglese et al. (2001); prospective, cross-sectional	LHON (14) vs. HCs (20)	1.5 T; NR; standard cervical coil; NR	• C1–C4 • 20 axial slices, 0.3 mm gap	• 2D GE ± MT pre-pulse (Gaussian, 1.5 kHz offset, 16 ms, 850°), FA = 20°	250 mm^2^; 256 × 256; 1 × 1 × 5 mm^3^; 640/10; no; NR	MTR, histogram peak, location	Manual, whole-cord	• None	• No significant differences in MTR or histogram metrics vs. HCs	High; no group differences found, no clinical data
Rocca et al. (2001); prospective, cross-sectional	CADASIL (25) vs. HCs (14)	1.5 T; NR; tailored cervical PAC	• C1—C7 • 24 axial slices (contiguous)	• 2D GE ± MT pre-pulse (Gaussian, 1.5 kHz offset, 7.7 ms, 500°), FA = 20°	NR; NR; 5 mm thick; 792/10; no; NR	MTR, histogram peak, location	Semi-automatic, whole-cord, excluding voxels with MTR < 10%	• Rankin score	• No difference in MTR or histogram location • MTR peak height lower in CADASIL: P = 0.02 • MTR correlates with Rankin disability: r = − 0.4, P = 0.05	High (diagnostic), moderately high (correlation); coarse clinical data, results are NS if corrected
Rovaris et al. (2001a); prospective, cross-sectional; high	Migraine (16) vs. HCs (17)	Same as Rovaris et al. (2000)						• Presence/absence of aura	• No differences in mean MTR or histogram metrics	High; no group differences found, minimal clinical data
Rovaris et al. (2001b); prospective, cross-sectional	PPMS (91) vs. SPMS (36) vs. HCs (30)	Same as Rovaris et al. (2000)				MTR, histogram peak	Same as Rovaris et al. (2000)		• Mean MTR decreased vs. HCs: 42 vs. 46, P < 0.001 • Peak height decreased vs. HCs: 61 vs. 72, P = 0.001 • Peak height increased vs. SPMS: 61 vs. 57, P = 0.003 • No metric had univariate correlation with EDSS	Moderately high (diagnostic), moderately low (correlation); coarse clinical data, no correlations found
Filippi et al. (2002); prospective, cross-sectional	PPMS (26) vs. HCs (15)	1.5 T; Siemens; tailored cervical PAC	• C1–C7 • 24 axial slices (contiguous)	• 2D GE ± MT pre-pulse (Gaussian, 1.5 kHz offset, 7.7 ms, 500°), FA = 20°, NEX = 2	250 mm^2^; 256 × 256; 1 × 1 × 5 mm^3^; 640/12; no; NR	MTR, histogram peak, location	Semi-automatic, whole-cord, excluding voxels with MTR < 10%	• EDSS • fMRI brain activations	• Decreased MTR: 40 vs. 46, P < 0.001 • Decreased peak height: 62 vs. 112, P < 0.001 • Decreased peak location: 35 vs. 40, P = 0.003 • MTR does not correlate with EDSS • MTR metrics correlate moderately with fMRI activation of several motor areas	High; no correlations with EDSS, utility of correlations with brain fMRI activation is unclear
Rovaris et al. (2004); prospective, cross-sectional	CIS (45) vs. HCs (27)	Same as Rovaris et al. (2000)							• No significant differences in metrics vs. HCs • 3/45 subjects had mean MTR 2 SDs below mean of HCs	High; no group differences found
Fatemi et al. (2005); prospective, cross-sectional	AMN (17 total, 9 full AMN, 8 X-ALD hetero-zygotes) vs. HCs (10)	1.5 T; Philips; 2 element neck PAC	• C1–C3 • 32 axial slices (contiguous)	• 3D GE with MT pre-pulse (sinc, 15 ms, 5 offsets 10–63 kHz), FA = 7°	225 × 48 mm^3^; 256 × 256 × 32; 1 × 1 × 1.5 mm^3^; 50/13; no; NR	MTCSF	Manual, DCs	• EDSS • R, L 1st toe vibration • Standing balance test	• MTCSF increased in AMN (34) vs. X-ALD (30) vs. controls (27): P < 0.0001 • DC MTCSF correlates with EDSS (r = 0.62, P = 0.01), vib. Sense (r = 0.75, P = 0.002), and balance sway (r = 0.62, P = 0.01)	High (diagnostic), moderately high (correlation); manual ROI, DCs only
Agosta et al. (2006); prospective, cross-sectional	Neuro-borreliosis (Lyme Disease) (20) vs. HCs (11)	1.5 T; Siemens; tailored cervical PAC	• C1–C7 • 24 axial slices (contiguous)	• 2D GE ± MT pre-pulse (Gaussian, 1.5 kHz offset, 7.7 ms, 500°), FA = 20°	250 mm^2^; 256 × 256; 1 × 1 × 5 mm^3^; 640/12; no; NR	MTR	Semi-automatic, whole-cord, excluding voxels with MTR < 10%	• None	• No difference in cervical cord MTR	Moderately high; no group difference found
Rocca et al. (2006); prospective, cross-sectional	Isolated myelitis (24) vs. HCs (15)	1.5 T; Siemens; NR	• C1–C7 • 20 axial slices (gap NR)	• 2D GE ± MT pre-pulse (Gaussian, 1.5 kHz offset, 7.7 ms, 500°), FA = 20°	NR; NR; 5 mm thick; 640/12; no; NR	MTR	Semi-automatic, whole-cord, excluding voxels with MTR < 10%	• EDSS • 9 hole peg • Finger-tapping	• MTR decreased in myelitis vs. HCs: 36 vs. 41, P < 0.0001 • MTR decreased in cervical vs. thoracic myelitis: 35 vs. 37, P = 0.01 • No correlation between MTR and clinical measures • Various correlations between MTR and brain fMRI activations	High (diagnostic), moderately high (correlation); no correlations with clinical measures
Agosta et al. (2007b); prospective, cross-sectional	RRMS (18) vs. HCs (13)	1.5 T; Siemens; tailored cervical PAC	• C1–C7 • 20 axial slices (contiguous)	• 2D GE ± MT pre-pulse (Gaussian, 1.5 kHz offset, 7.7 ms, 500°), FA = 20°	180 mm^2^; 128 × 128; 1.4 × 1.4 × 4 mm^3^; 600/25; no; NR	MTR	Manual, GM (avoiding edge voxels)	• EDSS	• Decreased GM MTR: 23.5 vs. 24.8, P = 0.009 • GM MTR correlates with EDSS: r = − 0.48, P = 0.048	High (diagnostic), moderately high (correlation); coarse clinical data
Rovaris et al. (2008); prospective, cross-sectional	RRMS (23) vs. HCs (10)	Same as Rovaris et al. (2001b)						EDSS	No difference in metrics vs. HCs No correlation in metrics with brain T2w lesions	High; no group differences found, correlation with EDSS NR
Zackowski et al. (2009); prospective, cross-sectional	MS (42) vs. HCs (18)	3 T; Philips; 2-element surface PAC	• C2–C6 • 40 contiguous axial slices	• GE ± MT pre-pulse (sinc-Gauss, 1.5 kHz offset, 24 ms), FA = 9°, SENSE = 2	NR; NR; 0.6 × 0.6 × 2.25 mm^3^; 110/13; no; NR	MTCSF	Manual, 3 ROIs in each slice: DCs and R/L LCs; GM ROI in 5 slices at C2–3	• EDSS • Vibration • Posture sway • Ankle power • Walk speed	• MTCSF of LC (but not DC, GM) increased in MS vs. HCs: 0.55 vs. 0.50, P = 0.008 • MTCSF of DC correlates with vibration (r = 0.58, P < 0.001), sway (r = 0.32, P = 0.02), EDSS (r = 0.41, P < 0.05) • MTCSF of LC correlates with ankle strength (r = − 0.45, P = 0.003), walk speed (r = − 0.51, P < 0.001), and EDSS (r = 0.59, P < 0.05)	High; groups not age-matched, manual tract-specific ROIs
Cohen-Adad et al. (2011); prospective, cross-sectional	cSCI (14) vs. HCs (14)	3 T; Siemens; multi-channel head, neck, spine PACs	• C2–T2 • 52 axial slices, 0.4 mm gap	• 3D GE ± MT pre-pulse (Gaussian, 1.2 kHz offset, 10 ms)	230 mm^2^; 256 × 256; 0.9 × 0.9 × 2 mm^3^; 28/3.2; no; 10 min	MTR	Manual, 4 ROIs: ACs, DCs, L/R LCs; lesion levels skipped in cSCI	• ASIA motor and sensory scores	• Decreased MTR: 26 vs. 32, P < 0.0001 • MTR correlates with total ASIA score: r = 0.59, P = 0.04 • MTR of ACs/LCs more specifically predicts motor score (P = 0.03), dorsal region predicts sensory score (P = 0.03)	High (diagnostic), moderately high (correlation); manual tract-specific ROIs
Cohen-Adad et al. (2013); prospective, cross-sectional	ALS (29) vs. HCs (21)	Same as Cohen-Adad et al. (2011)						• ALSFRS-R • TMS motor threshold	• Reduction in MTR greatest at caudal levels • MTR not correlated with ALSFRS-R	High; manual tract-specific ROIs, groups not gender-matched
Oh et al. (2013a); prospective, cross-sectional	MS (124 total, 69 RRMS, 36 SPMS, 19 PPMS), no HCs	3 T; Philips; 2 element surface PAC	• C2–C6 • 30 axial slices, contiguous	• 3D GE T2*w EPI ± MT pre-pulse (1.5 kHz offset, sinc-Gauss shape), FA = 9°, SENSE = 2	NR; NR; 0.6 × 0.6 × 3 mm^3^; 121/12.5; no; NR	MTR	Automatic segmentation, whole-cord at C3–4 (11 slices)	• EDSS • MSFC	• MTR decreased in high vs. low EDSS in high lesion count subjects (P = 0.003) • No difference in MTR in high lesion count subjects	Moderately high; diagnostic accuracy NR
Oh et al. (2013b); prospective, cross-sectional	MS (129 total, 74 RRMS, 36 SPMS, 19 PPMS) vs. HCs (14)	Same as Oh et al. (2013a)						• EDSS • Hip flexion power • Vibration	• Decreased MTR in total MS vs. HCs: 30 vs. 31, P = 0.04 • Decreased MTR in progressive MS vs. RRMS: 0.28 vs. 0.31, P < 0.001 • MTR correlates with EDSS (P = 0.02) and vibration (P = 0.05) in multivariate regression	Moderately high (diagnostic), moderately low (correlation); no diagnostic accuracy
El Mendili et al. (2014); prospective, longitudinal	ALS (29), no HCs	3 T; Siemens; neck/spine coil; NR	• C2–T2 52 axial slices, gap NR	• 3D GE ± MT pre-pulse (Gaussian, 1.2 kHz offset, 10 ms)	230 mm^2^; 256 × 256; 0.9 × 0.9 × 2 mm^3^; 28/3.2; no; 5 min	MTR	Manual, 4 ROIs: ACs, DCs, L/R LCSTs	• ALSFRS-R • Muscle power • FU at 1 year	• MTR at 1 year decreased from baseline: 30 vs. 33, P = 0.003 • No correlation between change in MTR and change in clinical scores • Baseline MTR not predictive of 1 year outcome	Moderately high; no correlation/prediction found, manual ROIs
Kearney et al. (2014); prospective, cross-sectional	MS (133 total, 22 CIS, 29 RRMS, 28 SPMS, 28 PPMS) vs. HCs (26)	3 T; Philips; 16 channel neuro-vascular coil	• C1–C7 • 22 axial slices	• 3D spoiled GE ± MT pre-pulse (Gaussian, 1 kHz offset, 16 ms), FA = 20° NEX = 2, SENSE = 2	180 × 240 mm^2^; 240 × 320; 0.8 × 0.8 × 5 mm^3^; 36/3.5,5.9; no; NR	MTR	Semi-automatic, outer cord, WM, GM at C2–3 (3 slices)	• EDSS • 25-foot TWT • 9 hole peg • ASIA motor, sensory	• WM MTR decreased in all subgroups vs. controls (P < 0.05) • MTR correlates with EDSS in GM (r = − 0.34), WM (r = − 0.32), outer cord (r = − 0.41) • Cord CSA showed stronger correlations with all clinical measures (e.g. R = − 0.60 with EDSS) than MTR	Moderately high (diagnostic), moderately low (correlation); CSA outperformed MTR
Kearney et al. (2015); prospective, cross-sectional	MS (92 total, 34 RRMS, 29 SPMS, 29 PPMS) vs. HCs (28)	Same as Kearney et al. (2014)					Semi-automatic, whole-cord, lesions	• EDSS • MSFC • 9 hole peg • PASAT • TWT	• Whole-cord MTR decreased in SPMS (P = 0.01) and PPMS (P = 0.004) vs. HCs • No difference in whole-cord or lesion MTR between subgroups • MTR not independently associated with disability (CSA, lesion load were stronger multivariate factors)	Moderately high (diagnostic), moderately low (correlation); no correlations with disability found
Oh et al. (2015); prospective, cross-sectional	MS (102 total, 66 RRMS, 24 SPMS, 12 PPMS) vs. HCs (11)	Same as Oh et al. (2013a)					Same as Oh et al. (2013a)	• EDSS • MSFC • Vibration • Hip flexion • OCT of retina	• MTR not different between total MS vs. HCs • MTR decreased in progressive MS vs. RRMS: P < 0.001 • MTR not correlated with retinal layer measures • MTR not correlated with clinical measures	Moderately high (diagnostic), moderately low (correlation); no group difference vs. HCs, no correlations found

**Table 6:** Summary of MWF studies.

Authors (year); design	Subjects	B_0_; vendor; coil	Anatomical region/position	MWF acquisition	FOV; matrix; voxel size; TR/TE (ms); cardiac gating; AT	MWF metrics	ROI	Clinical measures	Key results	Risk of bias; key barriers to translation
Laule et al. (2010); prospective, longitudinal	PPMS (24) vs. HCs (24)	1.5 T; GE; standard head coil	• C2–C3 • Single axial slice	• T2w 32-echo sequence (spacing 10 ms) with IR (TI = 1200 ms), NEX = 2	220 mm^2^; 256 × 128; 0.9 × 0.9 × 5 mm^3^; 3000/10 (32 echoes); no; 30 min	MWF (ratio of 15–40 ms signal to total); MRI repeated at 1 year, 2 years	Manual, whole-cord	• EDSS • FU EDSS at 1 year, 2 years	• NS difference in MWF vs HCs: 0.23 vs. 0.25, P = 0.12 • 10% decrease in MWF in PPMS over 2 years (P = 0.01) • Baseline MWF not correlated with EDSS, not predictive of decline • No effect of demyelination treatment on MWF	High; no group difference vs. HCs, coarse clinical data, no correlations or successful prediction found

**Table 7:** Summary of MRS studies.

Authors (year); design	Subjects	B_0_; vendor; coil	Anatomical region/position	MRS acquisition	Voxel size; TR/TE (ms); cardiac gating; AT	MRS metrics	Clinical measures	Key results	Risk of bias; key barriers to translation
Ciccarelli et al. (2007); prospective, longitudinal	MS (14 acute, lesion at C1–C3) vs. HCs (13)	1.5 T; GE; saddle coil	• Single voxel, C1–C3	• PRESS • Sat bands (NR) • NSA = 192 (w CHESS) • Shim method: NR • Phantom scanned using same voxel	6 × 8 × 50 mm^3^ (variable to fit cord); 3 heartbeats/30; yes (delay NR); NR	Absolute values and ratios for: NAA, Cre, Cho, Myo	• EDSS • 9-hole peg • 25-foot TWT • MSWS-12 • FU: EDSS at 3–6 months	• Decreased NAA: 4.1 vs. 6.7, P < 0.0001 • No difference in Myo, Cho, Cre • Correlations found with EDSS: Myo (r = 0.64, P = 0.02), Cho (r = 0.65, P = 0.01), Cre (r = 0.75, P = 0.003) • Cre correlates with upper limb metrics (P < 0.05) and MSWS-12	High (diagnostic), moderately high (correlation); no prediction of FU EDSS, high variance of metrics
Holly et al. (2009); prospective, cross-sectional	CSM (21) vs. HCs (13)	1.5 T; Siemens; neck coil	• Single voxel, C2	• PRESS • NSA = 256 • Shim method: manual (18–28 Hz)	10 × 10 × 20 mm^3^ (variable to fit cord); 1500 or 3000/30; no; 3–5 min shimming + 3 min 40 s	NAA/Cre, Cho/Cre, presence of Lac peak	• mJOA	• Decreased NAA/Cre: 1.27 vs. 1.83, P < 0.0001 • No difference in Cho/Cre • No correlation between NAA/Cre and mJOA • 7/21 CSM patients had lactate peak vs. no controls, P < 0.05	High (diagnostic), moderately high (correlation); boxplot shows low SE/SP
Ciccarelli et al. (2010a); prospective, cross-sectional	MS (14, 6 min within lesion onset at C1–C3) vs. HCs (13)	Same as Ciccarelli et al. (2007)				ResNAA (NAA not explained by AD, CSA parameters)	Same as Ciccarelli et al. (2007)	• Decreased NAA: 4.2 vs. 5.9, P = 0.03 • ResNAA correlates with EDSS (R^2^ = 0.5, P = 0.03), TWT (R^2^ = 0.4, P = 0.02), and MSWS-12 (R^2^ = 0.4, P = 0.01)	High; high variance of metrics, requires MRS, DTI in same ROI
Ciccarelli et al. (2010b); prospective, longitudinal	Same as Ciccarelli et al. (2007)					Absolute NAA; FU MRS studies at 1, 3, 6 months	Same as Ciccarelli et al. (2007)	• Increase in NAA from 1 month to 6 months in patients that recover following acute MS: P = 0.001 • Baseline NAA and NAA change over 1^st^ month not predictive of outcome	High; weak results for correlation and prediction
Marliani et al. (2010); prospective, cross-sectional	RRMS (15) vs. HCs (10)	3 T; GE; 8-channel spine PAC (upper 4 elements)	• Single voxel, C2–C3	• PRESS • NSA = 400 (CHESS), 16 (no water suppression) • Automatic shimming	7 × 9 × 35 mm^3^ (variable); 2000/35; no; 14 min	NAA/Cre, NAA/Cho, Cho/Cre, Myo/Cre	• EDSS	• All metabolite ratios significantly altered in RRMS (P = 0.002 to 0.04) • No metabolite ratios correlate with EDSS	High; no correlation with EDSS found, diagnostic accuracy NR
Carew et al. (2011a); prospective, cross-sectional	ALS (14) vs. HCs (16)	3.0 T; Siemens; head/neck/spine PACs	• C1–C2	• PRESS • NSA = 256 (CHESS), 4 (no water suppression) • Automatic shimming with B_0_ mapping	8 × 5 × 35 mm^3^; 2000/35; no; 12 min	Ratios between Cho, Myo, NAA, Cre	• ALSFRS-R • FVC	• Decreased NAA/Cre: 0.75 vs. 1.25, P = 0.0007 • Decreased Cho/Cre: 0.40 vs. 0.50, P = 0.007 • NAA/Myo correlates with FVC: r = 0.66, P = 0.01 • Metrics not significantly correlated with ALSFRS-R	High; 4/30 subjects excluded due to technical problems, no correlation with ALSFRS-R found
Carew et al. (2011b); prospective, cross-sectional	SOD1 (24) vs. ALS (23) vs. HCs (29)	Same as Carew et al. (2011a)					• None (asymptomatic population)	• SOD1 vs. HCs shows decreased NAA/Cre (P = 0.001), decreased Myo/Cre (P = 0.02) • SOD1 vs. ALS shows increased NAA/Cho (P = 0.002)	High; 12 metric calculations excluded due to technical issues
Bellenberg et al. (2013); prospective, longitudinal	MS (22) vs. HCs (17)	1.5 T; Siemens; head/neck coil PAC	• Single voxel, C3–C5 (variable, to include MS lesions)	• PRESS • 8 adjacent sat bands • NSA = 128 (CHESS), 16 (no water suppression) • Shim method: NR	8 × 10 × 40 mm^3^; 1500/30; yes (300 ms delay); AT NR	Absolute values and ratios for: NAA, Cre, Cho, Myo; MRI study repeated at 1 year FU	• EDSS • Max. walking distance • MSFC • 25-foot TWT • 9-hole peg • FU at 1 year, 2 years	• Decreased NAA, NAA/Cre (P < 0.01), Cho/Cre (P = 0.026) • Increased Myo (P = 0.001), Myo/Cre (P = 0.002) • NAA correlates with age: r = − 0.482, P = 0.003 • No correlation with clinical measures • No significant changes in MRS metrics over 1 year FU • MS patients that worsened after 1 year had lower baseline NAA/Cre (P = 0.024) and higher Cho (P = 0.021)	High (diagnostic, prognostic), moderately high (correlation); no correlation found, weak prediction of outcome
Ikeda et al. (2013); prospective, longitudinal	ALS (19) vs. HCs (20)	1.5 T; Siemens; NR	• Single voxel, C1–C3	• PRESS • NSA = 400 (CHESS) • Shim method: automatic	6 × 8 × 40 mm^3^; 1500/50; no; 15 min	NAA/Cre, Cho/Cre, Myo/Cre, NAA/Myo	• ALSFRS-R • FVC • EMG • Data captured 6 min prior, 6 min after	• Decreased NAA/Cre, NAA/Myo, increased Myo/Cre: ALS vs. HCs and with vs. without EMG denervation (P < 0.01) • NAA/Cre and NAA/Myo correlate with ALSFRS-R: r = 0.79, P < 0.01 and ρ = 0.76, P < 0.01 respectively • NAA/Cre and NAA/Myo predict decline of ALSFRS-R: r = − 0.70, P < 0.01 and ρ = − 0.78, P < 0.01	High (diagnostic, prognostic), moderately high (correlation); long acquisition time difficult for ALS population
Salamon et al. (2013); prospective, cross-sectional	CSM (21 total, 11 with T2w-HI, 10 without) vs. HCs (11)	3 T; Siemens; NR	• Single voxel, C2	• PRESS • NSA = 256 (CHESS), 4 (no water suppression) • 6 sat bands • Shim method: manual	7 × 7 × 35 mm^3^; 2000/30; no; NR	NAA/Cre, Glu/Cre, Cho/Cre, Myo/Cre, (Lip + Lac)/Cre, Cho/NAA	• mJOA	• Cho/NAA increased in CSM (P < 0.01) • Cho/NAA correlates with mJOA: R = − 0.45, P < 0.01	High; coarse clinical data, age/gender of HCs NR
Taha Ali and Badawy (2013); prospective, cross-sectional	CSM (24) vs. HCs (11)	1.5 T; Siemens; neck circular surface coil	• Single voxel, C2	• PRESS • NSA = 512 (CHESS) • Multiple very selective sat bands placed	10 × 10 × 15–20 mm^3^; 2000/36; yes, 4 min 54 s	NAA, Cho, Cre, Lac, NAA/Cre, Cho/Cre	• None	• NAA/Cr decreased: 1.34 vs. 1.82, P < 0.0001 • Lactate peak present in 9/24 CSM subjects, no HCs	High; no clinical data, diagnostic accuracy only provided for lactate

**Table 8:** Summary of fMRI studies.

Authors (year); design	Subjects	B_0_; vendor; coil	Anatomical region/position	fMRI acquisition	FOV; matrix; voxel size; TR/TE (ms); cardiac gating; AT	fMRI metrics	ROI	Clinical measures	Key results	Risk of bias; key barriers to translation
Stroman et al. (2004); prospective, cross-sectional	cSCI (27) vs. HCs (15)	1.5 T; GE; spine PAC	• T11-conus • 5 axial slices, mid-disk or mid-VB	• Single-shot FSE • PD-weighted, SEEP contrast • 3 sat bands: ant, L, and R • 8.25 s/volume • Block-design, thermal stimulus (10C, 32C) to legs	120 × 120 mm^2^; 128 × 128; 0.9 × 0.9 mm^2^, thickness NR; 8250/34; no; NR	Activation maps; co-registered with template, group activation for voxels active in ≥ 3 subjects	L1–S1 cord	• AIS grade	• Activation in lumbar cord seen in all cSCI subjects • Complete SCI subjects showed decreased ipsilateral dorsal activation and increased bilateral ventral activation (P values NR)	High; minimal clinical data, activations not corrected, only qualitative analysis of group activations
Agosta et al. (2008a); prospective, cross-Sectional	RRMS or SPMS (24) vs. HCs (10)	1.5 T; Siemens; Phased-array spine coil	• C5–C8 cord • 9 axial slices (mid-VB or mid-disk), gap adjusted to fit	• Multishot Turbo SE, FA = 120° • PD-weighted, SEEP contrast • 2 sat bands (ant. and post.) • 13 s/volume • Block-design, tactile stimulus to right hand	100 × 100 mm^2^; 256 × 244; 0.4 × 0.4 × 7 mm^3^; 2850/11; no; NR	Frequency of activation; mean SI change (active voxels)	Manual, 5 regions (R ant., L ant., R post., L post., central)	• EDSS	• Increased mean SI change (active voxels): 3.4% vs. 2.7%, P = 0.03 • Decreased frequency of ipsilateral activation: P = 0.003 • Decreased frequency of posterior activation: P = 0.02	High; coarse clinical data, activations not corrected, correlation with EDSS NR
Agosta et al. (2008b); prospective, cross-Sectional	RRMS or SPMS (25) vs. HCs (12)	Same as Agosta et al. (2008a)							• Increased mean SI change (active voxels): 3.9% vs. 3.2%, P = 0.02 • Mean SI change correlates with mean cord FA: r = − 0.48, P = 0.04 • Average SI change correlates with cord FA: r = − 0.48, P = 0.04	High; coarse clinical data, activations not corrected, correlation with EDSS NR
Agosta et al. (2009b); prospective, cross-Sectional	PPMS (23) vs. HCs (18)	Same as Agosta et al. (2008a)							• Increased mean SI change (active voxels): 3.3% vs. 2.6%, P = 0.05 • Decreased frequency of posterior activation (P < 0.001) • Mean SI change correlates with mean cord FA: r = − 0.58, P = 0.001	High; coarse clinical data, activations not corrected, correlation with EDSS NR
Valsasina et al. (2010); prospective, cross-sectional	MS (49 total, 30 RRMS, 19 SPMS) vs. HCs (19)	Same as Agosta et al. (2008a)							• RRMS (P = 0.05) and SPMS (P = 0.02) had increased cord activation • Severe disability corresponded to increased activation vs. controls (P = 0.004) and mild disability (P = 0.04)	High; coarse clinical data, activations not corrected, correlation coefficients NR
Cadotte et al. (2012); prospective, cross-sectional	cSCI (18) vs. HCs (20)	3.0 T; GE and Siemens; NR	• Brainstem and C1–T1 • 9 sagittal slices, contiguous	• ssFSE (HASTE) multi-echo, partial Fourier • PD-weighted, SEEP contrast • 9 s/volume • Thermal (44C) stimulus, L/R above and below injury	280 × 210 mm^2^; 192 × 144; 1.5 × 1.5 × 2 mm^3^; 9000/38; no; 7 min 12 s	Number of positive and negative active voxels per dermatome; connectivity analysis	Manual, 4 quadrants	• ASIA sensory score	• Increased number of active voxels in incomplete cSCI in dermatome of normal sensation • Number of active voxels correlates with degree of sensory impairment: R^2^ = 0.93, P < 0.001 • Increased number of intraspinal connections in cSCI vs. HCs	High; sensory-only paradigm, requires thermal stimulator
Rocca et al. (2012); prospective, cross-sectional	MS (35 total, 20 with fatigue, 15 without) vs. HCs (20)	Same as Agosta et al. (2008a)						• EDSS • Fatigue Severity Scale	• No difference in number of active voxels between MS groups or HCs • MS without fatigue had more distributed activation outside ipsilateral dorsal quadrant vs. MS with fatigue and HCs (P < 0.05) • Bilateral recruitment correlated with severity of fatigue: r = − 0.34, P = 0.04	High; activations not corrected (no activations in 30% of subjects at p < 0.001), altered recruitment not clearly defined
Valsasina et al. (2012); prospective, cross-sectional	Progressive MS (34 total, 18 SPMS, 16 PPMS) vs. HCs (17)	Same as Agosta et al. (2008a)							• Activation increased vs. HCs: P = 0.003 • Activation increased in SPMS vs. PPMS: P = 0.05 • No correlation between activation and EDSS	High; coarse clinical data, activations not corrected, no correlation with EDSS found

**Table 9:** Summary of studies by clinical pathology.

Clinical pathology	Number of studies by imaging technique						Key findings		
ROI DTI	DTI FT	MT	MWF	MRS	fMRI	Diagnostic utility	Biomarker utility (correlation with disability)	Predictive utility
ALS	7		2		3		• FA decreased (7/7 studies), specifically in LCSTs (4/4 studies) • MTR (in LCSTs) was decreased in ALS (1 study) • NAA decreased in ALS (3/3 studies)	• FA correlated with ALSFRS (r = − 0.55–0.74, R = 0.38, 4/6 studies) • NAA/Cre correlates with ALSFRS (r = 0.79, 1/2 studies) and FVC (r = 0.66, 1 study) • FA, MD changes over 1 year not correlated with change in ALSFRS (2/2 studies) • MTR does not correlate with ALSFRS (1 study)	• FA predicted ALSFRS at 1 year (1 study) • NAA/Cre and NAA/Myo predict ALSFRS at 1 year (r = − 0.70–0.78, 1 study)
aSCI	3						• MD decreased (2/3 studies) • FA decreased (2/3 studies)	• FA correlates with one or more components of ASIA motor score (2/2 studies)	
CM	3	3					• FA decreased and MD increased at MCL (2/3 studies) • FA had higher SE (73%) and SP (100%) than T2w-HI (1 study)	• No correlation of FA, MD, FT with JOA (1 study)	• FA, MD did not predict JOA outcome (1 study)
cSCI	4		1			2	• FA decreased above (4/4 studies) and below (3/3 studies) injury site • FA at lesion correlates with ASIA motor score (r = 0.67, 1 study) • FA, RD outside lesion correlates with ASIA motor/sensory scores (r = 0.66–0.74, 1 study) • MTR decreasd above/below injury (1 study) • fMRI shows increased bilateral activation in cSCI vs. HCs (2/2 studies)	• MTR correlates with ASIA motor/sensory score (r = 0.59, 1 study) • Number of active voxels correlates with sensory impairment (R = 0.96, 1 study)	
CSM	18	5			3		• FA had SE = 72–95%, SP = 50–100% to detect myelopathy (4 studies) • MD had SE = 13–100%, SP = 50–80% to detect myelopathy (3 studies) • OE had SE = 81%, SP = 67% to detect myelopathy (1 study) • FA reduced at compressed level (12/12 studies), above compression (2/5 studies), and below compression (1/3 studies) • MD increased at compressed level (8/10 studies), above compression (1/4 studies), and below compression (1/3 studies) • MK decreased in overall cord (1 study) • NAA/Cre reduced (2/3 studies), Cho/NAA increased (1 study) • Lactate peak present in 33% of subjects (1 study)	• FA correlates with JOA/mJOA (r = 0.48–0.88, R = 0.57–0.64, 5/5 studies) • SD(θ) correlates with mJOA (R = 0.64, 1 study) • Tractography pattern only correlated with clinical scale (JOA/Nurick) in 1/3 studies • NAA/Cre ratio not correlated with mJOA (1 study) • Cho/NAA correlated with mJOA (R = − 0.45, 1 study)	• FA predicts improvement on NDI (r = − 0.61) but not mJOA (1 study) • FA predicts mJOA recovery ratio > 50% (P = 0.03, 1 study)
MS	19	3	16	1	5	5	• FA has SE = 87%, SP = 92% for diagnosis (1 study) • FA reduced in whole-cord (11/12 studies), NAWM (6/8 studies), and in lesions (3/3 studies) • MD increased in whole-cord (7/10 studies), NAWM (2/5 studies), lesions (2/3 studies) • RD increased in whole-cord (4/6 studies) • FA decreased in progressive MS vs. RRMS (4 studies) • MK decreased in NAGM and lesions (1 study) • MTR decreased in whole-cord (8/11), WM (2/2), GM (1/2 studies) • MTR decreased in progressive MS vs. RRMS (2/3 studies) • MTCSF increased in WM (1 study) • MWF not different vs. HCs (1 study) • Decreased NAA (4/4 studies) • Increased number of active voxels (2/6 studies) • Increased mean SI change in active voxels (3/3 studies) • Increased distribution of activation outside expected ipsilateral dorsal horn (2/2 studies)	• FA correlates with EDSS (r = − 0.37–0.51, R = − 0.60, 7/15 studies), TWT (R = 0.70, 1 study) • FA of LCST correlates with MEPs (r = − 0.93, 1 study) • MD correlates with EDSS (r = 0.37, 3/13 studies) • RD correlates with EDSS (R = 0.7, 4/8 studies) and TWT (R = − 0.6, 1 study) • MK does not correlate with EDSS (1 study) • MTR correlates with EDSS (r = − 0.25–0.48, 6/15 studies) • MTCSF of LCs correlates with EDSS (r = 0.59), walk speed (r = − 0.51), ankle strength (r = − 0.45) (1 study) • MTCSF of DCs correlates with EDSS (r = 0.59), vibration (r = 0.58), postural sway (r = 0.32) (1 study) • Change in MWF over 1 year, 2 years not correlated with change in EDSS (1 study) • NAA does not correlate with EDSS (5 studies) • Number of active voxels correlates with EDSS (1/3 studies)	• FA predicts EDSS at 6 months–3 years FU (r = − 0.40, 2/2 studies) • RD predicts EDSS, 9 hole peg, and TWT at 6 months FU (P < 0.05, 1 study) • MWF not predictive of EDSS at 1 year, 2 years (1 study) • NAA predicts decrease in EDSS at 6 months–1 year FU (1/2 studies)
Myelitis	2	2	1				• Diagnostic utility: • FA decreased at lesion site (3/3 studies) • MTR decreased at lesion site (1 study)	• FA, RD correlate with EDSS (P < 0.0001) and 9 hole peg (P < 0.0001) (1 study) • FA correlates with sensory score (r = − 0.40, 1 study) • MTR does not correlate with clinical measures EDSS, 9 hole peg, finger-tapping (1 study)	
NMO	2						• FA decreased in NAWM (2/2) and lesions (1/1) • FA decreased in NAWM vs. MS (1 study) • MD increased in NAWM (1/1) and lesions (1/1)	• FA correlates with EDSS (r = − 0.80, 1 study)	
Syringo-myelia	1	2					• FA decreased at syrinx vs. HCs (2/2 studies) • FA decreased between symptomatic vs. asymptomatic subjects (1 study) • FA not different above/below syrinx (1 study)	• FA correlates with thermal sensation in 1/2 ROIs (r = − 0.63, 1/2 studies) • FA (r = − 0.64, P = 0.02) and number of FT fibers (r = − 0.75, P = 0.02) correlate with average daily pain scores (1 study)	

**Table 10:** Evidence summary.

Key question	Specific finding	Quality of evidence		
Baseline	Upgrade/downgrade	Final
1) *Diagnostic utility*: Does the MRI technique provide metrics that demonstrate group differences or improved sensitivity/specificity in the diagnosis of spinal pathologies?	FA is decreased in terms of group differences between patients and healthy controls in the clinical conditions ALS, CSM, myelitis, MS, NMO, and SCI	Low	None	Low
MD, RD, MK, MTR, MTCSF, and NAA demonstrate group differences between patients and healthy controls in various clinical conditions	Low	Downgrade: inconsistency (1)	Very low
AD, SD(θ), OE, tractography pattern, MWF, and fMRI metrics demonstrate group differences between patients and healthy controls in various clinical conditions	Low	Downgrade: inconsistency (1), imprecision of estimates (1)	Insufficient
Quantitative metrics based on state-of-the-art MRI techniques can be used for diagnosis with high diagnostic accuracy (sensitivity and specificity)	Low	Downgrade: inconsistency (2)	Insufficient
2) *Biomarker utility*: Does the advanced MRI technique generate quantitative metrics that correlate with neurological/functional impairment and/or show longitudinal changes that correlate with changes in impairment in spinal pathologies?	FA shows moderate correlation with clinical impairment in a number of clinical conditions: ALS, CSM, MS, myelitis, NMO, and SCI	Low	Upgrade: dose–response gradient	Moderate
MD, RD, MTR, MTCSF, NAA are weak-moderate biomarkers for clinical impairment in various clinical conditions	Low	Downgrade: inconsistency (1), imprecision of estimates (1)	Insufficient
3) *Predictive utility*: Does the advanced MRI technique generate metrics that predict neurological, functional, or quality of life outcomes in spinal pathologies?	FA, RD, and NAA are predictive of outcome in MS, ALS, and CSM	Low	Downgrade: inconsistency (1), imprecision of estimates (1)	Insufficient
